# Social determinants of health and *Helicobacter pylori* infection prevalence: a systematic review and meta-analysis

**DOI:** 10.3389/fpubh.2025.1703158

**Published:** 2026-01-13

**Authors:** Hengmin Wang, Zhenyu Wang, JianGen Yu, Jianan Jin

**Affiliations:** 1 Department of Gastroenterology, Shulan (Hangzhou) Hospital, Shulan International Medical College, Zhejiang Shuren University, Hangzhou, China; 2 Graduate School, Shaoxing University, Shaoxing, China; 3 Department of the College, The First People's Hospital of Xiaoshan District, Hangzhou, China; 4 Department of Gastroenterology, Hangzhou Xiaoshan Second People’s Hospital, Hangzhou, China

**Keywords:** *Helicobacter pylori*, social determinants of health, prevalence, systematic review, meta-analysis

## Abstract

**Objective:**

*Helicobacter pylori* infection represents a major public health challenge. While its transmission risk is potentially influenced by social determinants of health (SDoH), existing evidence remains fragmented. This study aimed to systematically evaluate the associations between various SDoH and *Helicobacter pylori* infection risk under a unified mapping framework and to explore the major sources of heterogeneity.

**Methods:**

We systematically searched PubMed, EMBASE, and Web of Science from inception to July 2025. Observational studies in human populations were included. SDoH were standardized into binary categories based on pre-defined rules. The prevalence of *Helicobacter pylori* and crude odds ratios (cORs) were pooled separately using random-effects models (Hartung–Knapp method with Paule–Mandel τ^2^ estimator). The Haldane–Anscombe correction was applied for zero cells. Pre-specified subgroup and exploratory meta-regression analyses were performed, and publication bias was assessed.

**Results:**

A total of 57 studies (*n* = 387,956 participants) were included. The overall pooled prevalence of *Helicobacter pylori* infection was 43% (95% CI: 36–50%), with considerable between-study heterogeneity (*I*^2^ = 99.6%). Subgroup analysis revealed significant variation across diagnostic methods (stool antigen test: 52%; urea breath test: 46%). In the analysis of SDoH associations, most adverse SDoH were positively associated with *Helicobacter pylori* infection. Household overcrowding (OR = 1.38, 95% CI: 1.03–1.85) and occupational instability (OR = 1.23, 95% CI: 1.05–1.45) showed statistically significant associations. Although the main analysis for education level was non-significant, a clear gradient was evident: the effect size for low versus high education (OR = 3.49) was stronger than that for low versus medium education (OR = 1.60). Meta-regression identified geographical region as the only stable source of heterogeneity.

**Conclusion:**

SDoH exert a significant influence on *Helicobacter pylori* infection risk, with household overcrowding, the educational gradient, and occupational instability being the most critical factors. In high-burden regions, public health strategies should prioritize alleviating housing overcrowding, enhancing education, and providing employment support to optimize primary prevention and screening strategies for *Helicobacter pylori*.

**Systematic review registration:**

https://www.crd.york.ac.uk/PROSPERO/view/CRD420251102412, CRD420251102412.

## Introduction

*Helicobacter pylori* (*H. pylori*) infection is one of the most prevalent chronic infectious diseases worldwide. A global study indicated that the infection rate among adults from 2015 to 2022 was approximately 43.9% (1). Currently, *Helicobacter pylori* infection has been strongly associated with gastrointestinal disorders, such as peptic ulcer disease and autoimmune gastritis (2, 3). In the 1990s, the World Health Organization classified *Helicobacter pylori* as a Group I carcinogen. Supporting this, a prospective cohort study involving 512,715 participants further confirmed that *H. pylori* infection is responsible for nearly 80% of non-cardia gastric cancer cases and over 60% of cardia gastric cancer cases annually in China (4). Recent updated analyses also show that although *Helicobacter pylori* infection has declined in some countries, its burden remains substantial in many low- and middle-income regions (5). Eradication therapy serves as the clinical cornerstone for managing *Helicobacter pylori* infection and preventing its associated diseases. However, at the population level, the prerequisite for achieving this goal is the efficient identification of infected individuals. In resource-limited settings, implementing risk-stratified precision screening, rather than untargeted population-wide screening, represents a more cost-effective public health strategy (6, 7). Therefore, identifying and quantifying key risk factors for infection, particularly modifiable social determinants, is crucial for developing effective screening strategies.

Social determinants of health (SDoH) refer to the social and environmental conditions throughout an individual’s life course, as well as the broader forces—such as economic systems, social policies, and development agendas—that shape these conditions (7). They encompass multiple dimensions, including socioeconomic status, education, healthcare access, neighborhood and built environment, and social and community contexts (8). Substantial evidence indicates that SDoH contribute to the distribution of and inequalities in both infectious diseases and chronic non-communicable diseases. This occurs through mechanisms such as differential exposures, chronic stress, health-related behaviors, and disparities in access to healthcare (9–13).

As one of the most common chronic infections, *Helicobacter pylori* infection is also significantly influenced by SDoH. Several studies to date have suggested that factors such as household crowding, unsafe drinking water and sanitation, and lower education and socioeconomic status are associated with a higher risk of *Helicobacter pylori* infection (14, 15). However, findings have been inconsistent across different regions, age groups, and diagnostic methods (e.g., serology, urea breath test [UBT], stool antigen), with considerable variation in effect sizes, indicating possible context-dependency and methodological heterogeneity. This inconsistency may stem, on the one hand, from real-world differences, and on the other, from variations in study design and measurement approaches. Notably, current research also lacks comprehensive studies examining *Helicobacter pylori* infection risk within a unified SDoH framework. Against this background, we therefore conducted a systematic review and meta-analysis. Under predefined rules, we uniformly mapped SDoH dimensions, pooled summary estimates of the association between different SDoH factors and the prevalence/risk of *Helicobacter pylori* infection, and performed stratifications by region, age group, and diagnostic method. Additionally, we assessed potential sources of heterogeneity through publication bias tests and (exploratory) meta-regression. This study aims to provide evidence to support decision-making for targeted screening and prevention strategies for *Helicobacter pylori*.

## Methods

This systematic review and meta-analysis were conducted in accordance with the Preferred Reporting Items for Systematic Reviews and Meta-Analyses (PRISMA 2020) statement (16). The study protocol was registered with the International Prospective Register of Systematic Reviews (PROSPERO), registration number CRD420251102412. The full registration record is available at: https://www.crd.york.ac.uk/PROSPERO/recorddashboard.

### Search strategy

We systematically searched PubMed, EMBASE, and Web of Science from their inception to July 11, 2025. The complete search strategy is detailed in Supplementary Table 1. The search combined Medical Subject Headings (MeSH) and free-text terms covering the key concepts of SDoH, *Helicobacter pylori*, and risk factors. All retrieved records were imported into EndNote 20 for deduplication, which was performed by matching authors, titles, publication years, and DOI/PMID. Titles and abstracts were screened for eligibility. Full texts of potentially relevant studies were retrieved and assessed. Furthermore, the reference lists of all included studies were manually searched to identify any additional pertinent publications.

### Eligibility criteria

Studies meeting all the following criteria were included as follows: (1) observational design (cross-sectional, cohort, or case–control); (2) conducted in human populations; (3) reported at least one social determinant of health (SDoH); (4) provided complete data for either (a) *Helicobacter pylori* prevalence/positive rate (or data allowing its calculation) or (b) a measure of association between an SDoH and *Helicobacter pylori* infection status [e.g., odds ratio (OR), risk ratio (RR), prevalence ratio (PR) with 95% confidence interval (CI), or raw data allowing calculation of an effect size]; and (5) were published in English.

Studies were excluded for the following reasons: (1) non-original research (e.g., reviews, systematic reviews/meta-analyses, editorials, letters, case reports/series); (2) incomplete reporting of essential data (e.g., effect estimates, raw frequencies, prevalence); (3) animal or *in vitro* studies; (4) duplicate or overlapping populations (for which only the report with the most complete information, largest sample size, most comprehensive adjustments, or most recent publication was retained). The specific definitions, measurement methods, and reclassification rules for each SDoH are detailed in Supplementary Tables S2, S3.

### Data extraction

Data extraction was performed independently by two investigators using a pre-piloted, standardized form. The following information was extracted from each included study:

1. Publication Details: First author, publication year, country/region, study setting (community/school/healthcare facility), and study period.
2. Study Design and Sample: Study type (cross-sectional/cohort/case–control), sample size, age group (children/adults/mixed), sex distribution, and race/ethnicity.
3. Exposure (SDoH): Specific SDoH indicator, its definition and measurement method, grouping categories and thresholds, and the reference group (e.g., urban vs. rural, favorable vs. unfavorable crowding).
4. Outcome (*Helicobacter pylori*): Diagnostic method (^13^C/^14^C-UBT, stool antigen test, rapid urease test [RUT], histology/culture, polymerase chain reaction (PCR), serology), positivity criteria, number of positive cases, and total number tested.
5. Effect Measures: The reported effect estimate (e.g., OR, RR) and its 95% CI for the SDoH-*Helicobacter pylori* association (adjusted estimates were prioritized for extraction). For the purpose of consistent meta-analysis, this study exclusively used crude odds ratios (cORs) calculated from original 2 × 2 tables for synthesizing associations; adjusted effect estimates (aORs) were not collected or pooled.

For each study and each comparable exposure-control pair, the OR and its 95% CI were calculated using the logit method. The Haldane-Anscombe continuity correction (adding 0.5 to all cells) was applied to handle zero cells. To ensure cross-study comparability, all SDoH variables were uniformly re-mapped into binary categories (e.g., favorable vs. unfavorable; rural vs. urban) according to pre-specified rules (see Supplementary Tables S2, S3). The crude OR (cOR) and its 95% CI were then calculated based on these standardized 2 × 2 tables. Detailed mapping rules and subgroup classifications are provided in the Supplementary materials.

### Quality assessment

The methodological quality of cohort and case–control studies was assessed using the Newcastle-Ottawa Scale (NOS). For cross-sectional studies, the adapted NOS-xs scale was used for assessing the quality of association analyses, and the NOS-xs2 scale was used for assessing the quality of prevalence estimates. Studies with a score of ≥6 (for NOS/NOS-xs) or ≥3 (for NOS-xs2) were considered to have acceptable quality and were included in the main analysis (17). The specific tools, scoring criteria, and examples for quality assessment are detailed in the Supplementary materials.

### Statistical analysis

All statistical analyses were performed using the R software (version 4.4.2) within the RStudio environment (version 2024.04.2–764). A detailed description of the statistical methods is provided in the Supplementary materials.

Analyses were conducted separately for the prevalence of *Helicobacter pylori* and for the associations between SDoH and *Helicobacter pylori* infection.

1. Prevalence Pooling: The pooled prevalence of *Helicobacter pylori* infection was calculated using a random-effects model. The Paule-Mandel estimator was used for τ^2^ (between-study variance), with the Hartung-Knapp adjustment applied. Prevalence proportions were transformed using the Freeman-Tukey double arcsine transformation prior to pooling and then back-transformed for presentation.
2. Association Pooling (SDoH → *Helicobacter pylori*): Crude odds ratios (cORs) were calculated from original 2 × 2 tables. The Mantel–Haenszel method under a random-effects model framework (incorporating Hartung-Knapp adjustment and Paule-Mandel τ^2^ estimator) was used for pooling. The Haldane-Anscombe continuity correction was applied to address zero cells.
3. Small-Study Effects / Publication Bias: Funnel plots were generated for visual inspection. For meta-analyses containing 10 or more studies (k ≥ 10), Egger’s linear regression test and Begg’s rank correlation test were performed as exploratory sensitivity analyses to assess potential small-study effects.
4. Subgroup Analysis: Pre-specified subgroup analyses were conducted by geographic region, age group, population type, and *Helicobacter pylori* diagnostic method. Quantitative synthesis within a subgroup was performed only when at least two studies were available (k ≥ 2).
5. Meta-Regression (Exploratory): Exploratory univariable meta-regression analyses were performed. Candidate covariates included publication year, region, age group, population characteristics, and diagnostic method. Detailed model specifications and formulas are provided in Section 3 of the Supplementary materials.

All hypothesis tests were two-sided, and a *p*-value of less than 0.05 was considered statistically significant.

## Results

### Study selection

**Figure 1:**
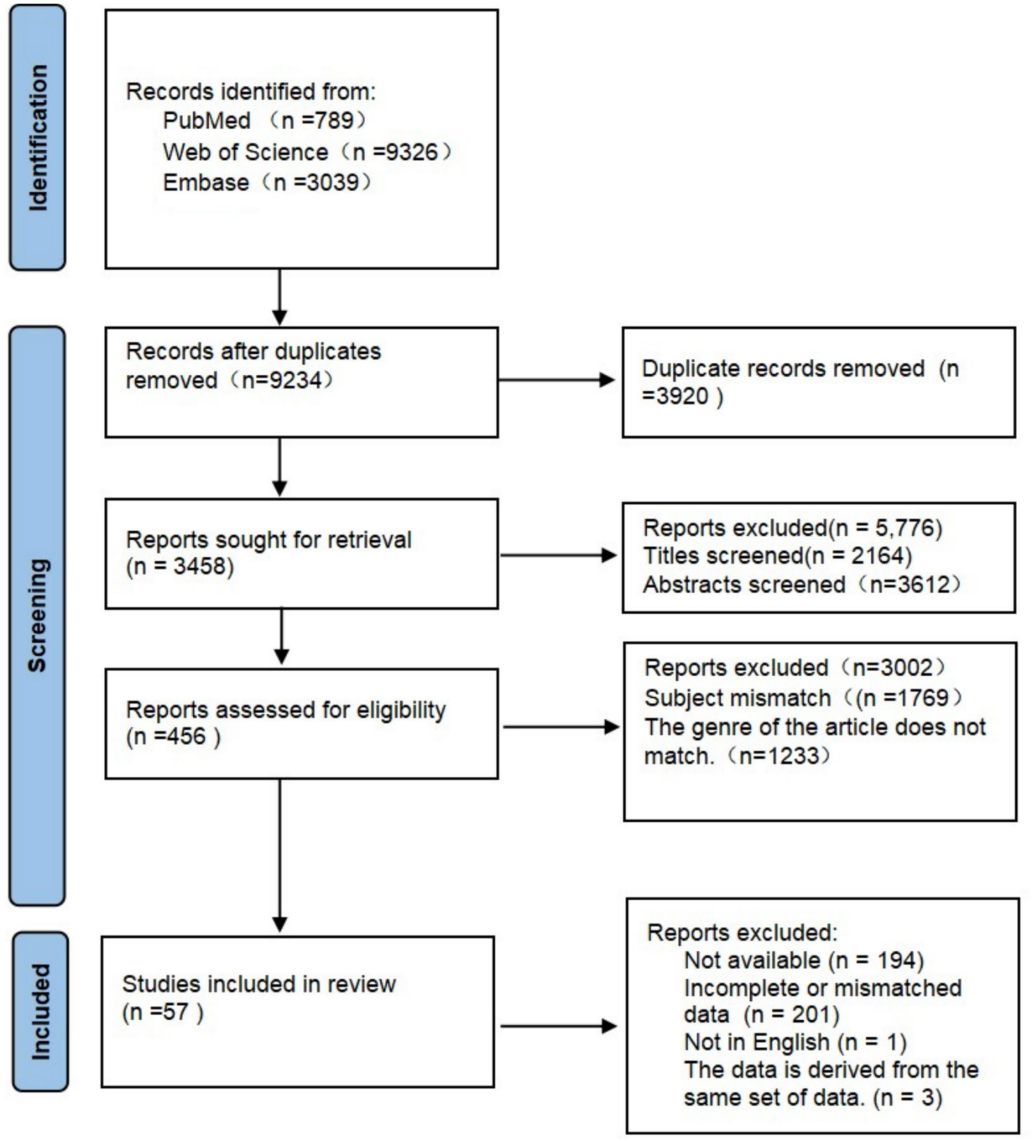
PRISMA flowchart. PRISMA 2020 flowchart summarizing the identification, screening, eligibility assessment, and final inclusion of studies. A total of 57 studies met the inclusion criteria and were included in the meta-analysis.

The study selection process is detailed in the PRISMA flow diagram (Figure 1). A total of 13,154 records were identified through database searching (PubMed: 789; Web of Science: 9,326; Embase: 3,039). After removing 3,920 duplicates, 9,234 records were screened based on titles and abstracts. Of these, 5,776 records were excluded as irrelevant. Full texts were sought for the remaining 3,458 records. A further 3,002 records were excluded during the full-text screening phase for reasons such as mismatched topic or ineligible publication type (e.g., reviews, commentaries). Consequently, 456 full-text articles were assessed for eligibility. Among these, 399 articles were excluded for the following reasons: full text unavailable (*n* = 194), key data missing or inconsistent (*n* = 201), non-English publication (*n* = 1), or duplicate dataset (*n* = 3). Finally, 57 studies were included in the systematic review and meta-analysis.

### Characteristics of included studies

A total of 57 studies were included (Table 1), published between 1993 and 2025. These studies covered Africa, Asia, Europe, and Latin America, with a higher proportion originating from East Asia (predominantly China). The study design was predominantly cross-sectional (approximately 91%). The cumulative sample size across all studies was 387,956 participants. Study populations were primarily general populations, with some studies focusing specifically on children, older adult(s), individuals, or those with gastrointestinal symptoms. The most frequently used diagnostic method was serology, followed by the UBT, stool antigen test, and mixed methods. Eight categories of SDoH were analyzed: household overcrowding, water safety, education, marital status, occupational status, residence type, socioeconomic status, and sanitation conditions.

**Table 1:** Characteristics of the study and patient demographics.

Author (Year)	Location	Population characteristics	Sample size	Methods for diagnosing *Helicobacter pylori*	The rate/prevalence of *Helicobacter pylori* infection	Include SDoH variables	Research design type	NOS score
Phoebe Aitila (2019)	Uganda	Africa, children, accompanied by digestive tract symptoms	304	Serum antibody test	0.243	Drinking water safety, Sanitary conditions, Degree of family overcrowding	Cross-sectional study	3/8
Hassan A. Al-Shamahy (2005)	Yemen	Middle East, Children	572	Serum antibody test	0.09	Social economic status, Degree of family overcrowding, Drinking water safety	Cross-sectional study	3/9
Dagninet Alelign (2023)	Ethiopia	Africa, adults, with digestive tract symptoms	422	Serum antibody test	0.32	Place of residence, Marital, Occupational status, Education, Degree of family overcrowding	Cross-sectional study	3/8
Andargachew Almaw (2024)	Ethiopia	Africa, adults, with digestive tract symptoms	384	Serum antibody test	0.12	Occupational status, Place of residence, Education, Degree of family overcrowding, Drinking water safety, Occupational status, Degree of family overcrowding, Sanitary conditions, Drinking water safety	Cross-sectional study	3/8
Alemayehu Sayih Belay (2020)	Ethiopia	Africa, adults, with digestive tract symptoms	208	Serum antibody test	0.43	Occupational status, Degree of family overcrowding, Sanitary conditions, Drinking water safety	Cross-sectional study	3/8
Jan Bureš (2012)	Czech Republic	Europe, including teenagers and above	1,427	Urea Breath Test (UBT)	0.24	Education, Marital	Cross-sectional study	4/9
María González-Pons (2017)	The United States	Latin America, adults, Hispanic	528	Serum antibody test	0.33	Marital	Cross-sectional study	4/9
Nikko Darnindro (2015)	Indonesia	East Asia, adults, with digestive tract symptoms	96	Serum antibody test	0.23	Drinking water safety	Cross-sectional study	3/8
Vitor Camilo Cavalcante Dattoli (2010)	Brazil	Latin America, children	1,104	Serum antibody test	0.29	Sanitary conditions	Prospective cohort study	9
C. H. Fall (1997)	Britain	Europe, older adult(s)	1,020	Serum antibody test	0.46	Social economic status, Degree of family overcrowding, Drinking water safety, Sanitary conditions	Retrospective cohort study	9
Ghislaine Florice Faujo Nintewoue (2023)	Cameroon	Africa, general population, with digestive tract symptoms	266	Rapid Urease Test (RUT)	0.72	Social economic status	Cross-sectional study	3/8
Robin B. Harris (2022)	The United States	Latin America, older adult(s), indigenous people	106	Urea Breath Test (UBT)	0.18	Drinking water safety	Cross-sectional study	4/9
Elin Hestvik (2011)	Norway	Europe, children	236	Serum antibody test	0.23	Degree of family overcrowding, Drinking water safety, Sanitary conditions, Social economic status	Cross-sectional study	3/8
Loi N. Ho (2024)	Vietnam	East Asia, older adult(s)	430	Rapid Urease Test (RUT)	0.56	Occupational status	Cross-sectional study	3/8
Thai Hoang Che (2022)	Vietnam	East Asia, teenagers	1,460	Fecal antigen test	0.88	Place of residence	Cross-sectional study	3/8
Seyedeh Amineh Hojati (2021)	Iran	Middle East, general population	233	Mixing	0.47	Education, Marital, Occupational status, Drinking water safety	Cross-sectional study	3/8
Jingjing Hu (2020)	China	East Asia, Children	1,355	Urea Breath Test (UBT)	0.23	Degree of family overcrowding	Cross-sectional study	3/8
Nagamitsu Iso (2005)	Japan	East Asia, general population	219	Mixing	0.65	Sanitary conditions	Cross-sectional study	3/8
Qi Jiang (2025)	China	East Asia, general population	4,361	Serum antibody test	0.71	Drinking water safety	Cross-sectional study	3/7
Ghalia Khoder (2019)	United Arab Emirates	Middle East, general population	350	Fecal antigen test	0.41	Occupational status, Degree of family overcrowding	Cross-sectional study	4/10
Mahdieh Khoshakhlagh (2024)	Iran	Middle East, youth, teenager	933	Serum antibody test	0.39	Marital, Education, Occupational status	Cross-sectional study	3/9
Zebasil Mnichil (2023)	Ethiopia	Africa, adults, with digestive tract symptoms	403	Serum antibody test	0.62	Place of residence, Marital, Social economic status	Cross-sectional study	3/9
Nilufer Ozaydin (2013)	Türkiye	Middle East, general population	4,622	Serum antibody test	0.83	Education, Drinking water safety	Cross-sectional study	3/9
Carolina Porras (2013)	Costa Rica	Latin America, general population	1852	Urea Breath Test (UBT)	0.79	Degree of family overcrowding, Education, Place of residence	Randomized controlled study	9
Duc Long Tran (2022)	Vietnam	East Asia, children, peptic ulcer	140	Mixing	0.46	Place of residence, Social economic status	Cross-sectional study	3/9
Van Tran (2022)	Ethiopia	Africa, Children	954	Serum antibody test	0.36	Place of residence, Degree of family overcrowding, Sanitary conditions	Cross-sectional study	3/9
Yan Xue (2019)	China	East Asia, general population, after *Helicobacter pylori* eradication	743	Mixing	0.05	Social economic status	Prospective cohort study	9
Hiwot Yisak (2022)	Ethiopia	Africa, pregnant woman	290	Fecal antigen test	0.18	Place of residence, Drinking water safety, Sanitary conditions	Cross-sectional study	3/9
Yangchun Zhu (2014)	China	East Asia, older adult(s)	5,417	Urea Breath Test (UBT)	0.63	Marital, Education, Degree of family overcrowding, Social economic status	Cross-sectional study	3/9
Hsin Chi (2009)	China	East Asia, teenagers	106	Urea Breath Test (UBT)	0.55	Social economic status	Cross-sectional study	3/9
Elin Hestvik (2010)	Uganda	Africa, children, health	427	Fecal antigen test	0.44	Degree of family overcrowding, Drinking water safety, Social economic status	Cross-sectional study	3/9
Chenyu Jiang (2025)	America	Latin America, general population	3,573	Serum antibody test	0.45	Education	Cross-sectional study	4/9
Paulius Jonaitis (2025)	Lithuania	Eastern Europe, adults	1,046	Serum antibody test	0.63	Education, Drinking water safety	Cross-sectional study	4/9
Danni Liu (2023)	China	East Asia, general population	1,355	Urea Breath Test (UBT)	0.46	Place of residence, Education, Marital	Cross-sectional study	3/9
D Palli (1993)	Italy	Europe, general population	930	Serum antibody test	0.45	Place of residence, Social economic status	Cross-sectional study	3/9
Idowu O. Senbanjo (2014)	Nigeria	Africa, children, health	118	Serum antibody test	0.64	Social economic status, Degree of family overcrowding, Drinking water safety	Cross-sectional study	3/9
Ari Fahrial Syam (2015)	Ari Fahrial Syam	East Asia, general population, with digestive tract symptoms	267	Mixing	0.22	Social economic status, Occupational status, Marital, Drinking water safety, Sanitary conditions	Cross-sectional study	3/9
Evariste Tshibangu-Kabamba (2021)	Congo	Africa, general population, with digestive tract symptoms	425	Serum antibody test	0.54	Occupational status, Degree of family overcrowding, Social economic status	Cross-sectional study	3/9
Yong Xie (2020)	China	East Asia, general population, after *Helicobacter pylori* eradication	3,728	Not reported	0.02	Education	Prospective cohort study	8
Thai Hoang Che (2023)	Vietnam	East Asia, Children	1,409	Fecal antigen test	0.88	Social economic status, Place of residence, Degree of family overcrowding	Cross-sectional study	3/8
Huan-Lin Chen (2014)	China	East Asia, general population	690	Urea Breath Test (UBT)	0.72	Marital	Cross-sectional study	3/8
Run-Xiang Chen (2023)	China	East Asia, general population	3,632	Urea Breath Test (UBT)	0.39	Occupational status, Education, Drinking water safety	Cross-sectional study	3/8
Irigrácin Lima Diniz Basílio (2018)	Brazil	Latin America, general population	200	Mixing	0.6	Education, Social economic status, Degree of family overcrowding, Drinking water safety	Cross-sectional study	3/9
Yasmine Samir Galal (2019)	Egypt	Africa, children, accompanied by digestive tract symptoms	630	Fecal antigen test	0.65	Place of residence	Cross-sectional study	4/9
Yasuyuki Goto (2016)	Indonesia	South Asia, general population, health	196	Urea Breath Test (UBT)	0.15	Education, Social economic status, Marital	Cross-sectional study	3/8
Shiwen He (2024)	China	East Asia, general population	23,254	Urea Breath Test (UBT)	0.26	Marital, Occupational status	Cross-sectional study	3/9
Hyasinta Jaka (2016)	Tanzania	Africa, general population, with digestive tract symptoms	202	Serum antibody test	0.39	Occupational status, Education, Drinking water safety, Place of residence	Cross-sectional study	3/8
Jeong Hoon Lee (2016)	South Korea	East Asia, general population	4,963	Serum antibody test	0.51	Social economic status, Education	Cross-sectional study	3/9
Seon Hee Lim (2018)	South Korea	East Asia, general population	16,885	Serum antibody test	0.44	Social economic status, Education,	Cross-sectional study	3/9
Seon Hee Lim (2013)	South Korea	East Asia, general population, no history of *Helicobacter pylori* infection	8,688	Serum antibody test	0.545	Socioeconomic status	Cross-sectional study	3/9
Laure Brigitte Kouitcheu Mabeku (2018)	Cameroon	Africa, adults, with digestive tract symptoms	205	Serum antibody test	0.64	Socioeconomic status, Degree of family overcrowding	Cross-sectional study	3/9
Suresh Mehata (2021)	Nepal	South Asia, Children	1,023	Fecal antigen test	0.18	Education, Place of residence, Social economic status	Cross-sectional study	3/9
Abdurahaman Seid (2018)	Ethiopia	Africa, general population, with digestive tract symptoms	189	Serum antibody test	0.61	Marital, Place of residence, Occupational status	Cross-sectional study	3/9
Hannah Spotts (2020)	Ethiopia	Africa, Children	434	Serum antibody test	0.65	Place of residence, Drinking water safety, Sanitary conditions, Degree of family overcrowding, Social economic status	Cross-sectional study	3/9
Fuhua Zhang (2021)	China	East Asia, general population	21,291	Urea Breath Test (UBT)	0.53	Education, Degree of family overcrowding, Marital, Occupational status	Cross-sectional study	3/9
Yan Zhou (2022)	China	East Asia, general population	3,704	Urea Breath Test (UBT)	0.44	Occupational status, Place of residence, Education, Degree of family overcrowding, Social economic status	Cross-sectional study	3/9
Yan Zhou (2017)	China	East Asia, general population	4,780	Urea Breath Test (UBT)	0.60	Occupational status, Place of residence, Education, Degree of family overcrowding, Social economic status	Cross-sectional study	3/9

The NOS column of the cross-sectional study is represented by X/Y: the first item is NOS-xs2 (0–4, prevalence/descriptive cross-sectional), and the second item is NOS-xs (0–9, association/analytical cross-sectional). The inclusion criteria for this study are: NOS-xs2 ≥ 3 and NOS-xs ≥ 6. These two values come from different scales and cannot be added or directly compared.

### Quality assessment results

The quality of cross-sectional studies was assessed using the NOS-xs scale (0–9 points, for association analysis) and the NOS-xs2 scale (0–4 points, for prevalence analysis). Cohort or randomized studies were assessed using the standard Newcastle-Ottawa Scale (NOS, 0–9 points). As there is no universal cut-off for the NOS scales, pre-defined thresholds were applied: a score of NOS-xs ≥ 8 or NOS ≥ 8 was considered high quality for association analysis; a score of NOS-xs2 ≥ 4 was considered high quality for prevalence analysis; scores of 3 (for NOS-xs2) or 6–7 (for NOS-xs/NOS) were considered moderate quality.

Most studies met the pre-defined quality thresholds (NOS/NOS-xs ≥ 6; NOS-xs2 ≥ 3). For the prevalence analysis, 12 studies were rated as high quality and 45 as moderate quality. For the SDoH-*Helicobacter pylori* association analysis, 56 studies were rated as high quality and one as moderate quality (Qi Jiang 2025, NOS-xs = 7). Common limitations reported included insufficient sample representativeness and non-response bias.

### Prevalence of *Helicobacter pylori* infection

**Figure 2:**
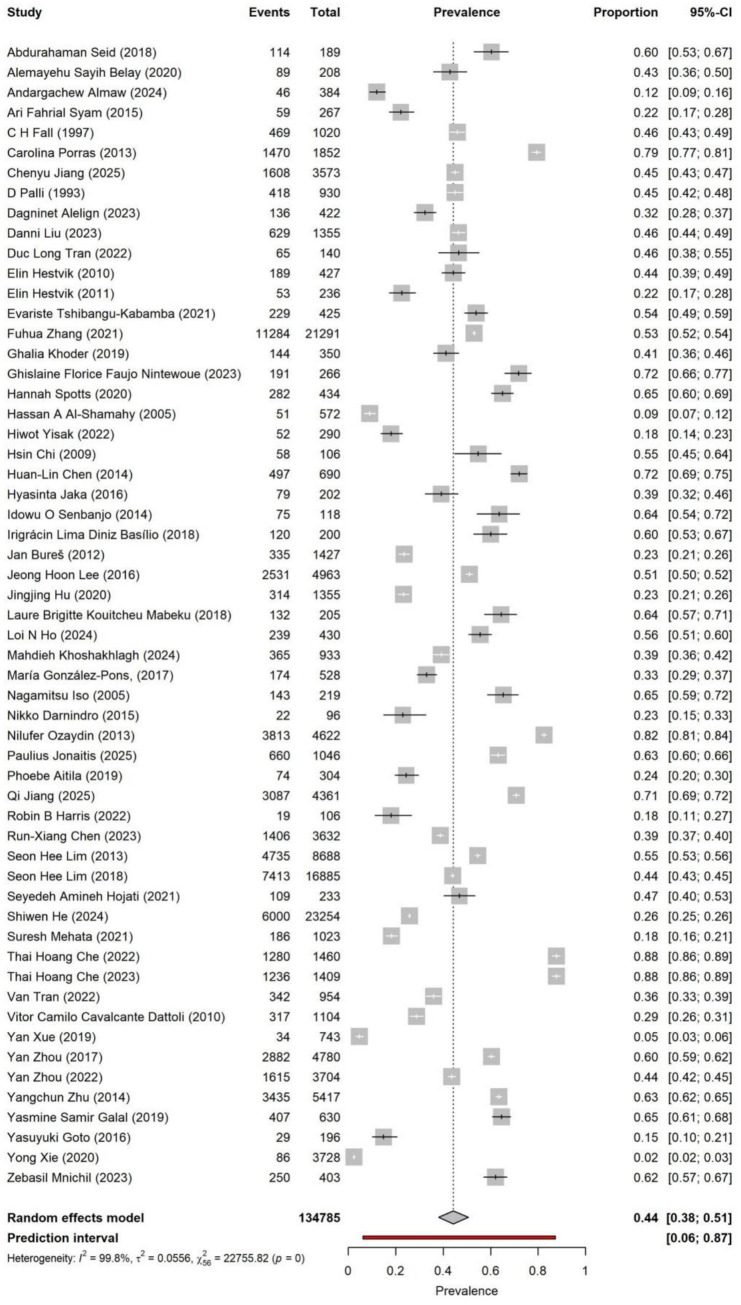
Meta-analysis of the overall infection rate. Pooled prevalence was 44% (95% CI: 38–51%), with substantial heterogeneity across studies (*I*^2^ = 99.8%).

The overall pooled prevalence of *Helicobacter pylori* infection, derived from a random-effects model, was 43% (95% CI: 36–50%). The prediction interval was 0.08–0.88, indicating substantial variation in prevalence estimates across different settings. Heterogeneity was extremely high (*I*^2^ = 99.6%, *Q*-test *p* < 0.0001). Consequently, pre-specified subgroup analyses by region, age, population characteristics, and diagnostic method were conducted, and meta-regression was performed to explore potential sources of heterogeneity (forest plot in Figure 2).

**Figure 3:**
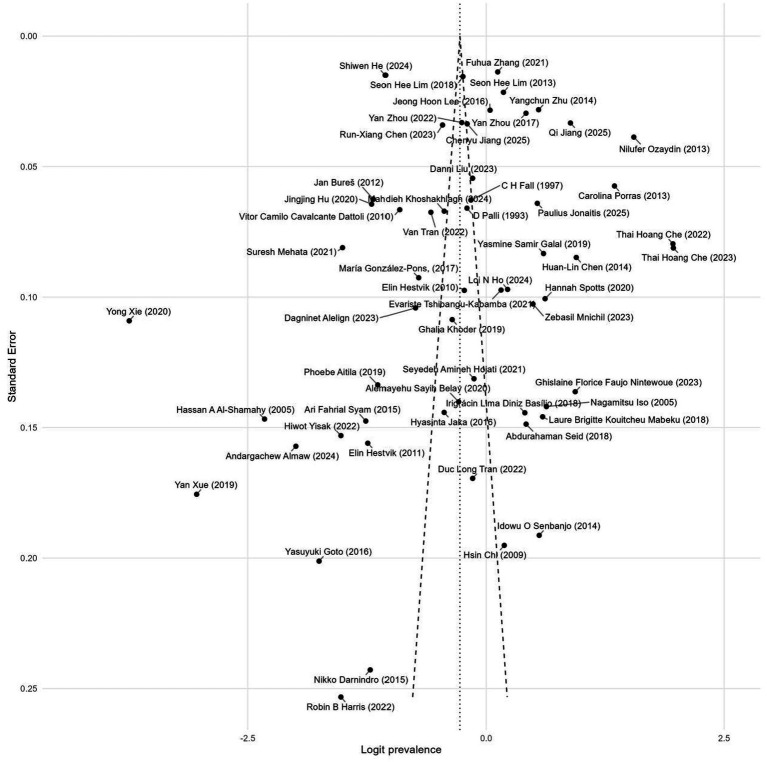
Funnel chart of infection rate. Visual inspection showed no major asymmetry, consistent with non-significant Egger’s and Peters’ tests.

On the logit-transformed prevalence scale, the funnel plot (Figure 3) appeared approximately symmetrical. Egger’s regression test showed an intercept of −0.107 (SE 0.139; *t* = −0.03; *p* = 0.973), and Peters’ 1/*n* test was also non-significant (*z* = −0.63; *p* = 0.530), suggesting no clear small-study effect. Begg’s rank correlation test indicated mild asymmetry (*z* = −2.00; *p* = 0.046). Considering the results of all three tests and the visual inspection of the funnel plot, there was no strong evidence for systematic publication bias.

Subgroup analyses by region, age, symptom status, and diagnostic method revealed that only the diagnostic method contributed to statistically significant between-subgroup differences (Supplementary Figures 1–4). Meta-regression results indicated that the pre-specified covariates explained only a low proportion of the observed heterogeneity, suggesting the presence of other important, unmeasured sources. Detailed results of the subgroup analyses and meta-regression are provided in the Supplementary materials.

### Association between social determinants of health (SDoH) and *Helicobacter pylori* infection

#### Results of association analysis

**Figure 4:**
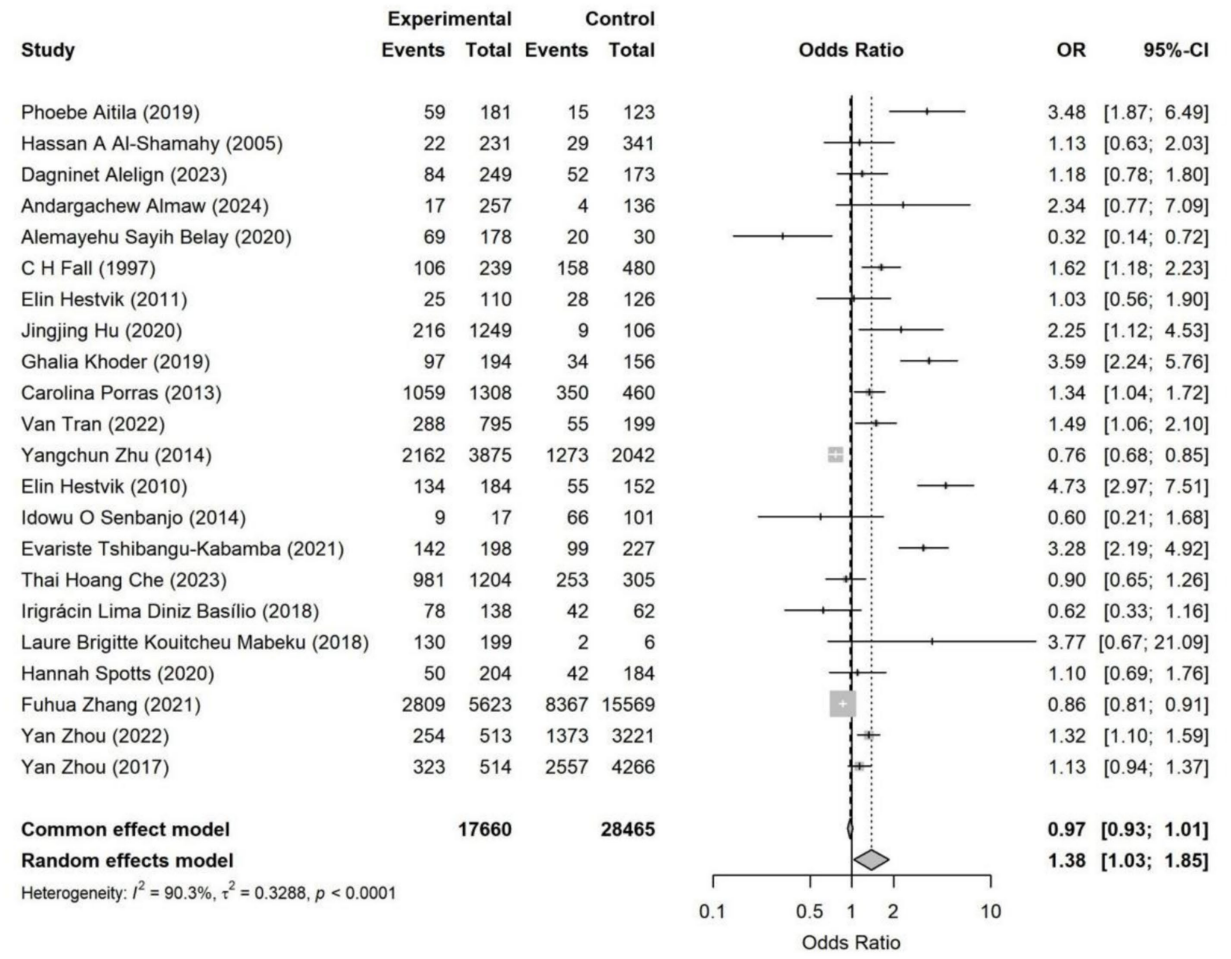
Forest plot of household overcrowding—*Helicobacter pylori* infection. Pooled analysis showed that household overcrowding was significantly associated with higher *Helicobacter pylori* infection risk (OR = 1.38, 95% CI: 1.03–1.85). Substantial heterogeneity was observed across studies (*I*^2^ = 90.3%). Results were synthesized using a random-effects model.

**Figure 5:**
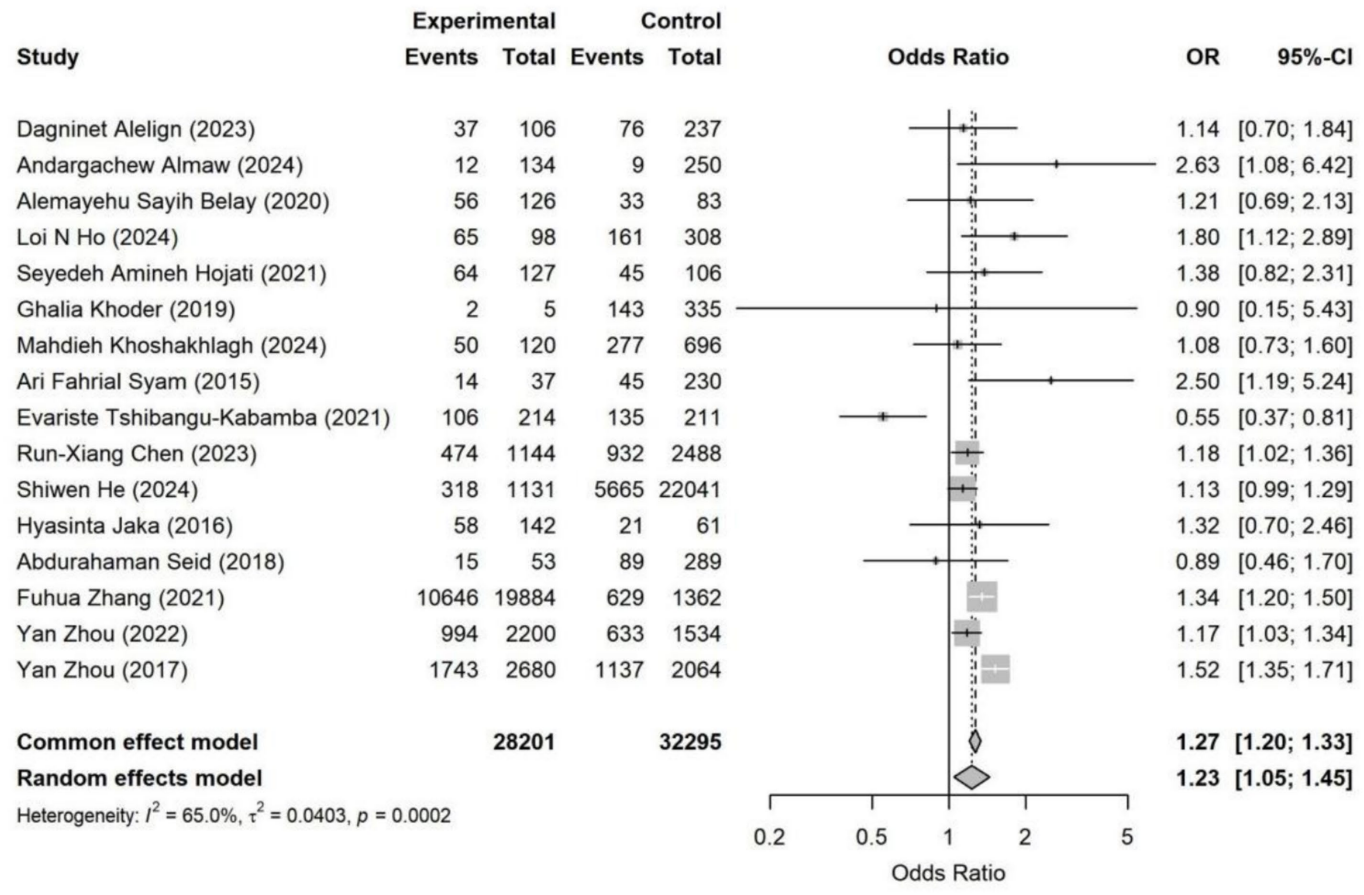
Forest plot of occupational status—*Helicobacter pylori* infection. The random-effects model showed that unstable or disadvantaged occupational status was significantly associated with a higher risk of *Helicobacter pylori* infection (OR = 1.23, 95% CI: 1.05–1.45). Moderate heterogeneity was observed (*I*^2^ = 65.0%).

**Figure 6:**
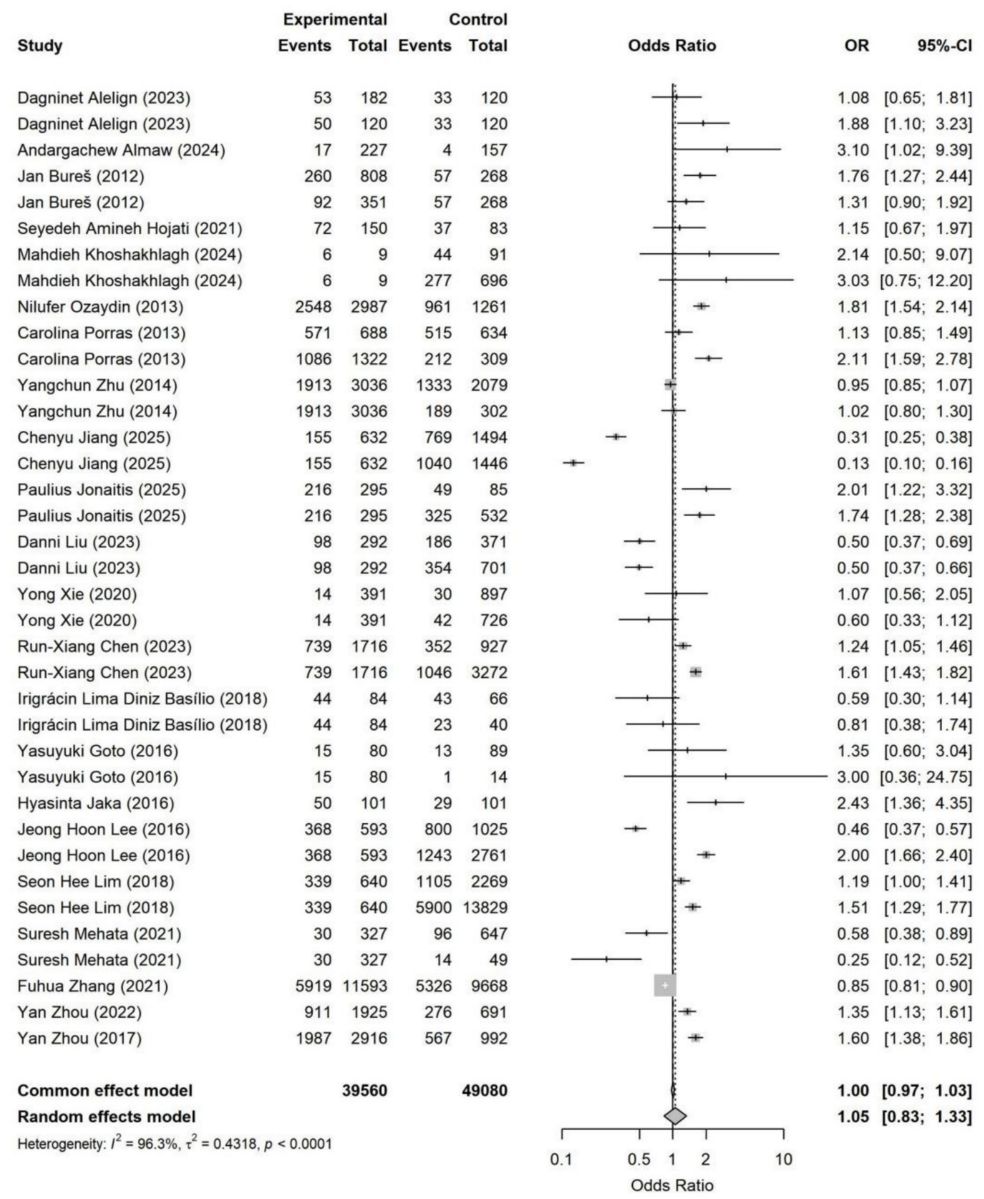
Forest plot of educational status—*Helicobacter pylori* infection. Lower educational attainment was not significantly associated with *Helicobacter pylori* infection in the random-effects model (OR = 1.05, 95% CI: 0.83–1.33), with substantial between-study heterogeneity (*I*^2^ = 96.3%). Effect estimates varied widely across settings, and no consistent direction was observed.

**Figure 7:**
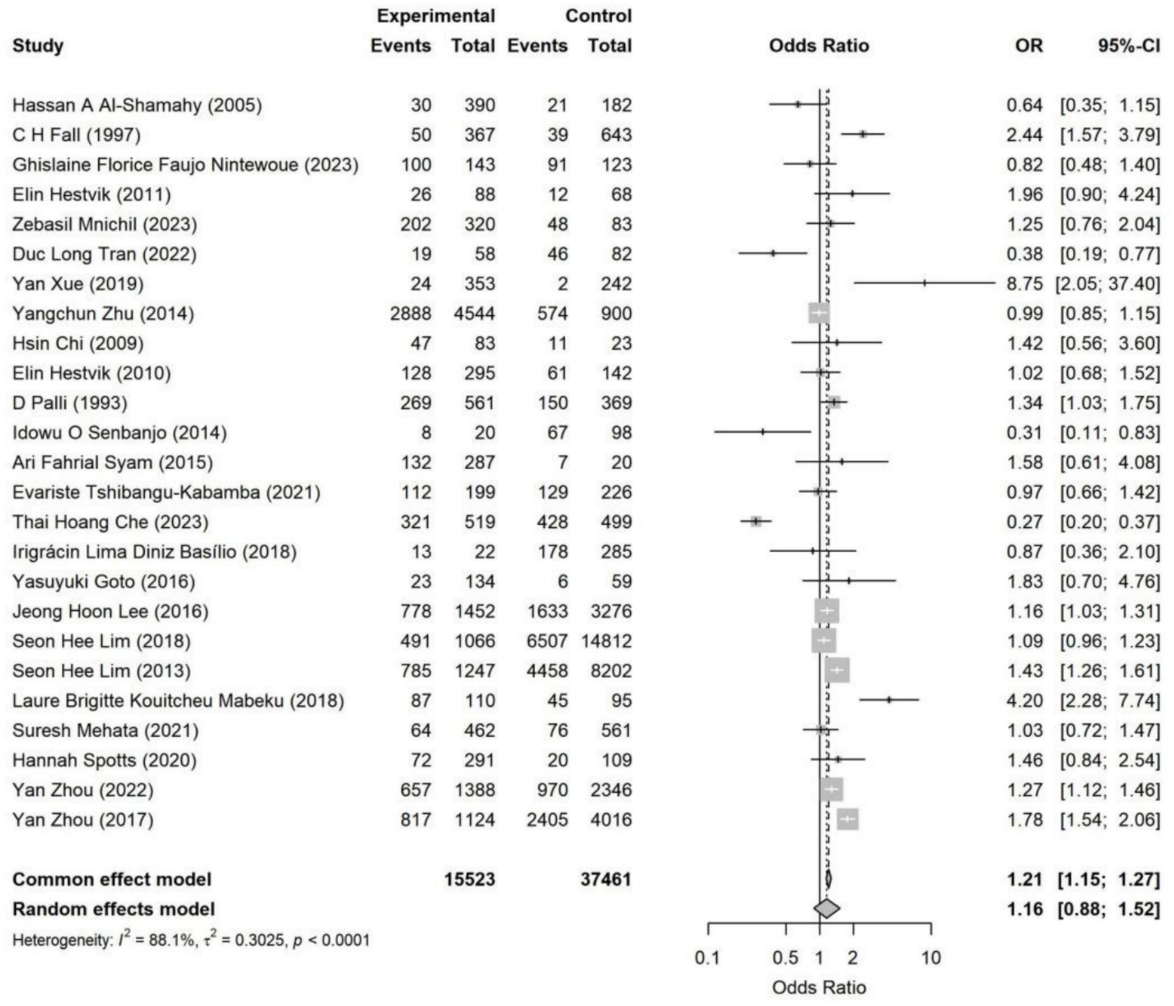
Forest plot of socioeconomic status—*Helicobacter pylori* infection. The combined results showed that individuals with a lower socioeconomic status had a slightly increased risk of *Helicobacter pylori* infection (random effects model OR = 1.16, 95% CI: 0.88–1.52), but the difference was not statistically significant. The heterogeneity among the studies was moderate to high (*I*^2^ = 88.1%), suggesting that there were significant differences in the definition of SES and social background among the studies.

**Figure 8:**
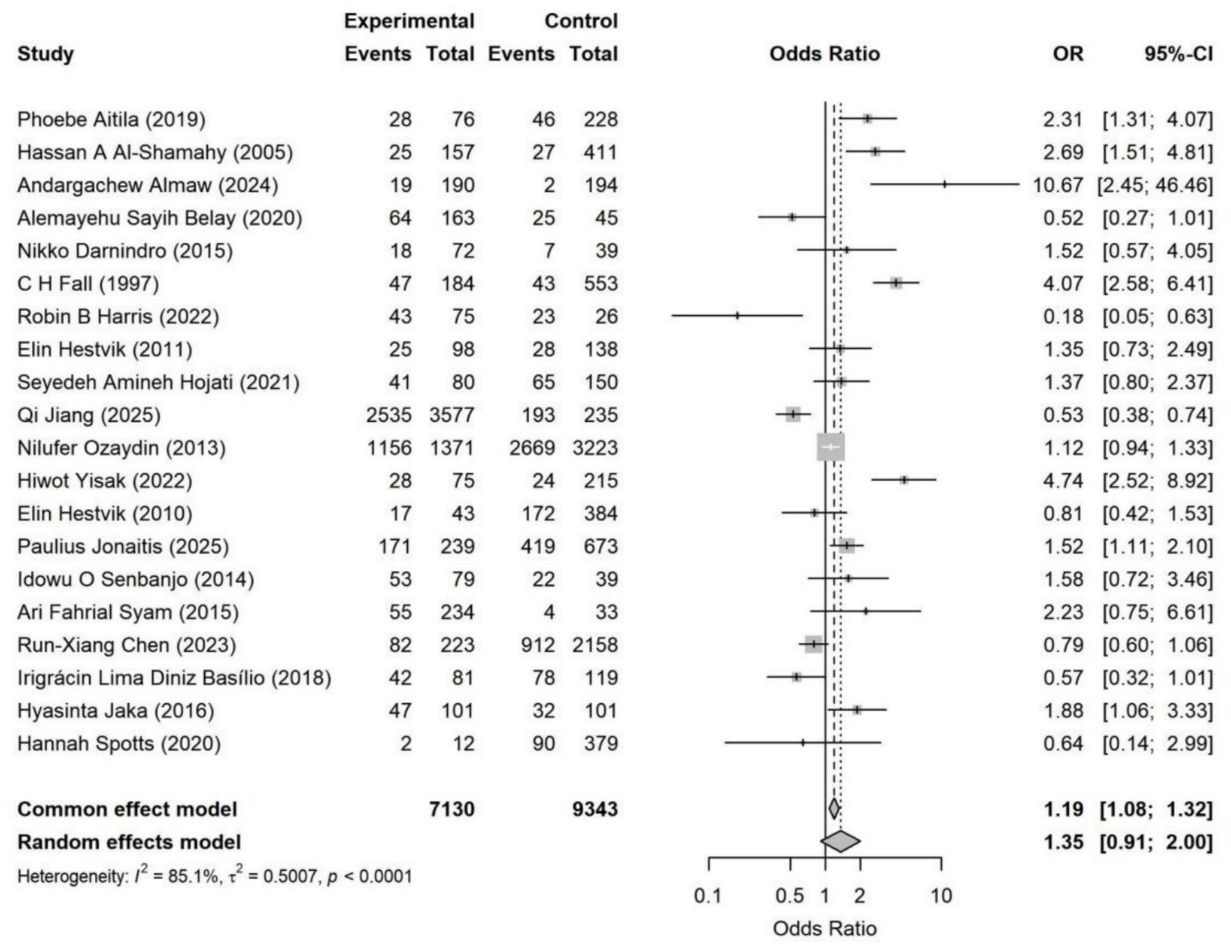
Forest plot of drinking water sources—*Helicobacter pylori* infection. Poor drinking water sources (unimproved water sources) were associated with an overall increased risk of *Helicobacter pylori* infection (random effects model OR = 1.35, 95% CI: 0.91–2.00). The effect direction was consistent in most studies, but the effect sizes varied significantly, and there was substantial heterogeneity among the studies (*I*^2^ = 85.1%). The overall association was not statistically significant.

**Figure 9:**
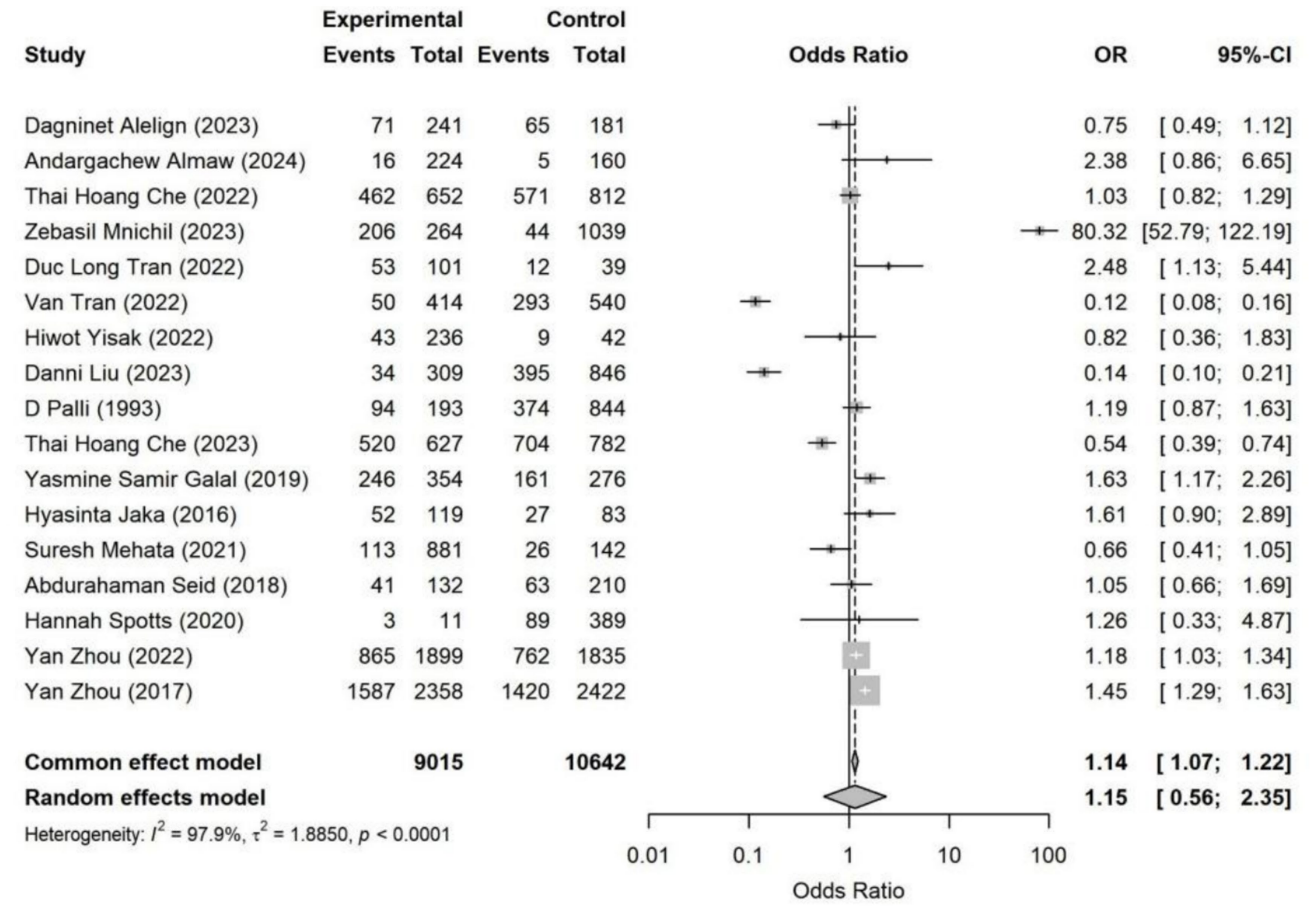
Housing situation—Forest plot of *Helicobacter pylori* infection. Pooled analysis shows that unfavorable housing conditions are modestly associated with a higher risk of *Helicobacter pylori* infection (random-effects OR = 1.15, 95% CI: 0.56–2.35). Considerable between-study heterogeneity was observed (*I*^2^ = 97.9%), indicating substantial contextual variation across settings.

**Figure 10:**
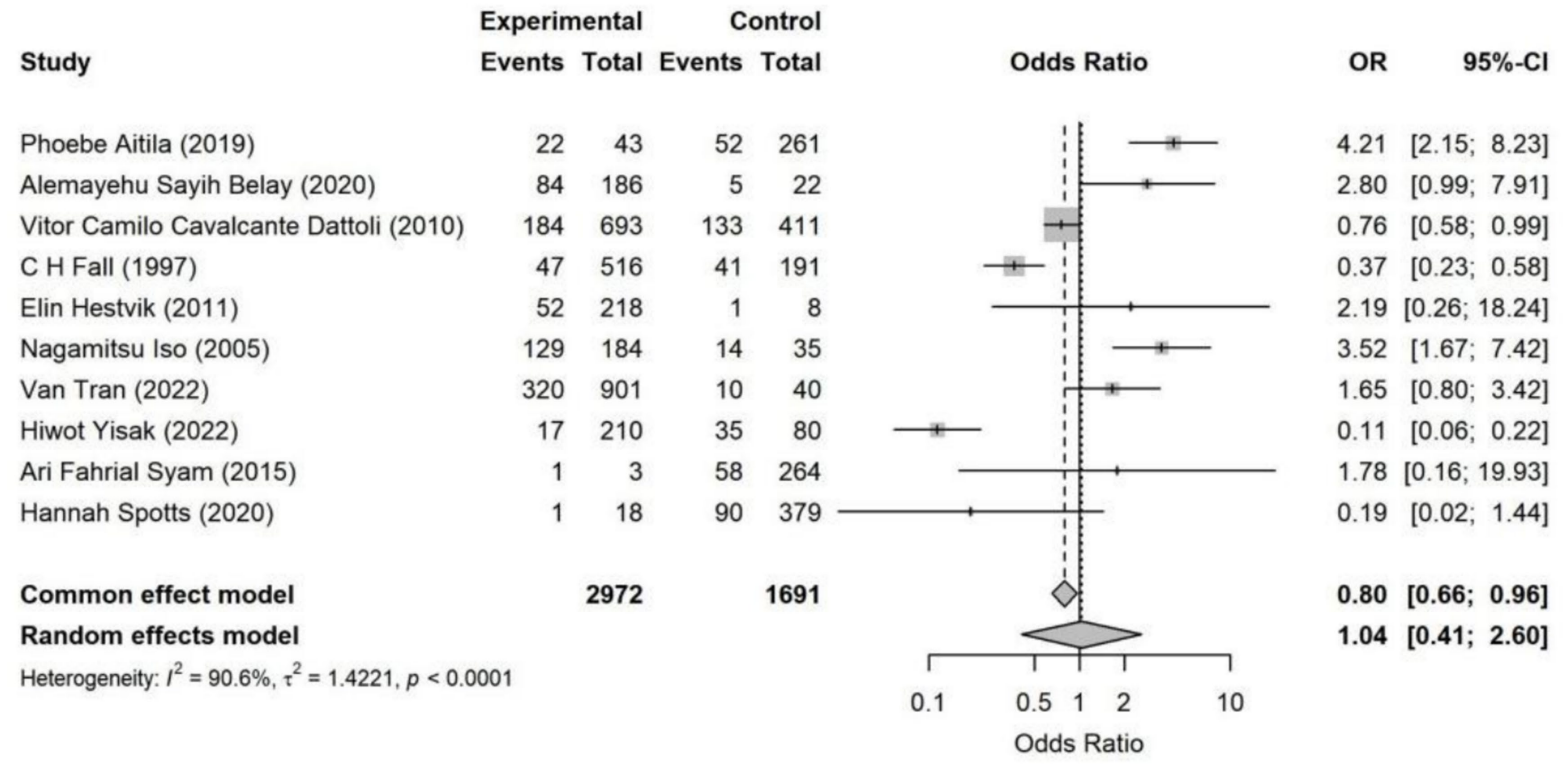
Forest plot of hygiene conditions—*Helicobacter pylori* infection. The overall effect indicated that there was no significant association between hygiene conditions and *Helicobacter pylori* infection (OR = 1.04, 95% CI: 0.41–2.60). The effect directions across studies were inconsistent, and heterogeneity was high (*I*^2^ = 90.6%).

**Figure 11:**
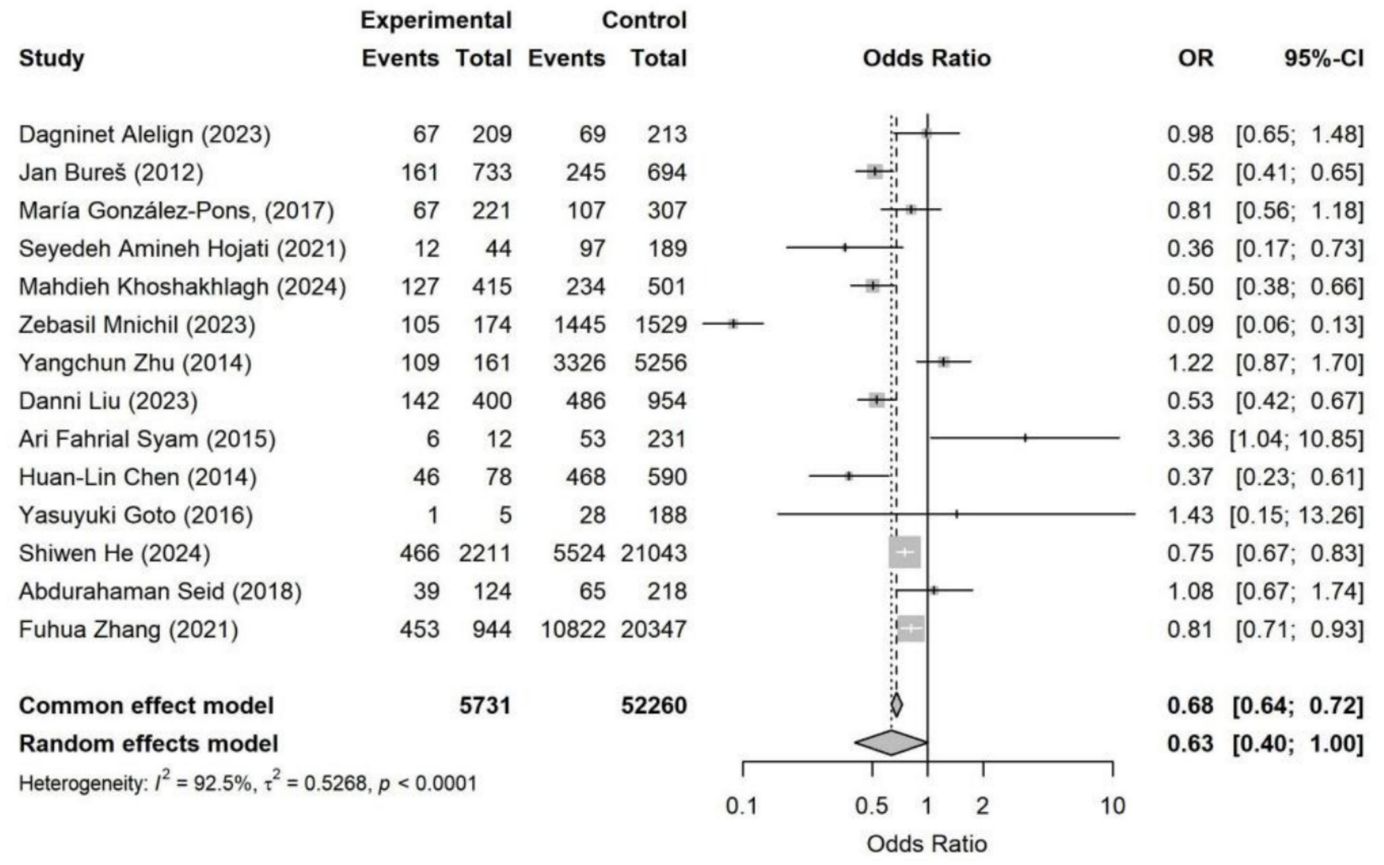
Forest plot of marital status—*Helicobacter pylori* infection. Across 15 studies, marital status showed no significant association with *Helicobacter pylori* infection (random-effects OR = 0.63, 95% CI: 0.40–1.00). Substantial heterogeneity was observed (*I*^2^ = 92.5%).

The overall pooled analysis indicated that most adverse SDoH were associated with a directionally consistent increase in the risk of *Helicobacter pylori* infection (OR > 1). These factors included household overcrowding, unsafe drinking water conditions, low socioeconomic status, occupational instability, and low educational attainment. In contrast, an adverse marital status (being unmarried, divorced, widowed, or living alone) overall showed a directionally protective association (OR < 1). Specifically, household overcrowding (OR = 1.38, 95% CI: 1.03–1.85) and occupational instability (OR = 1.23, 95% CI: 1.05–1.45) were identified as statistically significant risk factors (Figures 4–11). The remaining SDoH (education, socioeconomic status, water source, living conditions, and sanitation) showed a consistent risk-increasing direction but did not reach statistical significance. Marital status exhibited a borderline protective effect (OR = 0.63, 95% CI: 0.40–1.00). All pooled estimates were accompanied by high heterogeneity (*I*^2^ mostly ranging from 80 to 95%).

**Figure 12:**
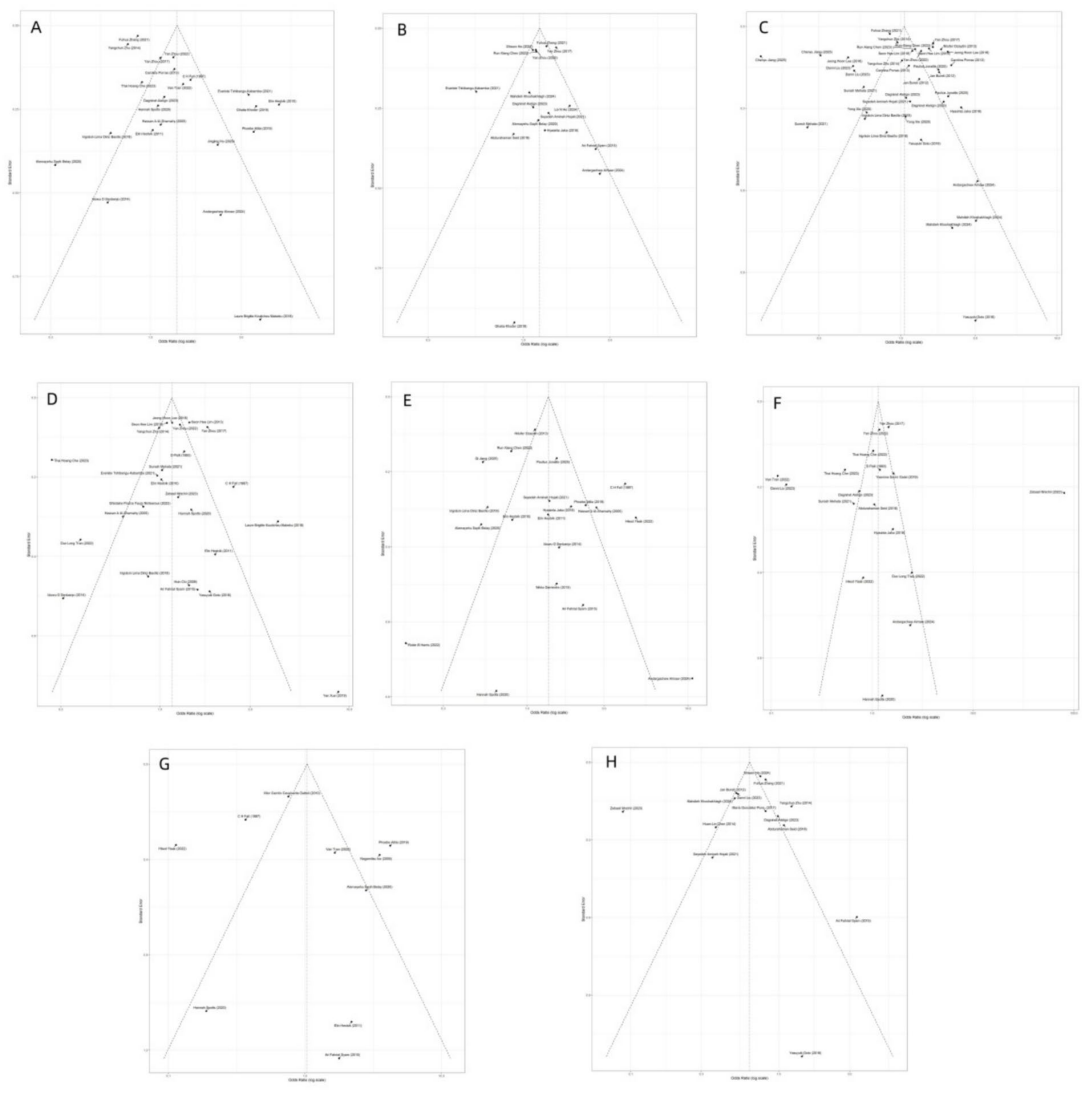
Funnel plots for publication bias across eight social determinants of health (SDoH) domains. **(A–H)** display funnel plots for: **(A)** household overcrowding, **(B)** occupational status, **(C)** educational level, **(D)** socioeconomic status, **(E)** drinking water source, **(F)** housing environment, **(G)** hygiene conditions, and **(H)** marital status. Each plot shows the distribution of study-specific effect sizes against their standard errors to visually assess small-study effects. Overall, no strong asymmetry was observed, except for household overcrowding, which showed mild asymmetry consistent with the Egger’s test results.

Funnel plots for each SDoH are presented in Figure 12. Overall, the stratified funnel plots appeared approximately symmetrical. The results of the Egger’s and Begg’s tests are detailed in Table 2. The analysis revealed significant asymmetry only for the stratum of household overcrowding: Egger’s intercept = 2.54, *t* = 3.22, *p* = 0.004, suggesting the possible presence of a small-study effect (where smaller studies are more likely to report higher risk estimates, OR > 1). However, Begg’s test did not reach significance (*p* = 0.672). No significant publication bias was detected in the other strata.

**Table 2:** Publication bias tests for SDoH–*Helicobacter pylori* associations.

SDoH	k	Egger_intercept	Egger_t	Egger_p	Begg_tau	Begg_p	Note
Degree of family overcrowding	22	2.537	3.22	0.00425	−15	0.672	
Drinking water safety	20	1.028	0.93	0.36500	6	0.846	
Education	37	0.537	0.40	0.68800	−78	0.308	
Marital	14	−1.112	−0.65	0.52900	−1	0.956	
Occupational status	16	−0.216	−0.31	0.76100	22	0.322	
Place of residence	17	−0.678	−0.23	0.82300	8	0.742	
Sanitary conditions	10	1.200	0.63	0.54600	3	0.788	
Social economic status	25	−0.421	−0.45	0.66000	8	0.852	

A trim-and-fill analysis was performed to explore potential bias specifically for the factor of household overcrowding. The Duval & Tweedie trim-and-fill method inferred no missing studies (k₀ = 0), and the pooled effect size remained unchanged after adjustment (OR = 1.38, 95% CI: 1.06–1.80). It is important to note that in the presence of high heterogeneity and a limited number of studies, the regression-based Egger’s test, due to its greater statistical power, is generally more sensitive to small-study effects than the rank correlation-based Begg’s test. This difference provides a reasonable explanation for the observed discrepancy between the two test results. Furthermore, the trim-and-fill analysis, which inferred no missing studies, suggests that the impact of such potential bias on the pooled effect estimate may be limited.

Exploratory subgroup analyses and meta-regression indicated that the association between SDoH and *Helicobacter pylori* infection exhibited notable geographic dependence. In contrast, age, the presence of gastrointestinal symptoms, and diagnostic methods showed limited moderating effects on the overall association. The overall proportion of heterogeneity explained by the examined covariates was low, suggesting the existence of important unmeasured contextual factors. Detailed results of all subgroup analyses and meta-regression are provided in the Supplementary materials.

#### Summary of SDoH domains with the strongest association to *Helicobacter pylori* infection

Overall, adverse living conditions and socioeconomic vulnerability demonstrated the strongest associations with an increased risk of infection. Household overcrowding (OR = 1.38) and occupational instability (OR = 1.23) represented stable and directionally consistent risk factors, with elevated risk observed across multiple subgroups. These factors reflect differences in living density, sanitation, and exposure environments, serving as crucial determinants for *Helicobacter pylori* transmission.

Although educational attainment was not statistically significant in the primary analysis, it exhibited a clear gradient effect: the risk was highest for low versus high education, followed by low versus medium education. This suggests that education may indirectly influence infection risk through pathways such as hygiene practices, health literacy, and access to healthcare services.

Socioeconomic status, water source, sanitation, and residence location all showed an overall direction toward increased risk. However, the estimates varied considerably across studies, potentially influenced by regional development levels, diagnostic methods, and population structure. The association with marital status was inconsistent in direction and weak in magnitude.

In summary, SDoH related to living density/environment appear to exert the greatest influence on infection risk, followed by factors indicating occupational and socioeconomic vulnerability. Educational attainment demonstrates a social gradient effect, representing another potentially critical domain.

### Subgroup analysis by educational attainment

When educational attainment was stratified into comparisons such as “low versus high” and “low versus medium,” the pooled ORs were consistently greater than 1. This indicates a higher risk of *Helicobacter pylori* infection associated with lower educational levels.

The effect size for the low- versus high-education comparison (OR = 3.49, 95% CI: 2.98–4.08; Supplementary Figure 31) was significantly stronger than that for the low- versus medium-education comparison (OR = 1.60, 95% CI: 1.38–1.86; Supplementary Figure 32), demonstrating a clear gradient relationship.

Both comparisons exhibited high heterogeneity (*I*^2^ ≈ 95–98%). While the direction of association was consistent across studies, the strength of the association was substantially influenced by geographic region and study design.

## Discussion

### Innovations of this study

*Helicobacter pylori* continues to pose a significant global public health burden, characterized by notable geographical disparities and population inequalities. This highlights the need to understand and manage the infection through the lens of SDoH (18, 19). Moreover, while effective treatment is fundamental for eradication, the primary challenge to achieving a rapid decline in infection rates at the population level—particularly in resource-limited settings—is the efficient identification of infected individuals (20, 21). Addressing this need, our study performed a systematic review and meta-analysis on the prevalence of *Helicobacter pylori* and the associations between multidimensional SDoH and *H. pylori* infection. We evaluated heterogeneity, publication bias, and result robustness, with stratified analyses by region, age group, and diagnostic method. This study provides a practical approach to the critical challenge of efficient case identification. Factors such as household crowding, low educational attainment, and occupational instability emerged as strong and readily accessible predictors for identifying communities and subpopulations at high risk of infection.

Additionally, this study presents several key innovations:

First, it incorporates studies from Africa, Asia, Europe, and Latin America, encompassing diverse age groups and population characteristics (e.g., children, older adult(s), and individuals with gastrointestinal symptoms). This globally representative and heterogeneous sample enhances the external validity of our findings, providing a more comprehensive reflection of the risk factors for *Helicobacter pylori* infection across different regions and social contexts. Compared to most previous studies focusing on single regions or homogeneous populations, our study offers more generalizable data to support global public health strategies and interventions.

Second, through the application of random-effects models, subgroup analyses, and meta-regression, this study provides an in-depth investigation into the sources of heterogeneity in *Helicobacter pylori* infection research. While prior studies have explored risk factors for *H. pylori*, few have systematically assessed heterogeneity to this extent. We identified that significant between-study differences may originate from various factors, including region, age, and diagnostic methods. This suggests that future research should further explore potential unmeasured contextual factors to more precisely identify high-risk groups for infection.

Third, this study not only considers multiple SDoH—such as household crowding, water quality, education level, and occupational instability—but also quantifies and compares their associations with *Helicobacter pylori* infection. Our findings reveal that household crowding (OR = 1.38) and occupational instability (OR = 1.23) are significant risk factors, indicating that these socio-environmental factors may substantially increase the risk of *H. pylori* infection by influencing living density and exposure settings. While previous research has often focused on single SDoH factors, our integrated analysis of multiple factors confirms significant socioeconomic inequalities in infection risk. This provides crucial scientific evidence for shifting from untargeted screening to precision public health interventions based on risk stratification, particularly in resource-limited settings.

Fourth, although education level was not a significant risk factor in the overall analysis, this study identified a clear educational gradient: low educational attainment was significantly associated with a higher risk of *Helicobacter pylori* infection than higher education groups. This finding offers a novel perspective on the potential role of education in shaping health behaviors, health literacy, and access to services. The disparity in infection risk across educational backgrounds suggests that public health interventions need to pay particular attention to groups with lower education levels.

Fifth, through subgroup analysis and meta-regression, this study further reveals the geographical dependency of the relationship between SDoH and *Helicobacter pylori* infection risk. This implies that factors, such as regional development levels, cultural contexts, and health policies, may influence the strength and direction of this association. Consequently, in practice, screening efforts should prioritize communities and individuals characterized by household crowding, occupational instability, and low educational attainment to enable precise targeting of resources. Screening strategies must incorporate the geographical variability patterns revealed in this study, tailoring intervention priorities to different regions. Operationally, promoting the standardization of diagnostic methods is essential to ensure comparability of risk surveillance and program evaluation data. It is important to emphasize that this approach does not replace treatment; rather, it optimizes the case-finding process (e.g., by focusing attention on specific high-risk groups) so that treatment resources can be allocated more accurately and efficiently to those most in need. This will ultimately accelerate progress toward the overarching goal of reducing the total burden of *Helicobacter pylori*-related diseases.

### Global prevalence differences and diagnostic method-driven heterogeneity

The results of this study indicate that the prevalence of *Helicobacter pylori* is 43%, with a wide predictive range (0.08–0.88), suggesting significant differences across regions, study populations, and diagnostic methods. It is noteworthy that our findings did not reveal a statistically significant difference in prevalence across continents (Africa, Asia, Europe, and Latin America), nor did we detect a clear long-term declining trend based on publication year. These unobserved results may suggest that, at a macro level, the social and environmental driving factors leading to high infection rates (e.g., household transmission) share commonalities across regions. Additionally, the progress in infection control globally may not be uniform, with improvements in some regions being offset by stagnation in others. Among all factors, differences in diagnostic methods were the most significant and consistent contributors to the variation in prevalence estimates. Our analysis showed that the highest prevalence was observed in studies using stool antigen testing (52%), followed by serology (49%), urease breath test (46%), and mixed methods (39%). This gradient clearly highlights that the diagnostic method itself is a key source of overestimation or bias in prevalence estimates. In conclusion, the pooled estimate (43%) of this study has limited external validity when applied to any specific region or population. Future research and prevention practices should go beyond focusing on a single overall prevalence rate and instead prioritize: (1) promoting the standardization of diagnostic methods in monitoring and research to enhance the comparability of evidence and (2) further identifying local, specific social determinants to develop more targeted, precision prevention strategies.

### Systematic bias in various diagnostic methods and their impact on prevalence estimation

Studies on the differences in diagnostic methods show that serology tends to overestimate the prevalence. It detects specific antibodies such as IgG/IgA, which may persist for months to years after eradication therapy. In some individuals, these antibodies can remain positive for an extended period, making it difficult to distinguish between past and current infection (22). Additionally, serological assays are often based on antigens from specific regional strains, and when used across regions with significant antigen heterogeneity, false positives may occur, further amplifying the bias in prevalence estimates (23). Furthermore, the urease breath test is considered one of the gold standards for detecting *H. pylori* active infection, but it is not entirely free of bias. Studies have found that the sensitivity of the urease breath test is lower in children due to insufficient gastric acid secretion and reduced urease activity (24). Similarly, the accuracy of the urease breath test may be compromised in patients with gastric ulcers, pyloric obstruction, or those on long-term proton pump inhibitor (PPI) therapy (25).

Stool antigen testing can detect current infection, but its sensitivity is significantly influenced by reagent quality and antigen preservation conditions. If stool samples are not properly stored under cold chain conditions, antigen proteins may degrade, leading to an increased false-negative rate (26). Additionally, some rapid test kits may have insufficient sensitivity to regional strains, potentially underestimating the true prevalence (27).

Finally, RUTs are highly dependent on sample collection methods. The sensitivity of RUTs is significantly influenced by the distribution of gastric mucosal inflammation, the amount of sample collected, and the presence of gastric atrophy (28). Therefore, in patients with patchy distribution or atrophic gastritis, the RUT is more likely to produce false negatives.

In summary, the differences in the sensitivity and specificity of various diagnostic methods, reagent sources, regional antigen heterogeneity, sample preservation conditions, and inherent factors of the subjects may introduce systematic biases, leading to significant discrepancies in prevalence estimates, both in this study and in the existing literature. This also explains the notable diagnostic method stratification results observed in our subgroup analysis, emphasizing the need to fully consider the impact of diagnostic methods in cross-study comparisons and policy formulation.

Moreover, these differences are not solely attributable to the diagnostic methods themselves, but they are closely related to local resources, costs, and availability. In high-resource settings, there may be a tendency to use more accurate diagnostic methods, whereas in low-resource settings, the choice of diagnostic method is more influenced by cost and accessibility, leading to the use of cheaper, more readily available methods despite their significant limitations (6, 29). This further explains the significant diagnostic method stratification results observed in our subgroup analysis, highlighting the need to balance diagnostic accuracy and cost-effectiveness, especially in resource-limited areas.

Future efforts to address the limitations of *H. pylori* detection should focus on improving diagnostic accuracy, optimizing method combinations, and reducing costs. First, enhancing the sensitivity and specificity of existing diagnostic methods, such as developing antigens adapted to regional strains and improving the quality of rapid urease and stool antigen tests, is crucial. Second, exploring new non-invasive diagnostic methods, such as molecular diagnostics and novel biomarkers, could provide higher sensitivity. Furthermore, the combined use of multiple methods and intelligent diagnostic platforms could improve diagnostic accuracy and efficiency. Additionally, improving the accessibility of diagnostic tools, particularly in low-resource regions, by promoting low-cost and stable testing tools and strengthening the training of primary healthcare workers, is essential. Global cooperation and cross-country data sharing will also help to better understand the infection characteristics in different regions and develop more effective diagnostic strategies. By combining technological innovation with policy support, early screening for *H. pylori* can be made more efficient, reducing misdiagnosis and missed diagnoses, ultimately improving global public health.

### Assessment of publication bias and the limited explanatory power of macro-level covariates regarding heterogeneity

In our study, although the funnel plot demonstrated relatively good symmetry, and both the Egger’s and Peters’ tests indicated no systematic small-study effects, the Begg’s test still showed mild asymmetry. This suggests that certain individual studies might have been influenced by factors such as sample size, research quality, or publication bias. However, considering the combined results of these three tests, there is insufficient evidence to conclude that publication bias systematically affects the overall findings.

Furthermore, meta-regression results revealed that macro-level covariates, including geographic region, age, population characteristics, and year of publication, provided an extremely limited explanation for the observed heterogeneity in prevalence estimates (*R*^2^ close to 0%). Even the most influential covariate, diagnostic method, accounted for less than 10% of the heterogeneity. This indicates that global *Helicobacter pylori* prevalence is largely influenced by unmeasured contextual factors, such as socioeconomic structures, regional healthcare infrastructure, dietary culture, background antibiotic usage patterns, racial/genetic characteristics, and household size patterns.

These findings underscore the necessity for future research to collect information at a more granular level (e.g., household, community, or individual behavioral factors) to more comprehensively explain the substantial global variation in prevalence.

In summary, while this study provides a multi-continental prevalence estimate, differences in diagnostic methods and unmeasured contextual factors remain key sources affecting comparability. Future epidemiological studies should explicitly specify the diagnostic methods used in their design and reporting and promote the standardization of surveillance systems to enhance the consistency of global prevalence estimates.

### Consistent direction but non-significant SDoH associations: insufficient evidence and methodological limitations

In investigating the associations between *Helicobacter pylori* infection and SDoH, most adverse SDoH factors—such as household crowding, occupational instability, low socioeconomic status, rural residence, unsafe sanitation, and unsafe drinking water—showed a consistent direction of association with a higher risk of *H. pylori* infection. Although pooled ORs for several SDoH domains (e.g., place of residence, sanitation, socioeconomic status) were greater than 1, these associations were not statistically significant. The non-significant results likely reflect the combined influence of multiple factors—including substantial between-study heterogeneity, inconsistent definitions of SDoH, variations in measurement quality, and limited statistical power in certain subgroups—rather than indicating a true absence of effect. Therefore, these findings suggest that the evidence in these domains remains insufficient and highlights the need for further well-designed studies with standardized measurement of SDoH.

### Household crowding, occupational instability, and marital structure: the strongest determinants of infection among SDoH

Among the various SDoH factors examined, the associations for household crowding and occupational instability emerged as the most robust and statistically significant. Several large-scale, community-based studies have also reported familial clustering of *Helicobacter pylori* infection, with evidence of concordant bacterial strains among family members (30, 31). Regarding occupational instability, existing evidence indicates that such instability can lead to increased stress load and a heightened physiological stress state, thereby elevating the risk of infection in affected individuals (32, 33). Notably, our study observed a marginally protective trend associated with adverse marital status (single/widowed/living alone). This may be explained by the established intrafamilial and spousal transmission routes of *H. pylori*: marriage or cohabitation expands daily close-contact networks and prolongs cohabitation exposure, thereby increasing opportunities for acquiring and sustaining infection. Conversely, individuals who are single or live alone experience fewer household contacts, exhibiting a relative protective trend. A large-scale, nationwide, family-based epidemiological study identified family members as the primary source of infection for *H. pylori* patients (30). Molecular subtyping further confirms the existence of within-household transmission channels, as evidenced by the presence of highly related strains (34).

### The gradient effect of education level: its core role as an upstream social determinant

A key finding of this study is the clear and consistent gradient relationship between education level and *Helicobacter pylori* infection, which remains stable across multiple regions and study settings. Although educational attainment was not statistically significant in the main analysis, a distinct gradient was observed in subgroup analyses: the risk was highest for low versus high education (OR = 3.49, 95% CI: 2.98–4.08), followed by low versus middle education (OR = 1.60, 95% CI: 1.38–1.86). This suggests that education may serve as a converging indicator for upstream SDoH.

First, lower education levels are often associated with greater occupational instability and a higher proportion of informal employment. Individuals with less formal education are more likely to work in high-density, high-contact environments such as construction, manufacturing, and service industries. These occupational settings, coupled with living conditions such as household crowding, can increase the frequency of close interpersonal contact and elevate opportunities for *Helicobacter pylori* transmission (35, 36).

Second, individuals with lower educational attainment are more likely to experience low income or lack health insurance. This not only affects living conditions (e.g., sanitation facilities and water quality) but also limits timely access to *H. pylori* diagnosis and eradication therapy, thereby prolonging infection duration and increasing the likelihood of transmission (37).

Third, education influences health literacy and related health behaviors concerning *H. pylori*. Studies indicate that individuals with lower education levels are less knowledgeable about preventive measures such as food hygiene, water safety, and household sanitation, making them more likely to be exposed to unsafe drinking water, unimproved sanitation, and generally poorer household hygiene conditions (38).

Fourth, there is a strong association between education and healthcare access. Those with lower education face disadvantages in healthcare navigation, financial capacity, and healthcare-seeking willingness. They are less likely to undergo *H. pylori* testing and more likely to delay eradication treatment, thereby increasing the risk of persistent infection and intrafamilial transmission (39).

Fifth, education serves as a converging social indicator that often coexists with multiple adverse SDoH factors, including crowding, low socioeconomic status, occupational instability, and poor sanitary environments. These factors collectively increase infection risk through shared mechanisms, primarily *via* oral-oral and fecal-oral transmission routes (40).

This finding carries important implications for public health strategy. In resource-limited settings, priority should be given to populations with lower education levels. Comprehensive interventions—including health education, improvement of sanitation facilities, occupational risk management, and enhanced healthcare access—should be implemented to reduce the sustained transmission of *Helicobacter pylori*.

### Context-dependency and the dominant role of regional variation in SDoH–*Helicobacter pylori* associations

Subgroup analyses revealed a marked context-dependency in the associations between SDoH and *Helicobacter pylori* infection. The relationship between education level and *H. pylori* varied across different regions, populations, and diagnostic methods. The effect of occupational status was more stable in European studies and in those using the UBT for diagnosis. The association with sanitation status was stronger in populations with gastrointestinal symptoms. Socioeconomic status showed a more pronounced correlation in non-pediatric populations, while marital and residential status were overall non-significant in stratified analyses.

Meta-regression further identified geographic region as the sole stable moderator. Publication year showed only a trend-level association, whereas diagnostic method, pediatric status, and the presence of gastrointestinal symptoms were not major explanatory sources for the observed heterogeneity. This aligns with prior global evidence indicating substantial regional disparities in *Helicobacter pylori* prevalence, while acknowledging that factors such as diagnostic approach, age group, and symptomatic status can also influence detection rates (1, 5).

In summary, future risk management strategies should be stratified by geographic region and integrally consider SDoH. This approach involves implementing screen-treat-follow-up protocols for high-risk groups, promoting upstream interventions to address adverse factors such as household crowding and occupational hygiene, and encouraging the simultaneous testing and treatment of household members. These concerted efforts are essential for effectively reducing the burden of *Helicobacter pylori* infection and its related clinical outcomes.

### Sources of extreme heterogeneity and limitations of the current analytical framework

Finally, this study has several limitations. First, we observed extremely high heterogeneity in most models (*I*^2^ typically exceeding 90–99%), while our pre-specified subgroup analyses and exploratory meta-regressions explained only a minimal proportion of this variance (pseudo-*R*^2^ mostly <7%). This suggests that the sources of heterogeneity extend far beyond the measured study-level characteristics. The primary reasons likely include the following aspects: first, the included studies were conducted in vastly different social, economic, familial, and healthcare contexts. Unmeasured background factors (e.g., cultural practices, intergenerational living patterns, and healthcare accessibility) may profoundly influence *H. pylori* exposure and transmission routes, thereby creating systematic differences between studies. Second, *H. pylori* transmission itself is likely affected by a complex interplay of coexisting factors (e.g., population density, family structure, and local health and education systems). These critical contextual variables were not systematically measured in most primary studies, making it difficult for our models to capture the true driving factors.

Furthermore, definitions and thresholds for factors like household crowding, socioeconomic status, education level, and occupational instability varied across studies. To enhance comparability, we dichotomized these exposures, but this approach may have led to loss of information. The original studies employed highly diverse categorizations for SDoH indicators—such as years of education, household crowding indices, and income quantiles—often splitting the same variable into multiple levels or modeling it as continuous.

It should be noted that the strategy of dichotomizing SDoH variables was adopted to address the substantial heterogeneity in their definitions and measurement. This methodological choice is not novel to our study, but it is a common practice in epidemiological research when synthesizing evidence on complex social exposures. For instance, in a national cohort study on premature mortality among US adults by Bundy et al., eight SDoH factors (e.g., employment, household income, education) were similarly dichotomized into favorable or unfavorable categories, successfully analyzing their dose–response relationship with mortality risk. Another large-scale study of nearly 500,000 patients also clearly demonstrated a descending gradient in *Helicobacter pylori* infection rates with rising socioeconomic status by stratifying education and income. These examples illustrate that, given the inherent heterogeneity in existing data, standardized dichotomization of SDoH constitutes a valid and widely accepted analytical framework for enabling cross-study quantitative comparisons and revealing macro-level associations (8, 41).

Nonetheless, methodological studies indicate that dichotomization results in a loss of approximately 33% of information content and reduces statistical power by 36–66% (42, 43). Analysis of real data by Royston et al. further found that dichotomization can shift effect sizes (e.g., ORs) by 30–50% and reduce model explanatory power by 40% (16217841). Additionally, non-differential exposure misclassification can dilute effect sizes toward the null (OR = 1) by 20–50% (44, 45). This evidence suggests that while our dichotomization and re-mapping enhanced cross-study comparability, it may also have attenuated social gradient effects, obscured dose–response relationships, and increased uncertainty around the effect estimates. Future efforts should focus on promoting the standardization of SDoH indicators and on exploring composite indices (e.g., crowding index, SES composite scores) in study reporting to reduce resultant heterogeneity.

The factors mentioned above collectively imply that the generalizability of the pooled effect estimates from this study across different contexts should be interpreted with caution. They underscore the need for future research to employ standardized SDoH measurement frameworks, improve exposure measurement precision, and conduct multi-center studies across diverse social and cultural settings. This will better delineate the true role of these factors in *H. pylori* infection risk and reduce unexplained heterogeneity.

Second, the literature included in this meta-analysis consisted predominantly of cross-sectional studies, and the populations in many of these studies may not be fully representative. Therefore, prioritizing prospective household/community cohorts and multi-center follow-up studies in the future would enhance the explanatory power of the findings.

Third, there was heterogeneity in outcome measurement. Although we conducted analyses across four pre-specified subgroups, the number of studies available for certain specific populations (e.g., older adult(s)) was limited, resulting in insufficient statistical power for more refined stratification. Future studies should consider stratified analyses within larger cohorts—for example, by nesting older adult(s) subgroup studies—and establish continuously updated evidence bases for underrepresented populations.

Fourth, to maximize cross-study comparability, this analysis pooled only cORs calculated from original 2 × 2 frequency data, not adjusted odds ratios (aORs). This constitutes a significant limitation. By not harmonizing and incorporating adjusted effect estimates (aORs) from individual studies, potential key confounders—including age structure, prior comorbidities, household exposure patterns, lifestyle factors (e.g., smoking), and access to social resources—may have influenced the results in varying directions and magnitudes across studies, leading to over- or underestimation of the true effect. Moreover, the contribution of several large studies reporting well-adjusted aORs was relatively weakened within our cOR framework, potentially affecting the robustness of the overall inference. Consequently, future studies should report both crude and adjusted effect measures, or employ more comparable effect metrics (e.g., prevalence ratios, risk differences), combined with quantitative bias analysis, to more comprehensively assess the impact of unmeasured confounding on the true association.

Fifth, another limitation is that, although we explored potential sources of heterogeneity *via* meta-regression, the pseudo-*R*^2^ values of the models were generally low. This indicates that other important, unmeasured factors—such as genetic background, dietary patterns, or more nuanced sociocultural variables—might be driving the substantial between-study differences. Therefore, future research needs to incorporate a wider range of explanatory variables to further probe the unexplained heterogeneity.

Sixth, the published evidence and our sample were highly concentrated in East Asia (primarily China), with relative under-representation from regions such as Africa and Latin America. Future efforts should involve cross-regional collaboration to increase samples from underrepresented areas and establish multi-center platforms to mitigate the influence of confounding factors.

## Conclusion

This systematic review and meta-analysis indicate that the global pooled prevalence of *Helicobacter pylori* infection is approximately 43%. The estimated prevalence varied significantly according to the diagnostic method used, underscoring the need for standardized diagnostic protocols in future research. Under a unified mapping framework for SDoH, most adverse SDoH factors demonstrated a consistent direction of association with a higher risk of infection. Among these, household crowding and occupational instability emerged as the most robust risk factors. The influence of education level displayed a clear social gradient, with risk decreasing from lower to higher levels of educational attainment. Adverse marital status (e.g., divorced/widowed) may confer a marginally protective effect against *H. pylori* infection.

Integrated subgroup and meta-regression analyses identified geographic region as a stable moderator of the strength of these associations. Consequently, future strategies for *Helicobacter pylori* prevention and control should pivot from a uniform approach to a stratified management model guided by SDoH and tailored to regional epidemiological patterns. Strengthening the case-finding component in this manner is crucial to maximizing the public health impact of effective treatment and achieving a feasible and sustainable eradication goal. Simultaneously, standardizing diagnostic procedures and reporting guidelines is imperative to enhance the comparability of evidence and the quality of program evaluation. These combined efforts will accelerate the reduction of both primary infections and reinfections, ultimately alleviating the global burden of *Helicobacter pylori*-related diseases.

## Data Availability

The original contributions presented in the study are included in the article/Supplementary material; further inquiries can be directed to the corresponding author/s.
