# Supplementary material for: Social determinants of health and Helicobacter pylori infection prevalence: a systematic review and meta-analysis
Source: Front Public Health. 2026 Jan 13;13:1703158. doi: 10.3389/fpubh.2025.1703158 (PMC12835281; doi:10.3389/fpubh.2025.1703158)
Supplement: Supplementary file 1 [file Supplementary_file_1.docx]

**Supplementary Methods**

1. Variable Remapping

This meta-analysis used only crude effect sizes—specifically, crude odds ratios (cORs) calculated from original 2×2 frequency tables—for pooling associations. Adjusted effect estimates (aORs) were neither collected nor pooled. For each study and each comparable exposure-control pair, the odds ratio (OR) and its 95% confidence interval (CI) were calculated using the logit method. The Haldane-Anscombe continuity correction (adding 0.5 to all cells) was applied to handle zero cells.

To achieve cross-study comparability, all social determinants of health (SDoH) variables were uniformly remapped into binary categories (e.g., favorable vs. unfavorable; rural vs. urban) according to pre-specified rules (see Supplementary Tables S2–S3). The crude OR (cOR) and its 95% CI were then calculated based on these standardized 2×2 tables. The remapping and subgroup classification rules for the education variable are detailed in a separate supplementary methods file.

Remapping continuous or multi-categorical SDoH variables into a binary format was a pre-defined and necessary step in this meta-analysis. Its primary purpose was to address the substantial heterogeneity across original studies in SDoH definitions, measurement scales, and grouping thresholds. This approach established a unified and comparable analytical framework for defining "adverse exposure," enabling quantitative synthesis across studies. We acknowledge that this process entails a loss of information from the original data (e.g., the inability to analyze dose-response relationships or assess gradient effects within a category). However, this was a necessary trade-off to facilitate the integration of evidence on a broad scale while retaining a sufficient number of studies for analysis.

Education was treated as the sole exception. For the main analysis, the comparison was "low education vs. medium/high education (combined)." Additionally, two pre-specified subgroup analyses were conducted: ① low vs. medium education, and ② low vs. high education. If a single study reported multiple education levels or categorizations, data were included only once according to the above priority order to avoid double-counting. All other SDoHs, even if originally reported as multi-level or continuous variables in the source literature, were dichotomized based on the thresholds listed in Supplementary Tables S2–S3 before being included in the pooled analysis.

1. Subgroup Definitions

Subgroup analyses in this study were defined as follows:

Subgroup 1 (Region): Studies were classified by geographic region: Africa, Asia, Europe, and Latin America.

Subgroup 2 (Age): Study samples were categorized as either children or non-children.

Subgroup 3 (Population Characteristics): Populations were grouped based on the presence or absence of gastrointestinal symptoms.

Subgroup 4 (Diagnostic Method): Studies were stratified by the primary Helicobacter pylori diagnostic method reported (e.g., UBT, stool antigen test, serology, histology/culture/PCR). Studies employing multiple methods were categorized as "Mixed (Mixing)."

To ensure robustness, quantitative pooling within a diagnostic method subgroup was performed only when at least 2 studies were available (k ≥ 2). Categories with an insufficient number of studies or sample size (e.g., a single study) or those providing very low information (e.g., "Not reported" or only RUT) were excluded from quantitative synthesis. Their potential contribution to heterogeneity was acknowledged in the discussion.

3. Quality Assessment

The methodological quality of the included studies was assessed using the Newcastle-Ottawa Scale (NOS). The standard NOS was used for cohort and case-control studies. For cross-sectional studies, which reported both prevalence data and exposure-outcome association data (2×2 tables), two adapted versions were employed: the NOS-xs (0–9 points) for assessing the quality of association analyses, and the NOS-xs2 (0–4 points) for assessing the quality of prevalence estimates.

The pre-defined quality threshold for inclusion in the main analysis was a score of NOS/NOS-xs ≥ 6 points and NOS-xs2 ≥ 3 points. Studies scoring below these thresholds were excluded from the primary meta-analysis but were considered for narrative synthesis in the systematic review. Consequently, this meta-analysis included only studies meeting the score of 6 points or higher.

The risk of bias assessment was conducted independently by two investigators. Any disagreements were resolved through discussion or, if necessary, by arbitration from a third investigator to ensure consistency.

4. Statistical Methods

4.1. Pooling of Prevalence

The pooled prevalence of Helicobacter pylori was calculated using a random-effects model, employing the Paule-Mandel estimator for τ² (between-study variance) with Hartung-Knapp adjustment. Prevalence proportions were transformed using the Freeman-Tukey double arcsine transformation prior to pooling and then back-transformed for presentation. This combination of methods was selected for its robustness in settings characterized by high heterogeneity and potential small-study effects. Specifically, the Hartung-Knapp adjustment accounts for the uncertainty in the estimate of between-study variance, providing more conservative and reliable confidence intervals when heterogeneity is substantial and the number of studies is limited. The Freeman-Tukey transformation was chosen for its superior stability when handling proportions with extreme values (near 0% or 100%) or studies with markedly different sample sizes, effectively stabilizing variances and mitigating potential bias associated with traditional methods (e.g., simple logit transformation) on such data.

4.2. Pooling of Associations (SDoH → Helicobacter pylori)

Crude odds ratios (cORs) were calculated from original 2×2 frequency tables. Associations were pooled using the Mantel-Haenszel method under a random-effects model framework (incorporating Hartung-Knapp adjustment and Paule-Mandel τ²), chosen for its favorable statistical properties when analyzing sparse data common in meta-analyses with numerous small-sample studies. The Haldane-Anscombe continuity correction was applied to address zero cells, ensuring the numerical stability of effect size calculations.

4.3. Subgroup Analysis

Pre-specified subgroup analyses were conducted by geographic region, age group, population type, and Helicobacter pylori diagnostic method. Quantitative synthesis within a subgroup was performed only when at least two studies were available (k ≥ 2). It is important to note that all these subgroup comparisons, along with the subsequent meta-regression, are considered exploratory analyses. Their corresponding p-values were not adjusted for multiple testing, and the results should be interpreted as hypothesis-generating rather than confirmatory.

4.4. Meta-Regression (Exploratory)

Exploratory univariable meta-regression was performed using the metafor::rma.uni function. The significance of regression coefficients was assessed via two-sided Wald (z) tests. Covariates, including publication year (treated as a continuous variable) and the variables defining Subgroups 1–4, were entered into the models one at a time. The proportion of explained heterogeneity was quantified by the pseudo R² statistic, calculated for each model on the available subsample of studies with complete data for that covariate using the formula: R² = (τ²_null − τ²_model) / τ²_null × 100%. The results are intended for hypothesis generation, and no correction for multiple testing was applied.

**Supplementary Results**

1. Helicobacter pylori Prevalence

1.1 Subgroup Analysis of Prevalence (Supplementary Figures 1-4)

Subgroup 1 (Geographic Region): The pooled prevalence was 47% (95% CI: 36%–57%) in Africa, 44% (95% CI: 34%–54%) in Asia, 40% (95% CI: 20%–62%) in Europe, and 44% (95% CI: 21%–69%) in Latin America. The test for subgroup differences was not statistically significant (χ² = 0.57, p = 0.903).

Subgroup 2 (Children vs. Non-Children): The prevalence was 40% (95% CI: 26%–56%) among children and 45% (95% CI: 38%–53%) among non-children. No significant difference was found between these age groups (χ² = 0.43, p = 0.512).

Subgroup 3 (Gastrointestinal Symptoms): The prevalence was 43% (95% CI: 31%–56%) in populations with symptoms and 45% (95% CI: 37%–52%) in asymptomatic/general populations. The difference was not statistically significant (χ² = 0.02, p = 0.878).

Subgroup 4 (Diagnostic Method): The prevalence estimates varied significantly by diagnostic method: 52% (95% CI: 24%–80%) for stool antigen tests, 39% (95% CI: 14%–67%) for mixed methods, 49% (95% CI: 45%–53%) for serology, and 46% (95% CI: 36%–56%) for urea breath tests (UBT). The test for subgroup differences was highly significant (χ² = 432.65, p < 0.0001).

1.2 Meta-Regression of Prevalence

A univariable meta-regression was performed on 57 studies with complete covariate information using the REML method with Knapp-Hartung adjustment. Publication year and the four pre-specified stratification variables (geographic region, age group, population characteristics based on symptoms, and diagnostic method) were examined individually as covariates.

The results showed that only the diagnostic method (Subgroup 4) explained any notable proportion of heterogeneity, reducing τ² from 0.0556 to 0.0515 (R² = 7.3%). However, this moderating effect did not reach statistical significance (Qm p = 0.114). Publication year, geographic region, age group, and population characteristics explained almost none of the heterogeneity (R² ≈ 0%; Qm p-values ranged from 0.49 to 0.95).

In summary, the pre-specified covariates explained only a minimal fraction of the between-study variance, indicating that more important, unmeasured sources of heterogeneity are likely present.

2. Associations between SDoH and Helicobacter pylori Infection

2.1 Subgroup Analyses of SDoH-Helicobacter pylori Associations

The subgroup analyses reported below are exploratory. All p-values are unadjusted for multiple comparisons. The findings are intended to describe potential sources of heterogeneity and require validation in future studies.

2.1.1 Household Overcrowding

After stratification by geographic region, age, diagnostic method, and symptom status, the direction of association was largely consistent (OR >1) but did not reach statistical significance in any subgroup.

By Region (Supplementary Figure 5): Test for subgroup differences, χ² = 2.32, p = 0.66.

By Age (Children vs. Non-Children) (Supplementary Figure 6): χ² = 0.37, p = 0.54.

By Symptom Status (Supplementary Figure 7): χ² = 0.56, p = 0.46.

By Diagnostic Method (Supplementary Figure 8): χ² = 6.99, p = 0.07.

2.1.2 Drinking Water Source

After stratification, the association direction was broadly consistent (OR >1) but not statistically significant.

By Region (Supplementary Figure 9): Test for subgroup differences, χ² = 7.52, p = 0.06.

By Age (Children vs. Non-Children) (Supplementary Figure 10): χ² = 0.23, p = 0.63.

By Symptom Status (Supplementary Figure 11): χ² = 1.00, p = 0.32.

By Diagnostic Method (Supplementary Figure 12): χ² = 7.52, p = 0.06.

2.1.3 Education

Subgroup analysis revealed a statistically significant interaction by geographic region (Supplementary Figure 13; χ² = 17.68, p = 0.0005). A significant positive association was observed in Europe (OR = 1.67, 95% CI: 1.30–2.15), while associations in Asia, Africa, and Latin America were non-significant. Significant between-subgroup differences were also observed for age (Supplementary Figure 14; χ² = 5.58, p = 0.02), symptom status (Supplementary Figure 15; χ² = 5.95, p = 0.01), and diagnostic method (Supplementary Figure 16; χ² = 7.90, p = 0.048). However, within most of these subgroups, the 95% CIs of the pooled effects crossed 1, indicating inconsistent direction and unstable evidence, particularly in subgroups with fewer studies (e.g., children, stool antigen test).

2.1.4 Marital Status

After stratification by region, diagnostic method, and symptom status (age stratification was not applicable as all included studies focused on adults), the direction of association was largely consistent but not statistically significant in any subgroup.

By Region (Supplementary Figure 17): χ² = 4.82, p = 0.19.

By Symptom Status (Supplementary Figure 18): χ² = 0.02, p = 0.89.

By Diagnostic Method (Supplementary Figure 19): χ² = 0.45, p = 0.80.

2.1.5 Occupational Status

Subgroup analyses identified region, symptom status, and diagnostic method as significant sources of effect variation.

By Region (Supplementary Figure 20): χ² = 17.68, p = 0.0005. The association was significant in Europe (OR = 1.67, 95% CI: 1.30–2.15) but not in other regions.

By Symptom Status (Supplementary Figure 21): χ² = 5.95, p = 0.0147.

By Diagnostic Method (Supplementary Figure 22): χ² = 7.90, p = 0.048. The association was significant for studies using the UBT (OR = 1.27, 95% CI: 1.09–1.48) but not for other methods.

2.1.6 Residence Type

After stratification, the direction of association was largely consistent (OR >1) but not statistically significant.

By Region (Supplementary Figure 23): χ² = 1.46, p = 0.48.

By Age (Supplementary Figure 24): χ² = 1.09, p = 0.30.

By Symptom Status (Supplementary Figure 25): χ² = 3.14, p = 0.08.

By Diagnostic Method (Supplementary Figure 26): χ² = 1.46, p = 0.48.

2.1.7 Sanitation Conditions

Subgroup analyses identified region, symptom status, and diagnostic method as significant sources of effect variation.

By Region (Supplementary Figure 27): χ² = 40.04, p < 0.0001.

By Symptom Status (Supplementary Figure 28): χ² = 10.23, p = 0.0013. A significant association was found in populations with symptoms (OR = 3.59, 95% CI: 1.71–7.56) but not in asymptomatic populations (OR = 0.69, 95% CI: 0.21–2.25).

By Diagnostic Method (Supplementary Figure 29): χ² = 75.29, p < 0.0001.

By Age (Supplementary Figure 30): The difference was not significant (χ² = 0.27, p = 0.60).

2.1.8 Socioeconomic Status

After stratification by region, diagnostic method, and symptom status, associations were directionally consistent but non-significant.

By Region (Supplementary Figure 31): χ² = 4.81, p = 0.19.

By Symptom Status (Supplementary Figure 32): χ² = 0.61, p = 0.44.

By Diagnostic Method (Supplementary Figure 33): χ² = 2.49, p = 0.48.

By Age (Supplementary Figure 34): A significant between-subgroup difference was found (χ² = 5.7, p = 0.02). Lower socioeconomic status was significantly associated with higher odds of Helicobacter pylori infection in non-child populations (OR = 1.38, 95% CI: 1.11–1.73) but not in children (OR = 0.71, 95% CI: 0.39–1.31).

2.2 Meta-Regression of SDoH-Helicobacter pylori Associations

Meta-regression indicated that only geographic region had a statistically significant moderating effect on the SDoH-Helicobacter pylori associations (Qm = 9.64, p = 0.022; Pseudo-R² = 4.6%). Publication year showed a trend toward significance (Qm = 3.59, p = 0.058; Pseudo-R² = 2.29%). Neither diagnostic method (Qm = 2.84, p = 0.417) nor the age group (child vs. non-child) (Qm = 0.71, p = 0.407) showed significant moderating effects. Overall, the low Pseudo-R² values indicate that the examined covariates explain only a minimal proportion of the between-study heterogeneity, with the majority of its sources remaining unaccounted for.
