# Supplementary material for: Social determinants of health and Helicobacter pylori infection prevalence: a systematic review and meta-analysis
Source: Front Public Health. 2026 Jan 13;13:1703158. doi: 10.3389/fpubh.2025.1703158 (PMC12835281; doi:10.3389/fpubh.2025.1703158)
Supplement: Supplementary file 2 [file Supplementary_file_2.docx]

| **Pubmed** | ("Helicobacter pylori"[Mesh] OR "H. pylori infection" OR "Helicobacter infection")  AND  ("Social Determinants of Health"[Mesh]  OR "Social determinants"  OR "Socioeconomic Factors"[Mesh]  OR "Socioeconomic status"  OR "Income"  OR "Employment"  OR "Unemployment"  OR "Education level"  OR "Educational attainment"  OR "Housing"  OR "Overcrowding"  OR "Food security"  OR "Health care access"  OR "Health Services Accessibility"  OR "Social Support"  OR "Social inclusion"  OR "Discrimination"  OR "Neighborhood"  OR "Environmental exposure")  AND (Humans[Mesh])  AND (English[lang])  NOT (Review[ptyp] OR Meta-Analysis[ptyp] OR Case Reports[ptyp] OR Letter[ptyp] OR Clinical Trial[ptyp] OR Systematic Review[ptyp]) |
| --- | --- |
| **Web of Sience** | TS=("Helicobacter pylori" OR "H. pylori infection" OR "Helicobacter infection")  AND  TS=("Social determinants of health" OR "Social determinants" OR "Socioeconomic status" OR "Socioeconomic factors"  OR "Income" OR "Employment" OR "Unemployment"  OR "Education level" OR "Educational attainment"  OR "Housing" OR "Overcrowding" OR "Food security"  OR "Health care access" OR "Health Services Accessibility"  OR "Social support" OR "Social inclusion"  OR "Discrimination" OR "Neighborhood" OR "Environmental exposure")  AND  LA=(English)  AND  DT=(Article)  NOT  DT=("Review" OR "Meta-Analysis" OR "Case Report" OR "Letter" OR "Clinical Trial" OR "Systematic Review") |
| **Embase** | ('helicobacter pylori'/exp OR 'h. pylori infection':ti,ab,kw OR 'helicobacter infection':ti,ab,kw)  AND  ('social determinant of health'/exp OR 'social determinants':ti,ab,kw OR 'socioeconomic factor'/exp OR 'socioeconomic status':ti,ab,kw  OR income:ti,ab,kw OR employment:ti,ab,kw OR unemployment:ti,ab,kw  OR 'education level':ti,ab,kw OR 'educational attainment':ti,ab,kw  OR housing:ti,ab,kw OR overcrowding:ti,ab,kw OR 'food security':ti,ab,kw  OR 'health care access':ti,ab,kw OR 'health services accessibility':ti,ab,kw  OR 'social support':ti,ab,kw OR 'social inclusion':ti,ab,kw  OR discrimination:ti,ab,kw OR neighborhood:ti,ab,kw OR 'environmental exposure':ti,ab,kw)  AND  [english]/lim  AND  [humans]/lim  NOT  ('review'/it OR 'meta analysis'/it OR 'case report'/it OR 'letter'/it OR 'clinical trial'/it OR 'systematic review'/it) |

Supplementary Table 1. Search Strategy Table

| Variable | Beneficial classification | Adverse classification |
| --- | --- | --- |
| Degree of family overcrowding | No family overcrowding, no excessive crowding, small family size, family size < 4 people, number of people per room ≤ 1, living space (square meters per person) (> 40), no shared beds | There is family overcrowding, excessive overcrowding, large family size, family size ≥ 4 people, number of people per room range > 1, living space (square meters per person) (≤ 40), and shared beds exist. |
| Social economic status | Non-manual workers, high income level, least poor in terms of wealth index, high monthly income, affluent family economy, high family income, high annual income, social class (high + medium), high social economic conditions | Manual workers, low income level, wealth index (Poorer + Poorest), low monthly income, poverty in family economy, low family income, low annual income, social class (low), low socio-economic conditions |
| Place of residence | City, urban area, exurban area, town | Rural areas, suburban areas, and urban-rural fringe areas |
| Sanitary conditions | Toilet with a water pump | Pit latrines, open-air, without flush toilets, open area, no sanitation facilities |
| Drinking water safety | Piped water, municipal water supply, safe water source, purified water source, public supply, PAM tap water, boiling water | Plastic oil drums, other sources (not from public supply), surface water, boreholes/wells, river water, unsafe, no piped water, unfiltered water sources, groundwater |
| Marital | Married and having a cohabiting partner | Single, divorced, widowed, separated |
| Occupation status | Civil servants, employees, technicians, students, businessmen, managers | Housewife, farmer, unemployed person, day laborer |

Supplementary Table 2. Mapping Table

| Elementary | Medium | Advanced |
| --- | --- | --- |
| Illiterate, primary school level | Junior high school, high school, senior high school | University, graduate school, doctoral degree, above university level |

Supplementary Table 3. Mapping Table of Educational Variables
