# Supplementary material for: Social determinants of health and Helicobacter pylori infection prevalence: a systematic review and meta-analysis
Source: Front Public Health. 2026 Jan 13;13:1703158. doi: 10.3389/fpubh.2025.1703158 (PMC12835281; doi:10.3389/fpubh.2025.1703158)
Supplement: Supplementary file 3 [file Supplementary_file_3.docx]

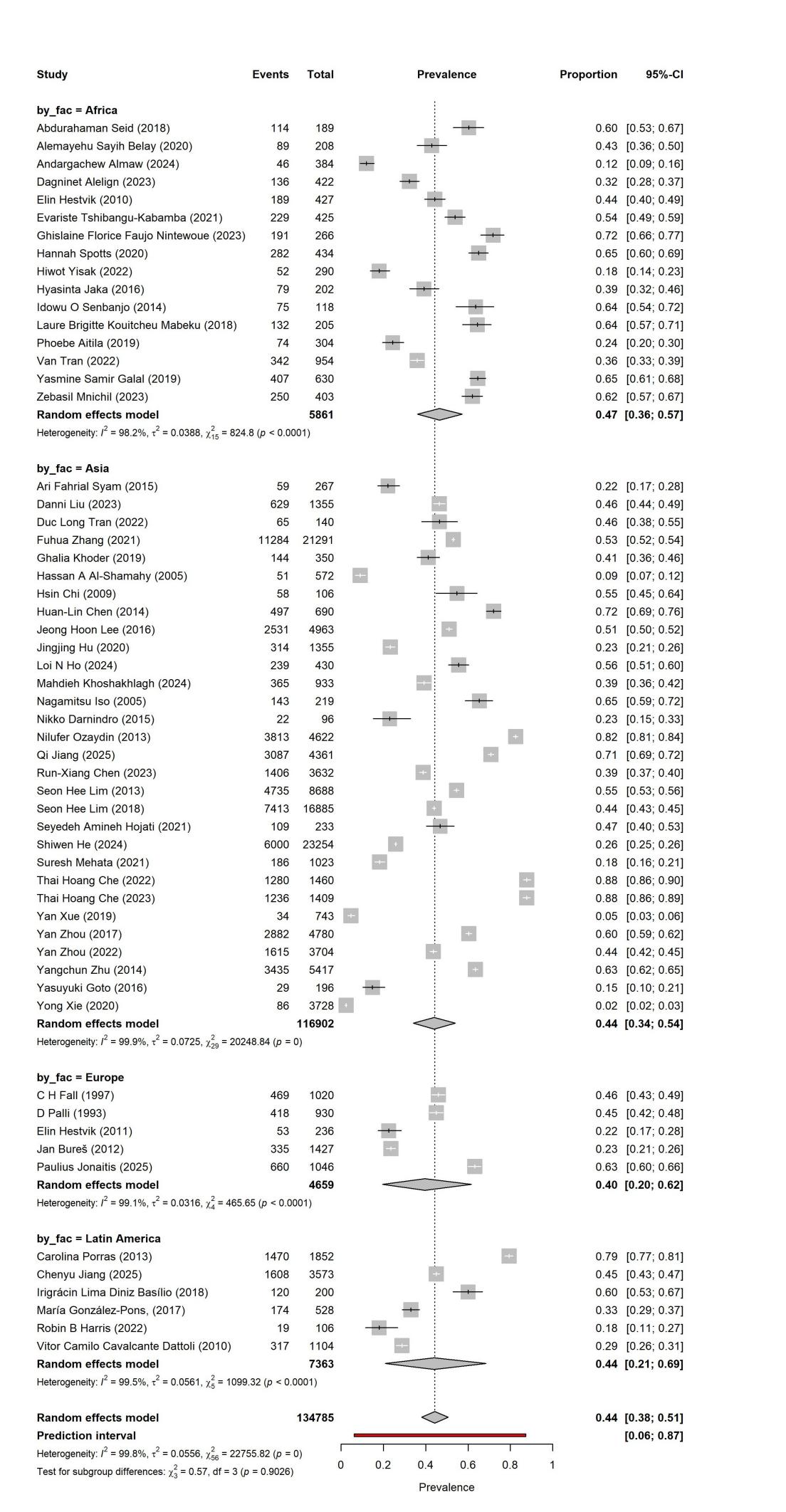


Supplementary Figure 1 . Subgroup meta-analysis of infection rates (Subgroup 1)


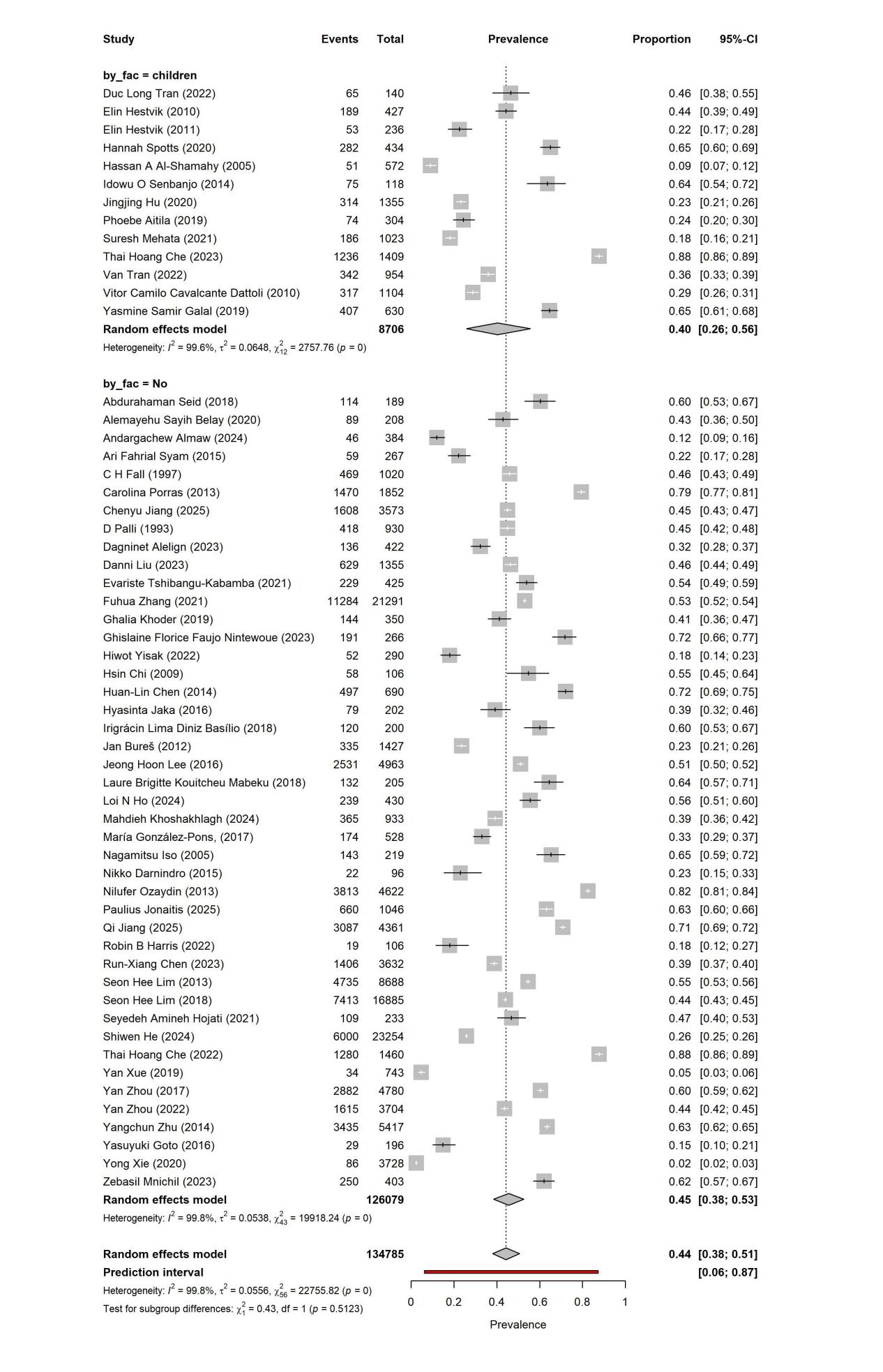


Supplementary Figure 2. Subgroup meta-analysis of infection rates (Subgroup 2)


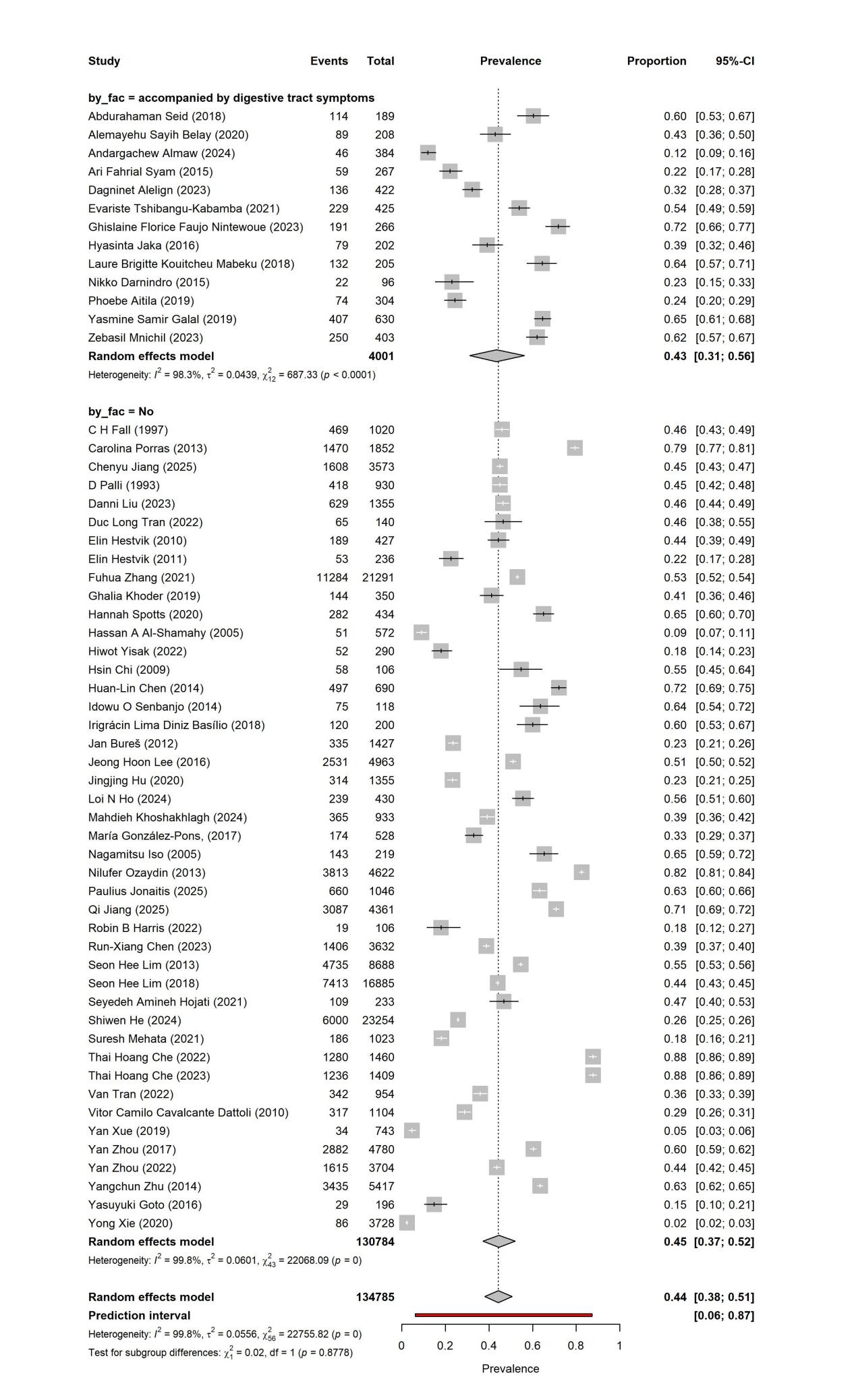


Supplementary Figure 3. Subgroup meta-analysis of infection rates (Subgroup 3)


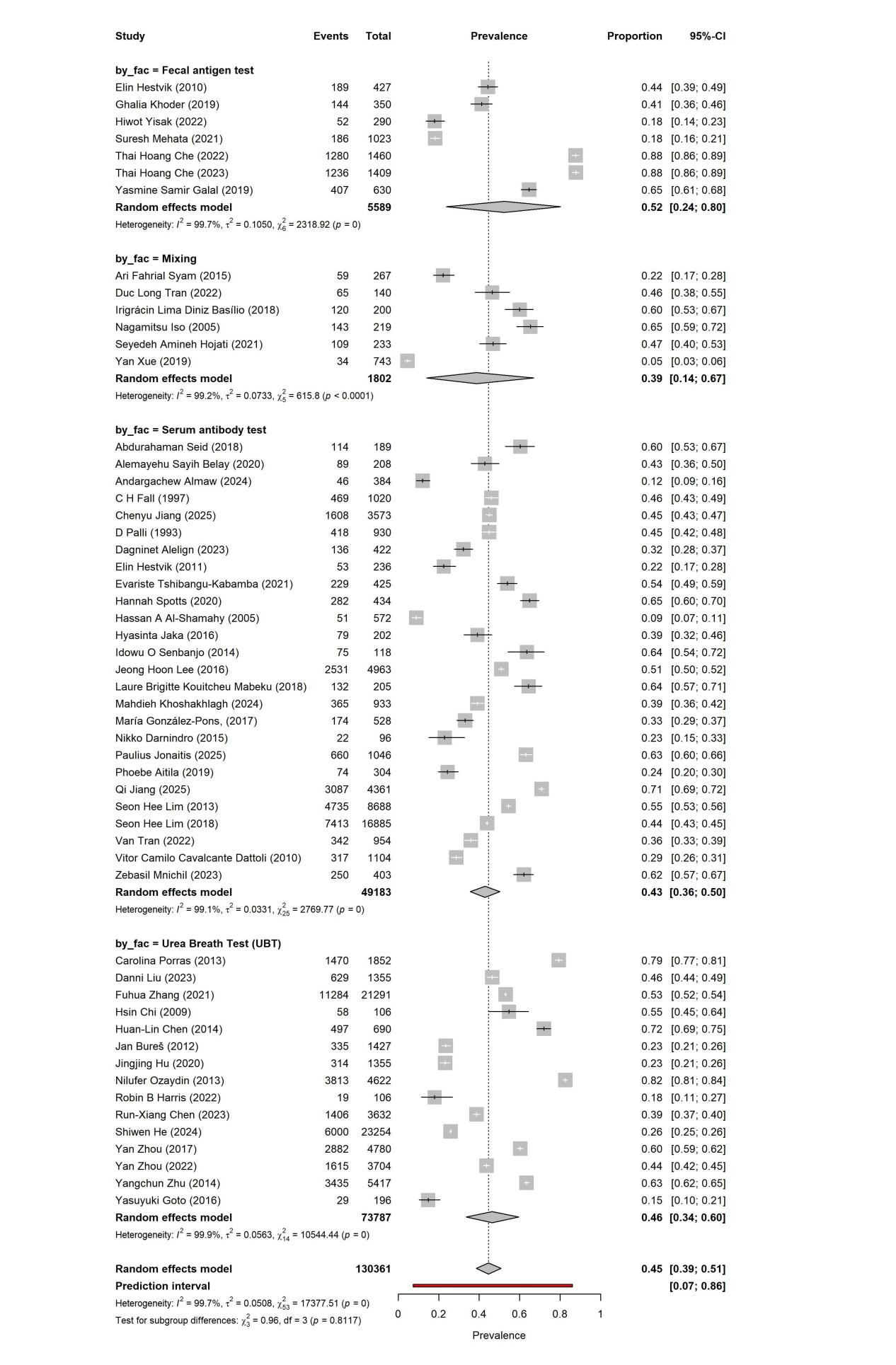


Supplementary Figure 4. Subgroup meta-analysis of infection rates (Subgroup 4)


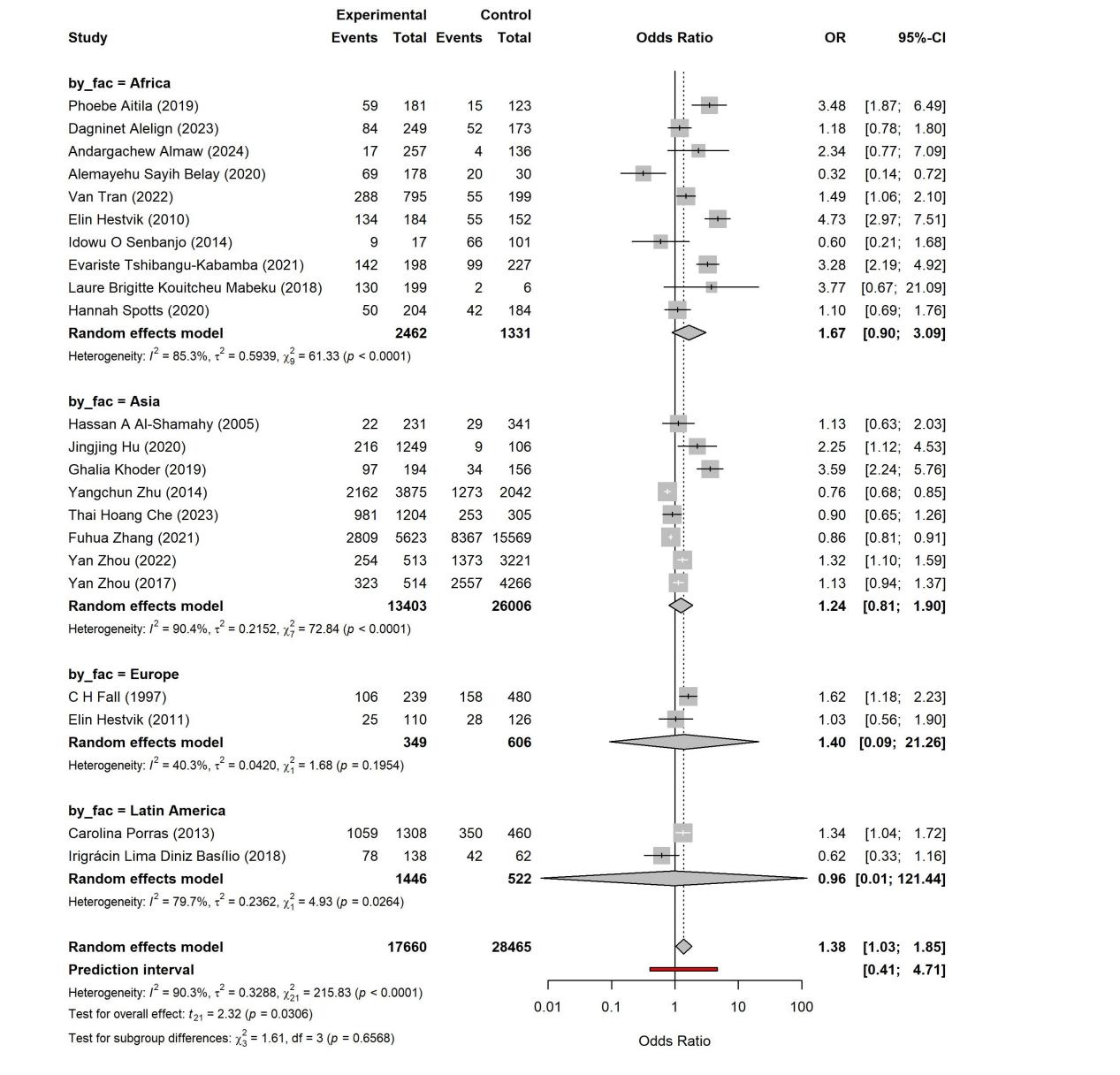


Supplementary Figure 5. Forest plot of household overcrowding - Helicobacter pylori infection (subgroup: region)


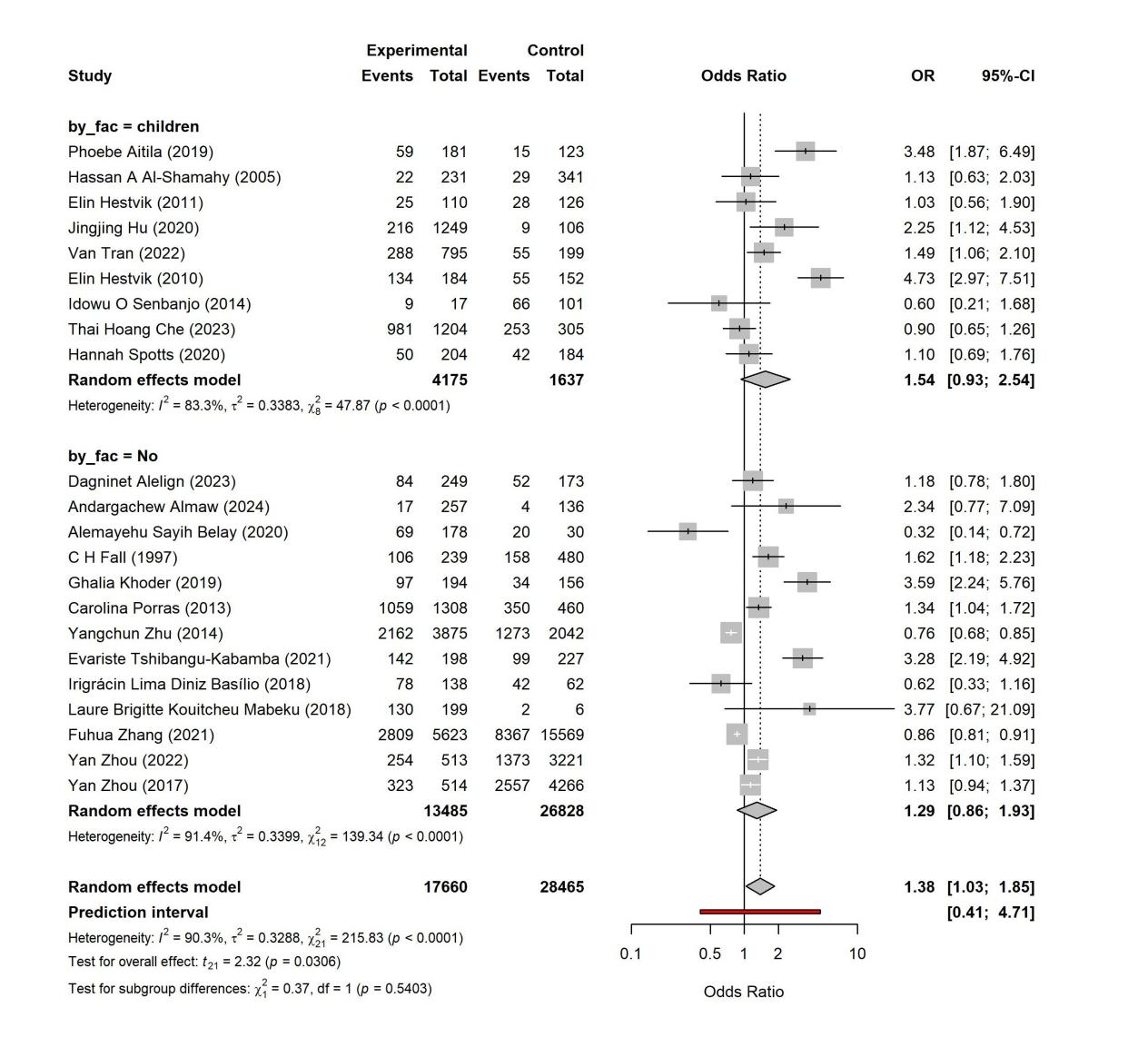


Supplementary Figure 6. Forest plot of household overcrowding - Helicobacter pylori infection (subgroup: age)


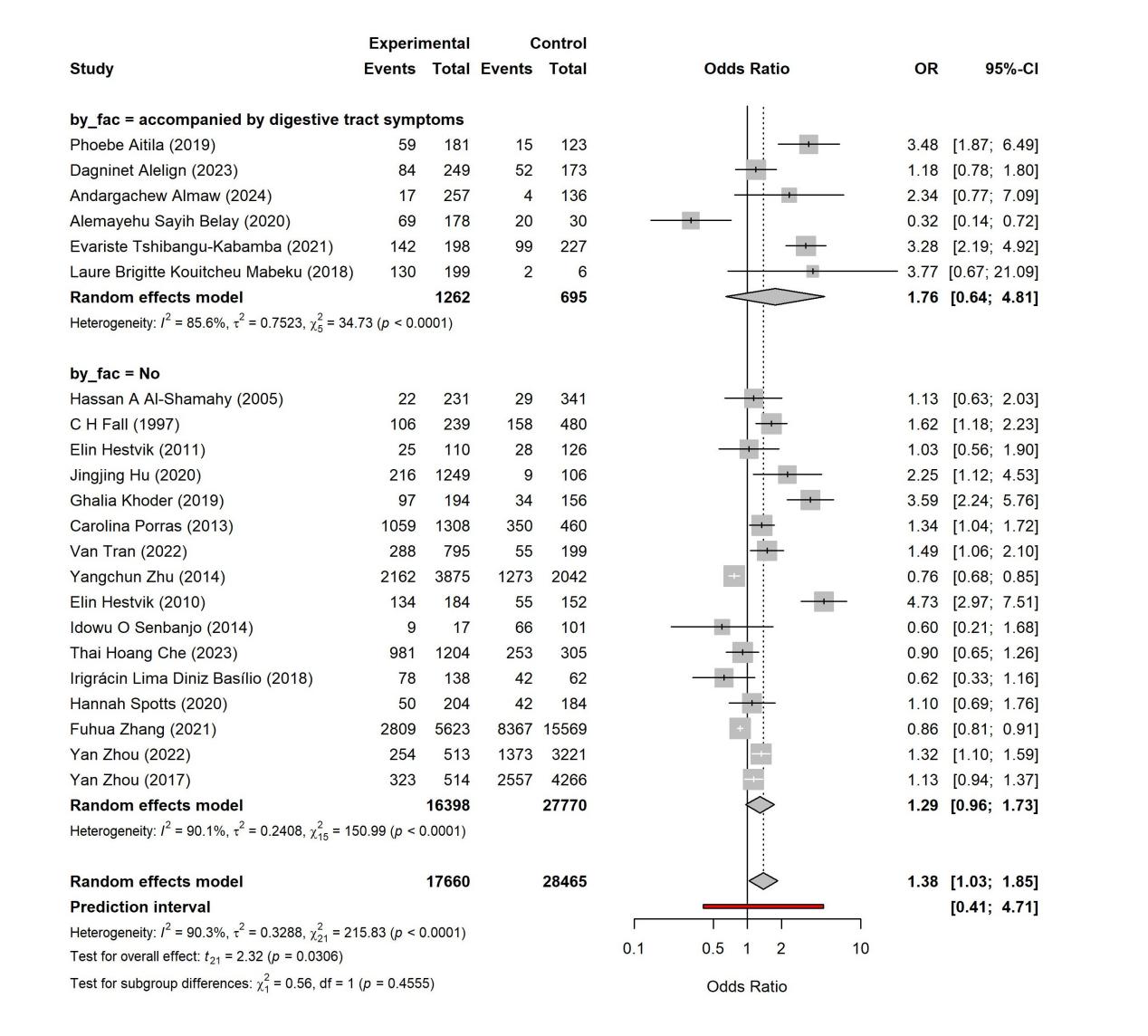


Supplementary Figure 7. Forest plot of household overcrowding - Helicobacter pylori infection (Subgroup: Gastrointestinal symptoms)


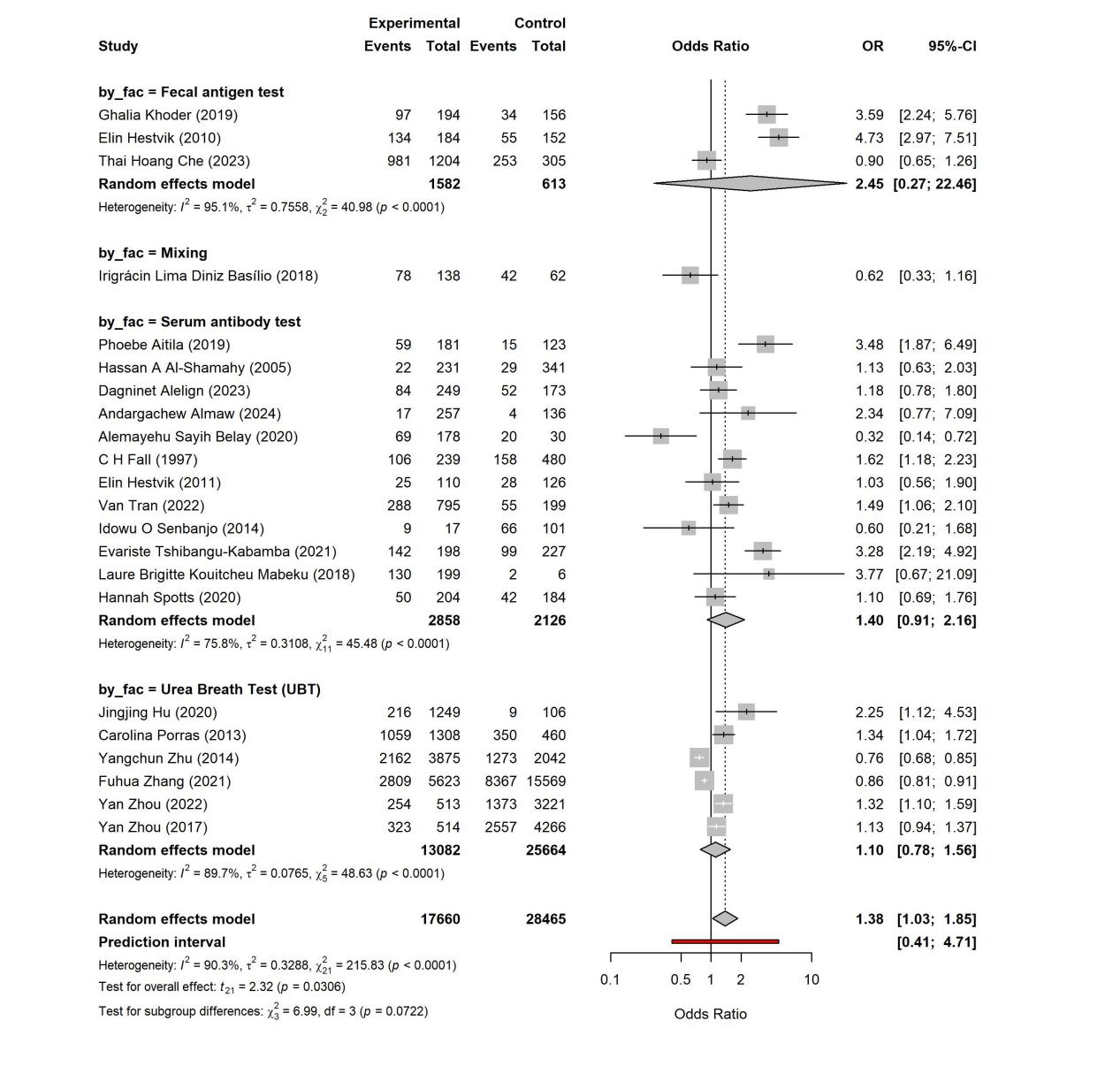


Supplementary Figure 8. Forest plot of household overcrowding - Helicobacter pylori infection (Subgroup: Testing method)


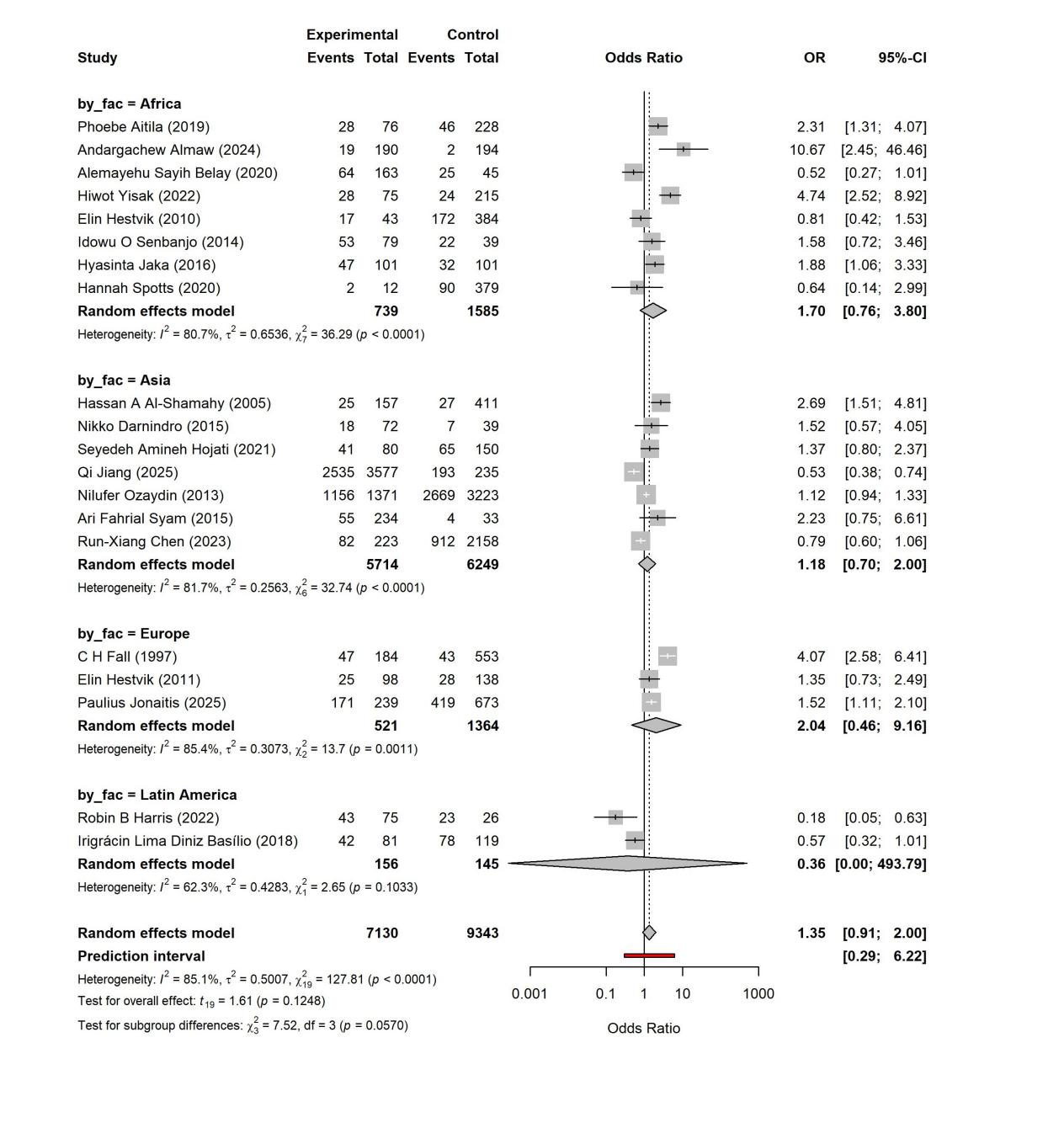


Supplementary Figure 9. Forest plot of drinking water safety - Helicobacter pylori infection (subgroup: region)


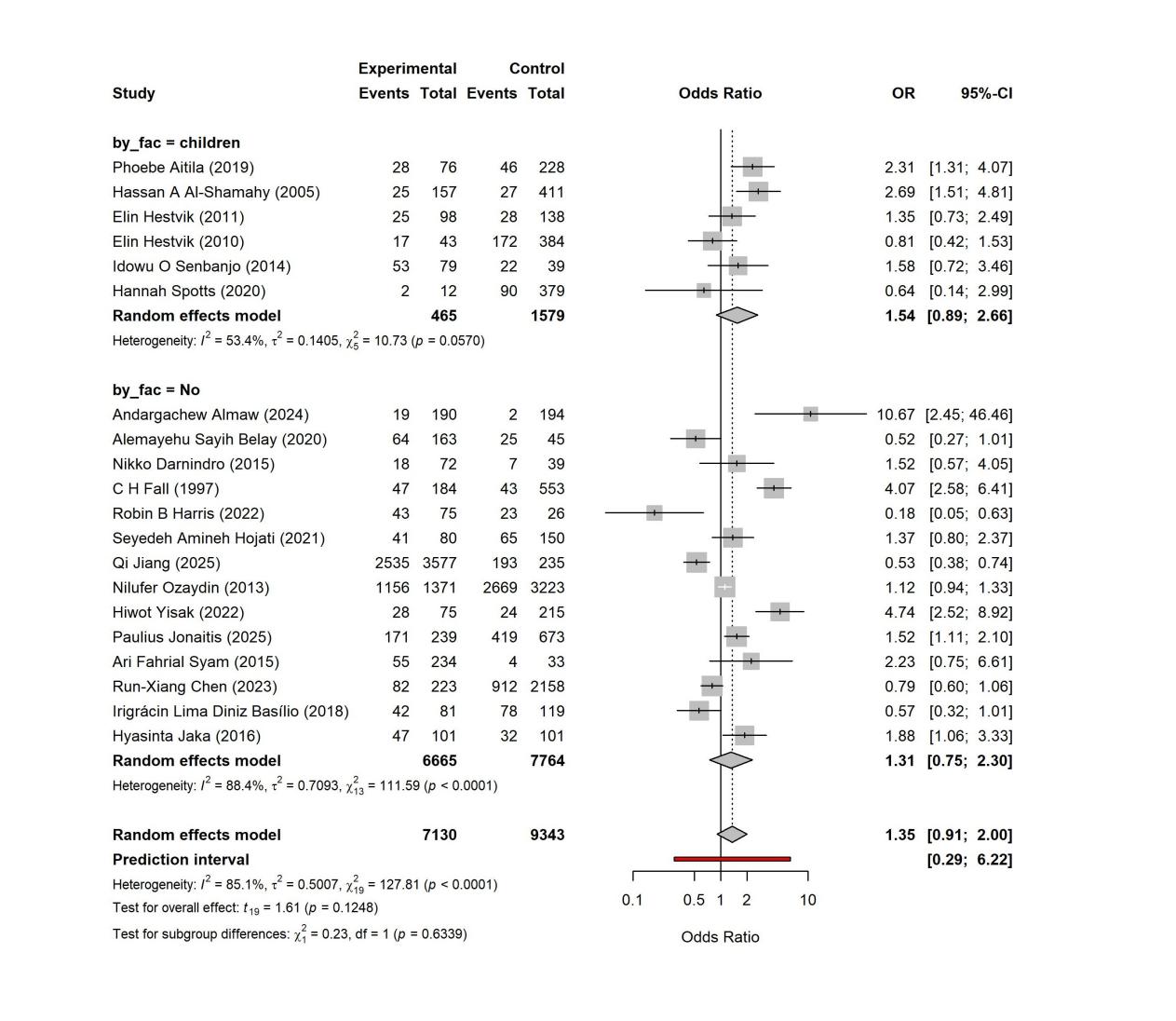


Supplementary Figure 10. Drinking Water Safety - Forest Plot for Helicobacter pylori Infection (Subgroup: Age)


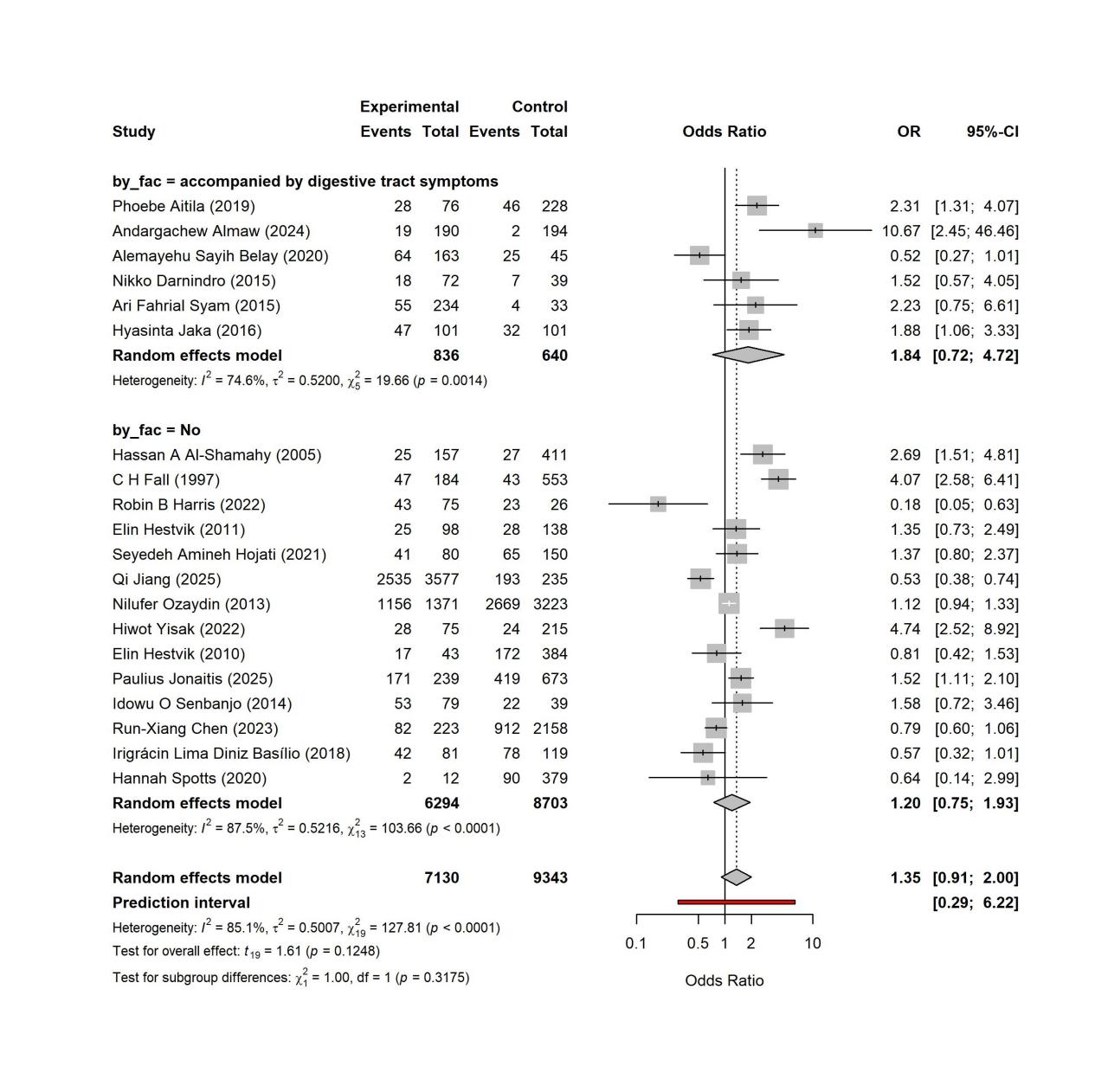


Supplementary Figure 11. Forest plot for drinking water safety - Helicobacter pylori infection (Subgroup: Gastrointestinal symptoms)


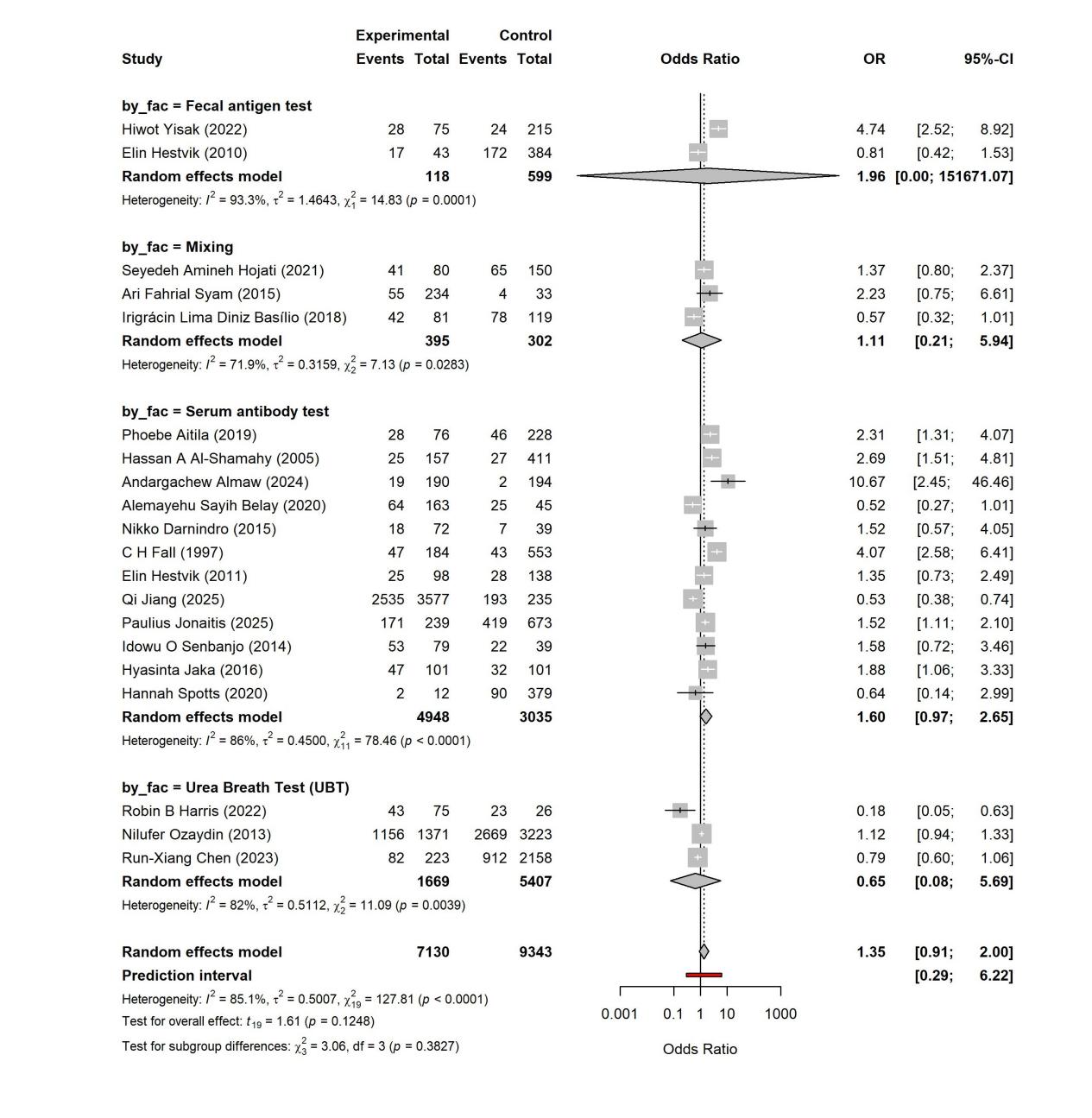


Supplementary Figure 12. Drinking Water Safety - Forest Plot for Helicobacter pylori Infection (Subgroup: Testing Method)


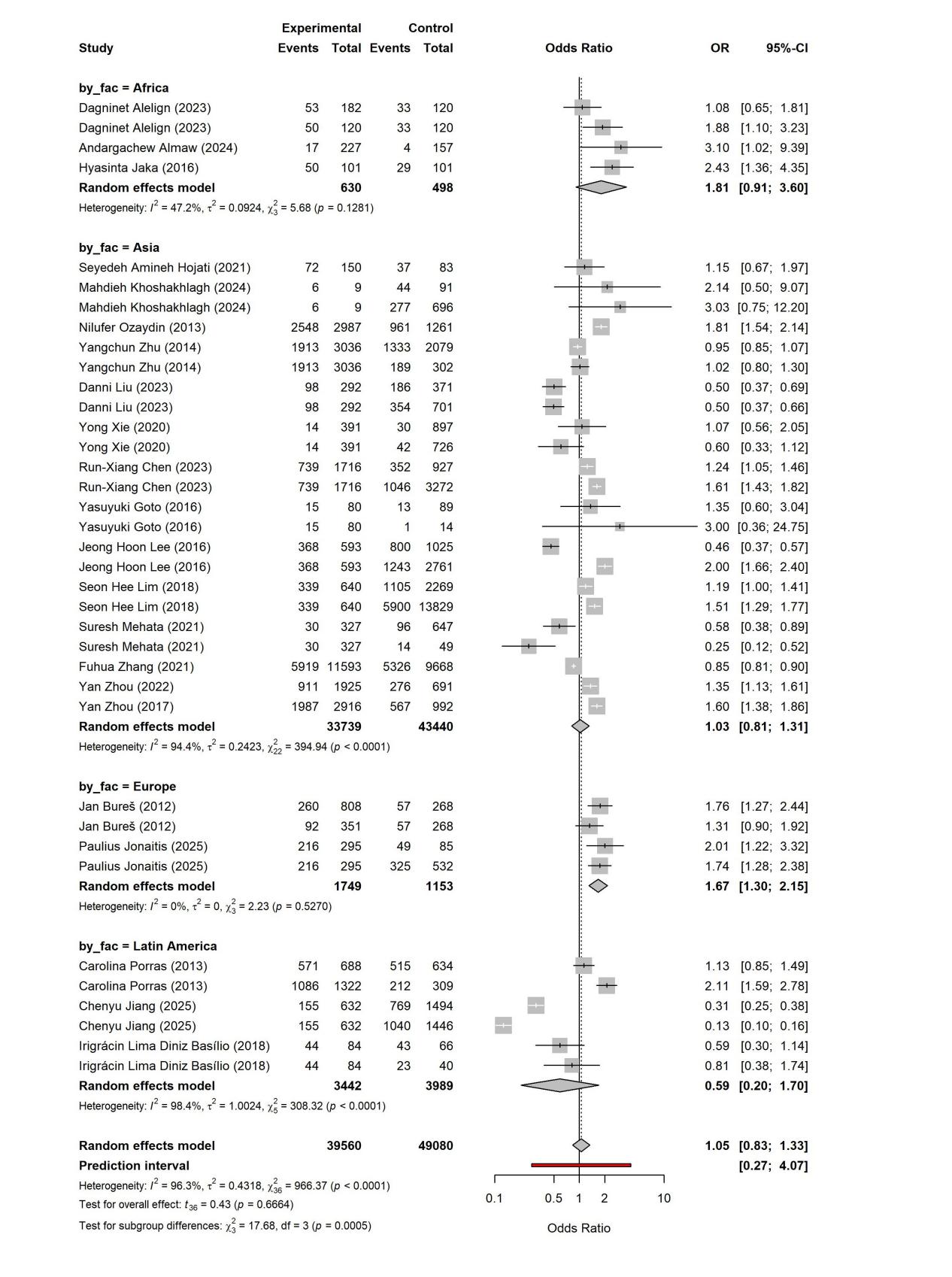


Supplementary Figure 13. Forest plot of education - Helicobacter pylori infection (subgroup: region)


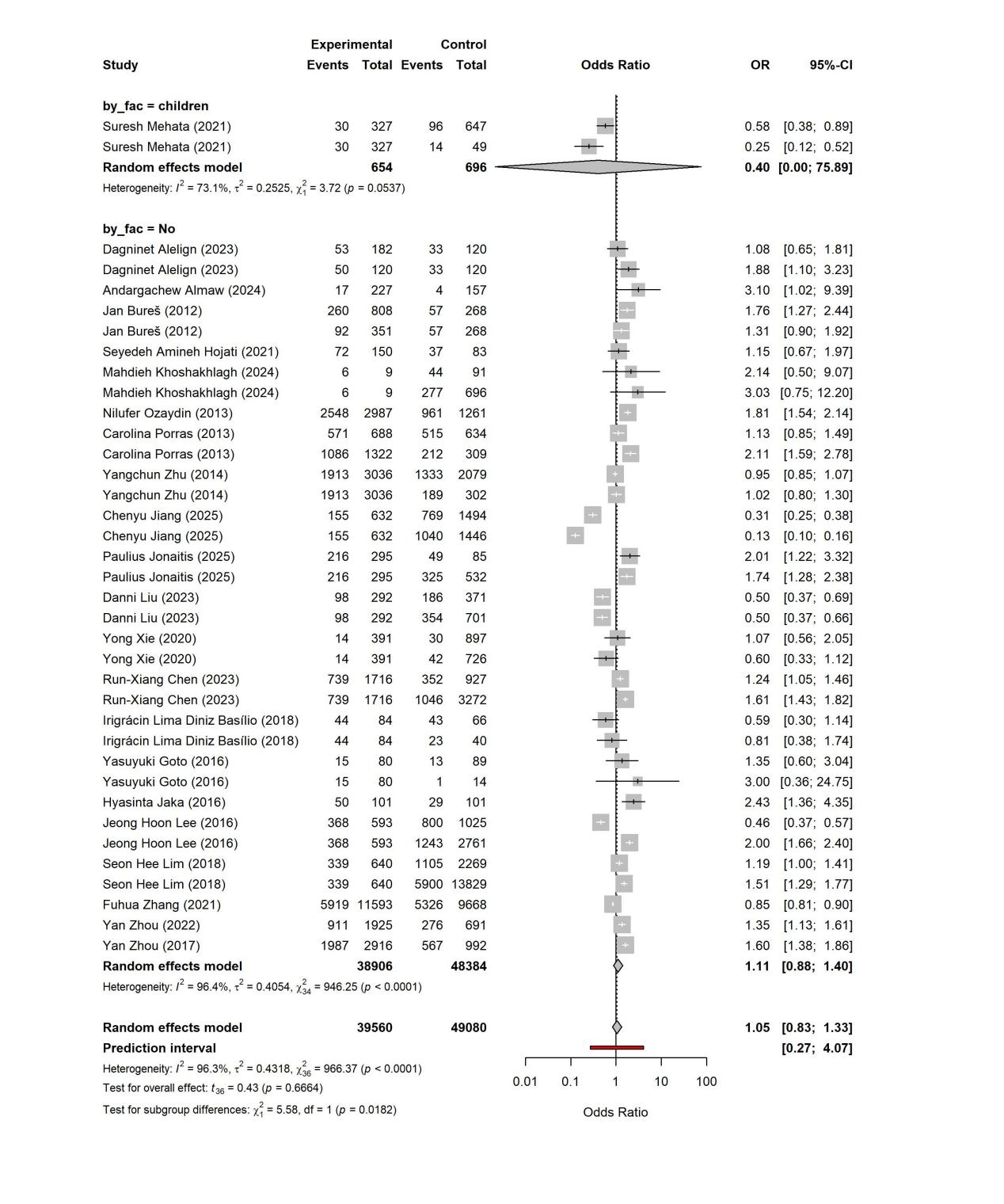


Supplementary Figure 14. Forest plot for education - Helicobacter pylori infection (subgroup: age)


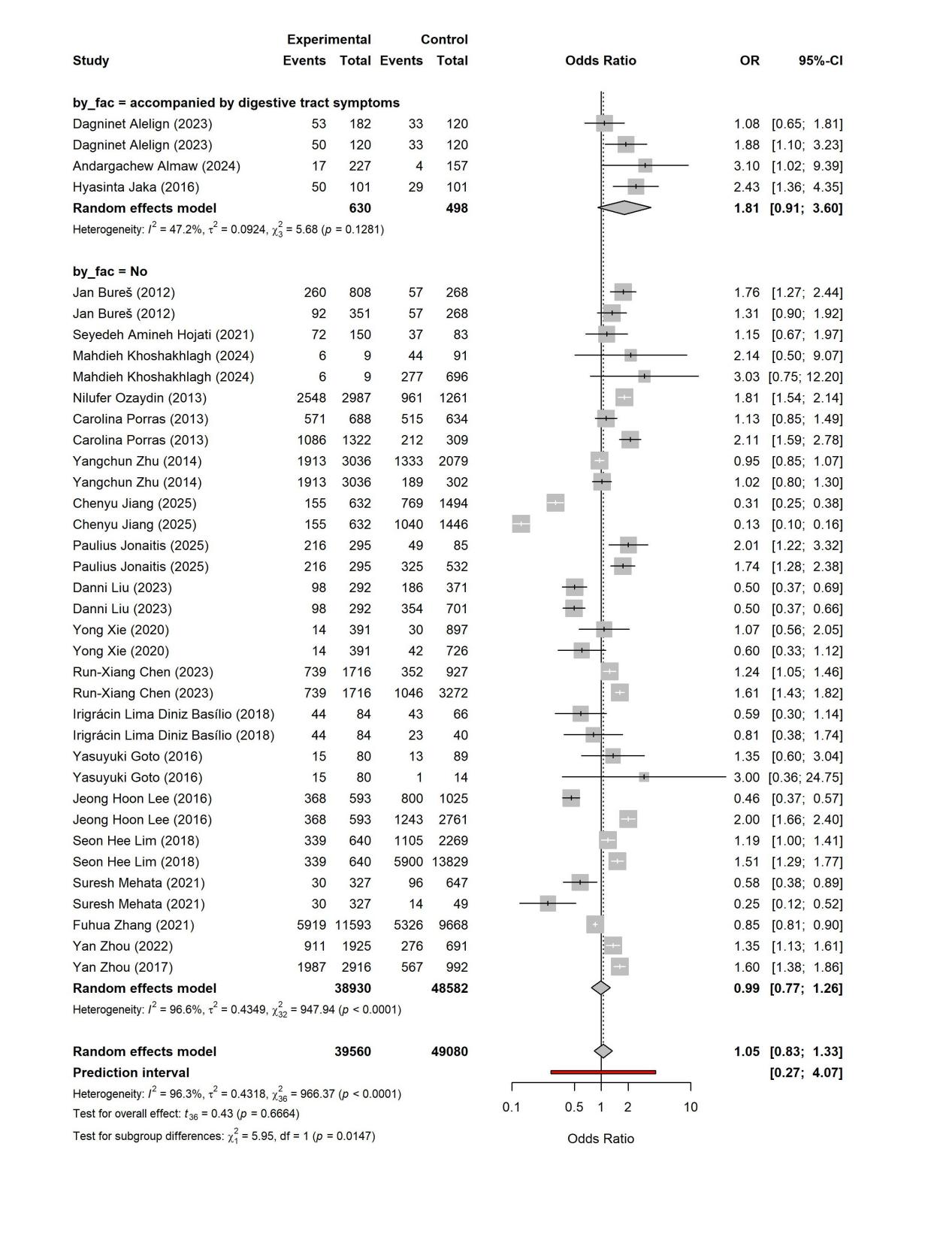


Supplementary Figure 15. Forest plot for education - Helicobacter pylori infection (subgroup: gastrointestinal symptoms)


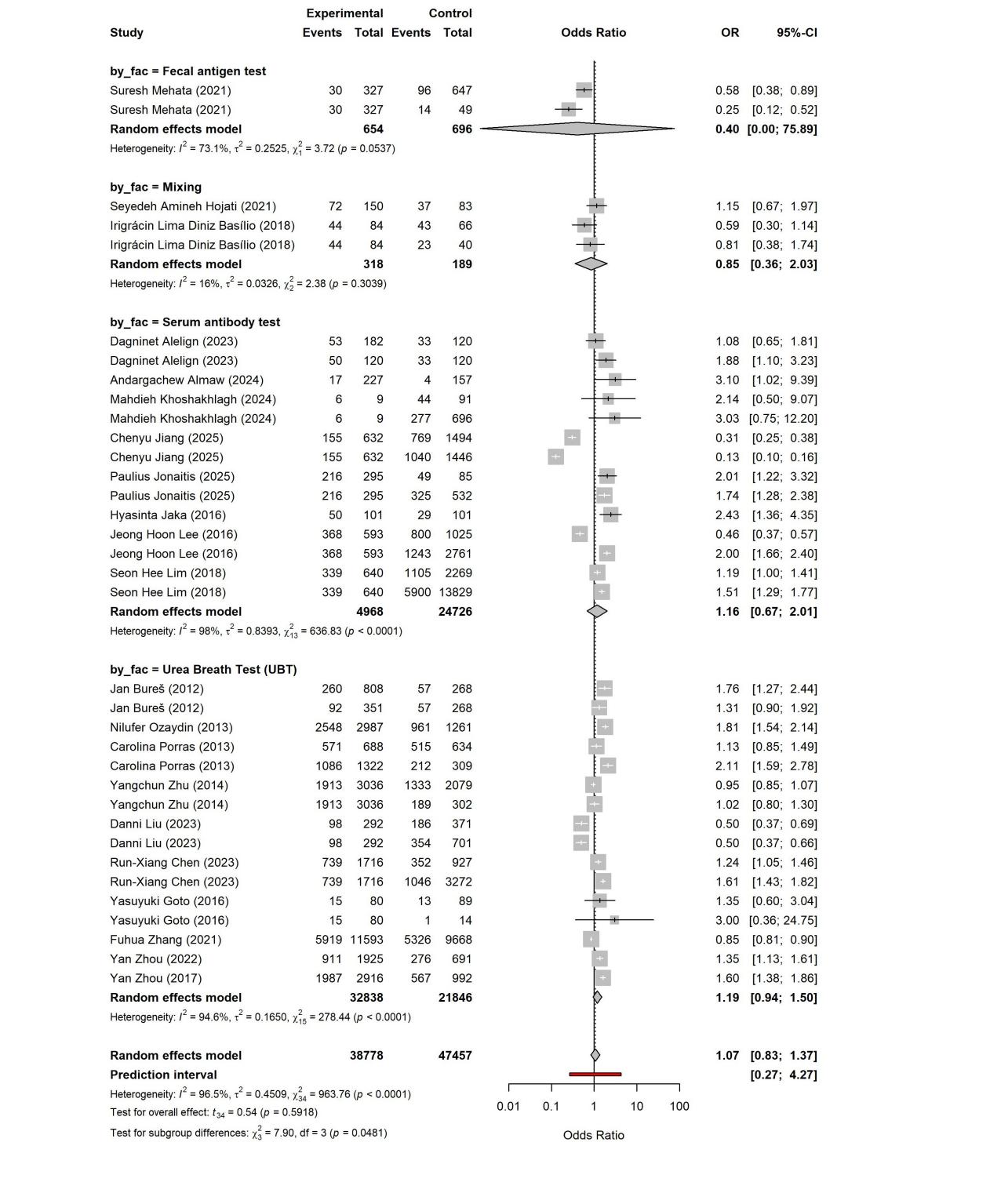


Supplementary Figure 16. Forest plot for education - Helicobacter pylori infection (subgroup: testing method)


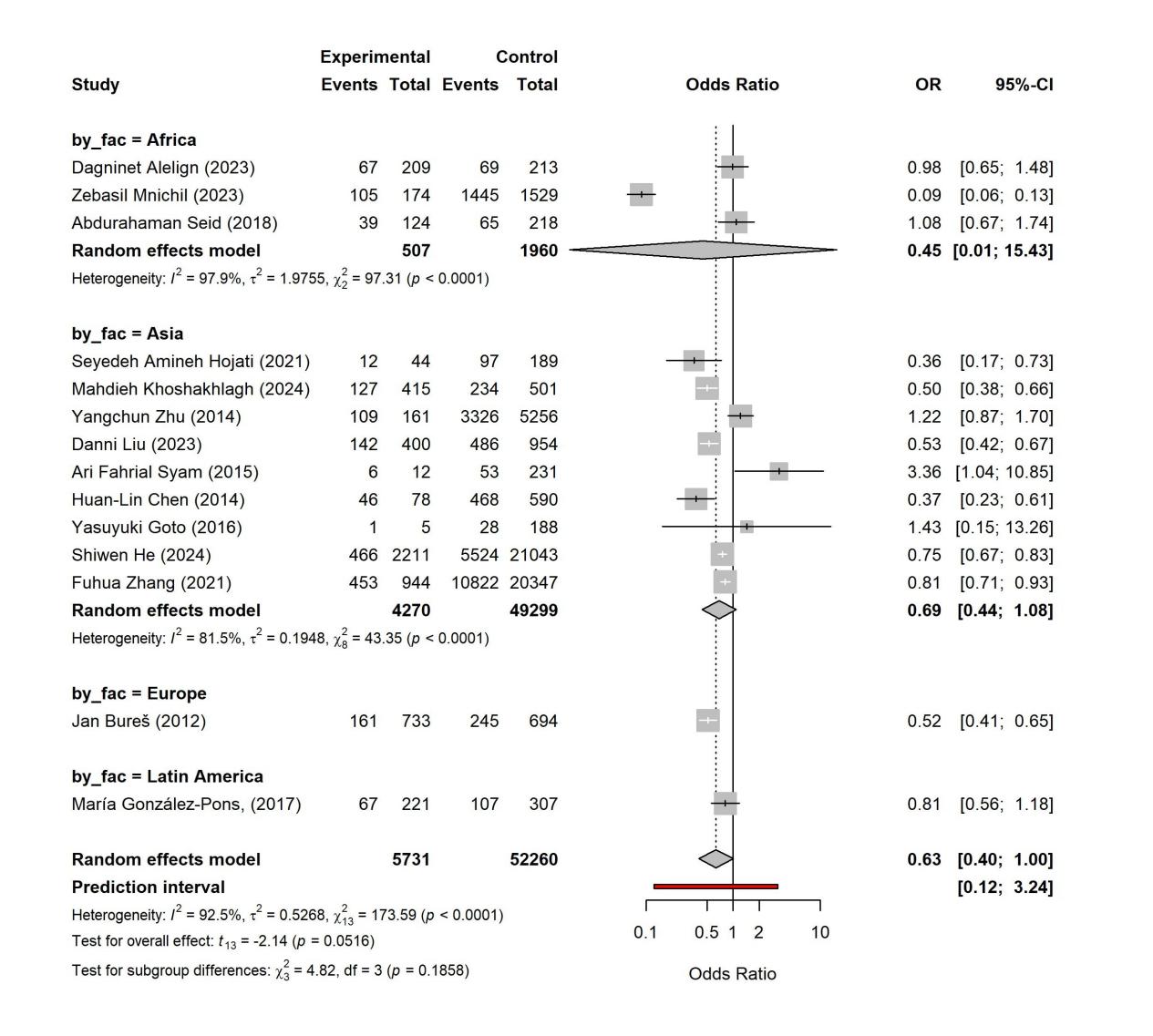


Supplementary Figure 17. Forest plot of marital status - Helicobacter pylori infection (subgroup: region)


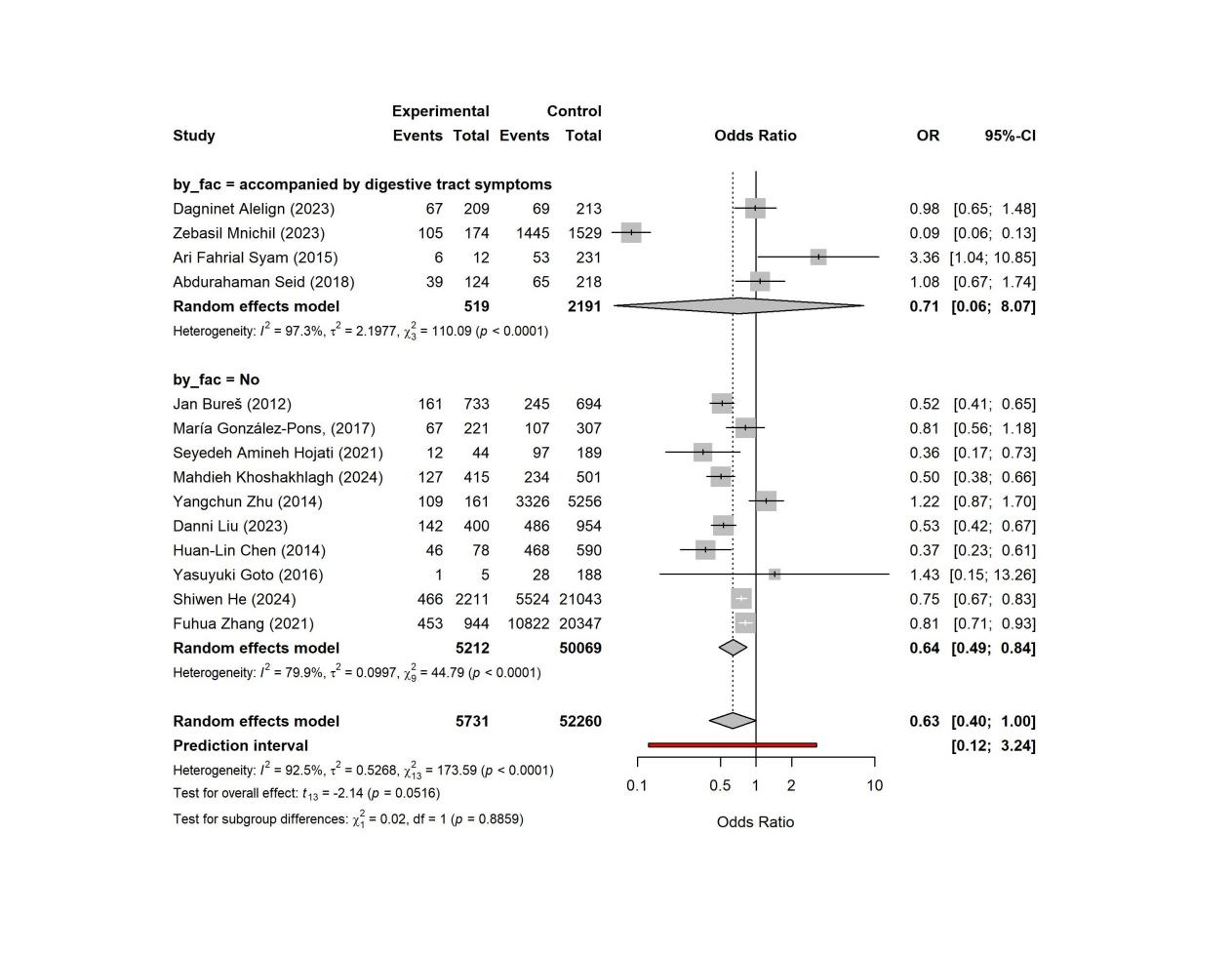


Supplementary Figure 18. Forest plot of marital status - Helicobacter pylori infection (subgroup: gastrointestinal symptoms)


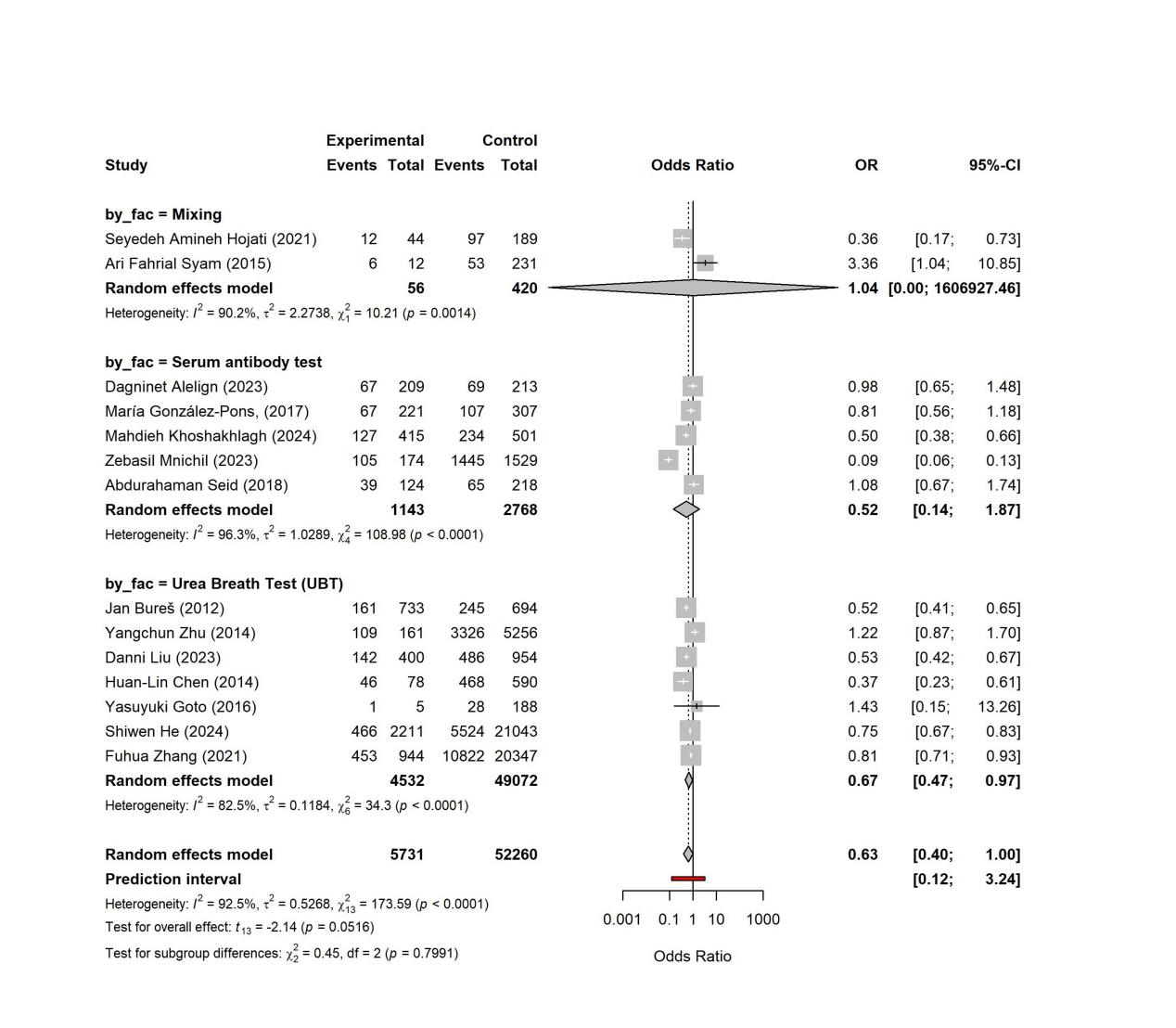


Supplementary Figure 19. Forest plot of marital status - Helicobacter pylori infection (subgroup: testing method)


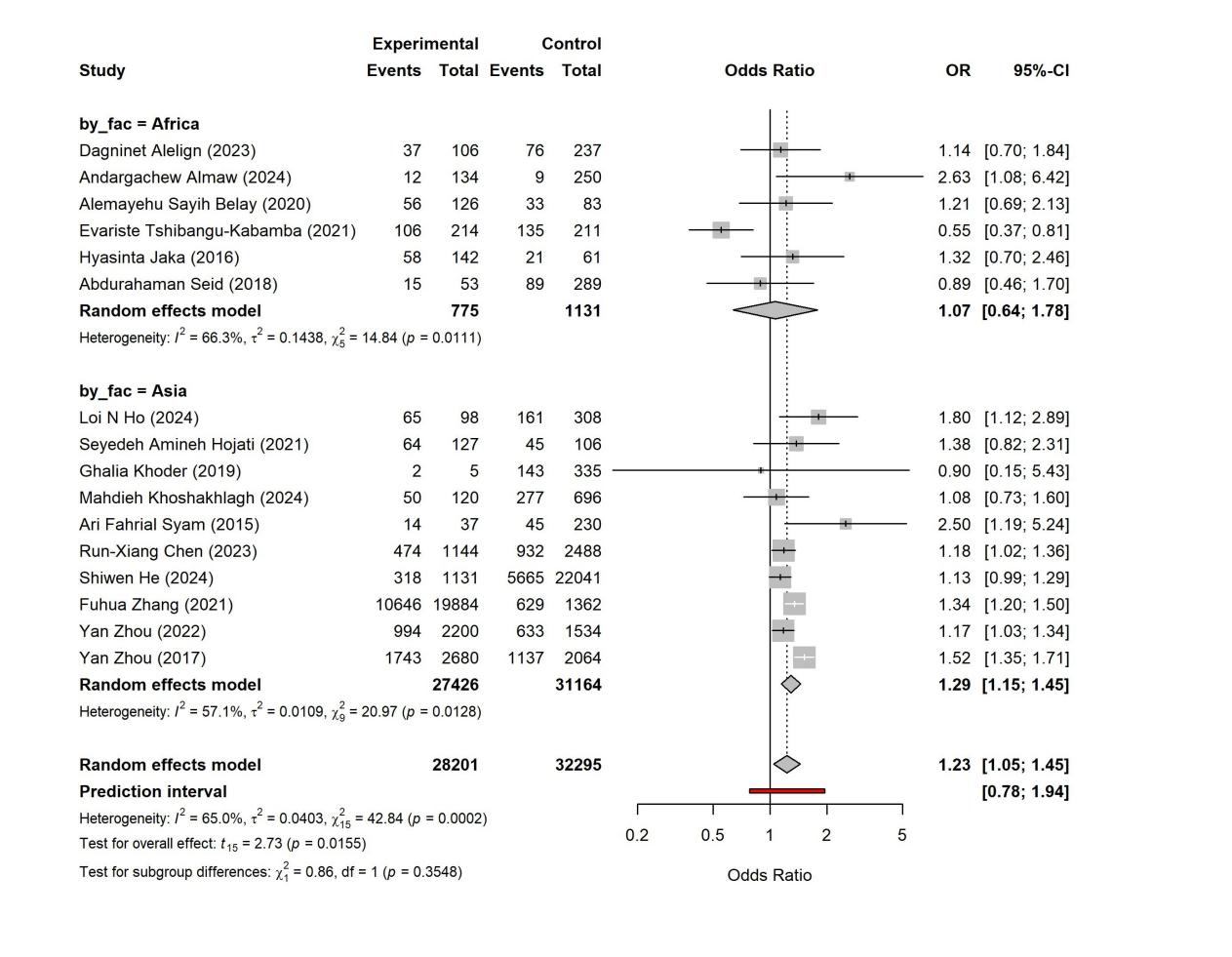


Supplementary Figure 20. Forest plot of marital status - Helicobacter pylori infection (subgroup: region)


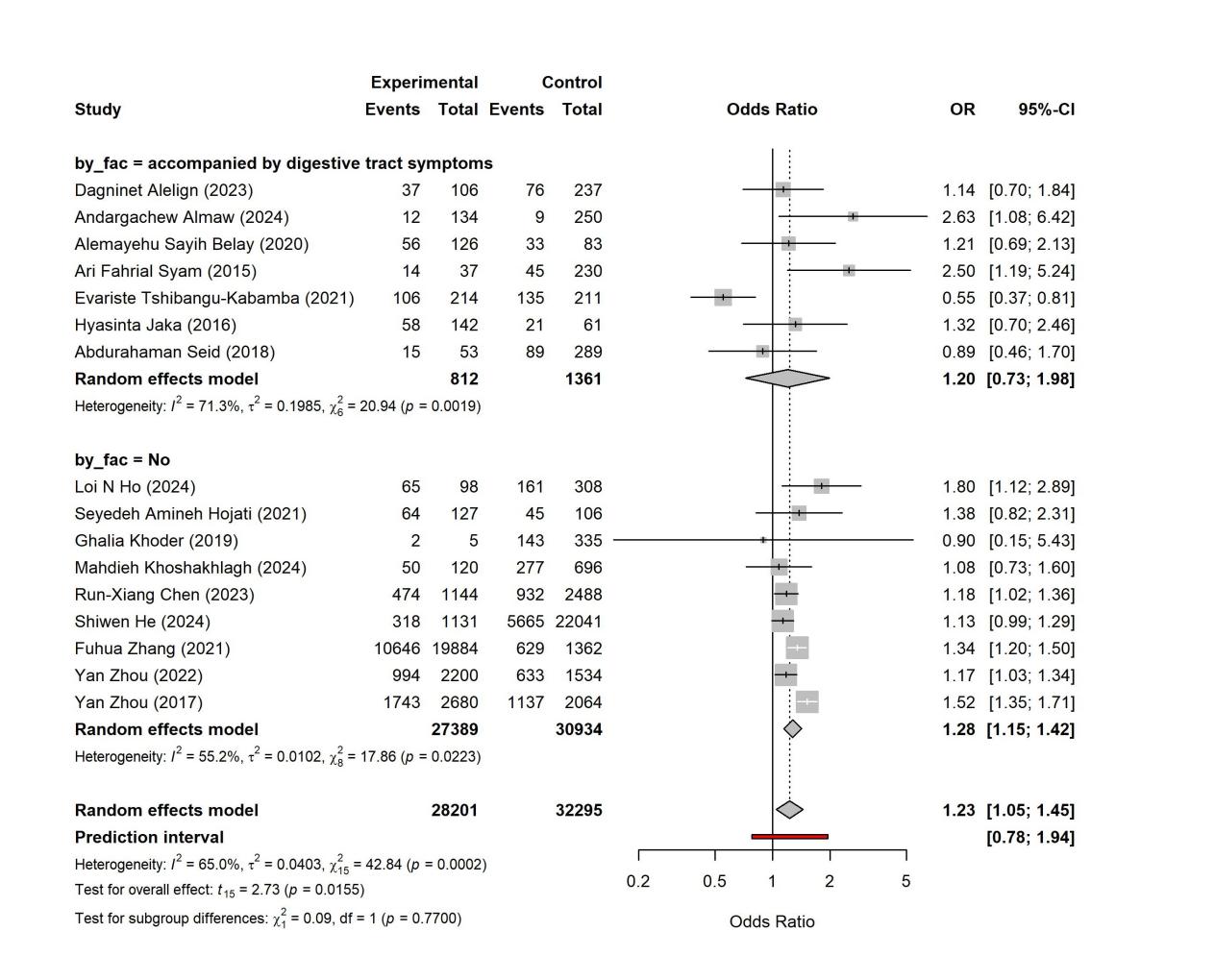


Supplementary Figure 21. Forest plot of marital status - Helicobacter pylori infection (subgroup: gastrointestinal symptoms)


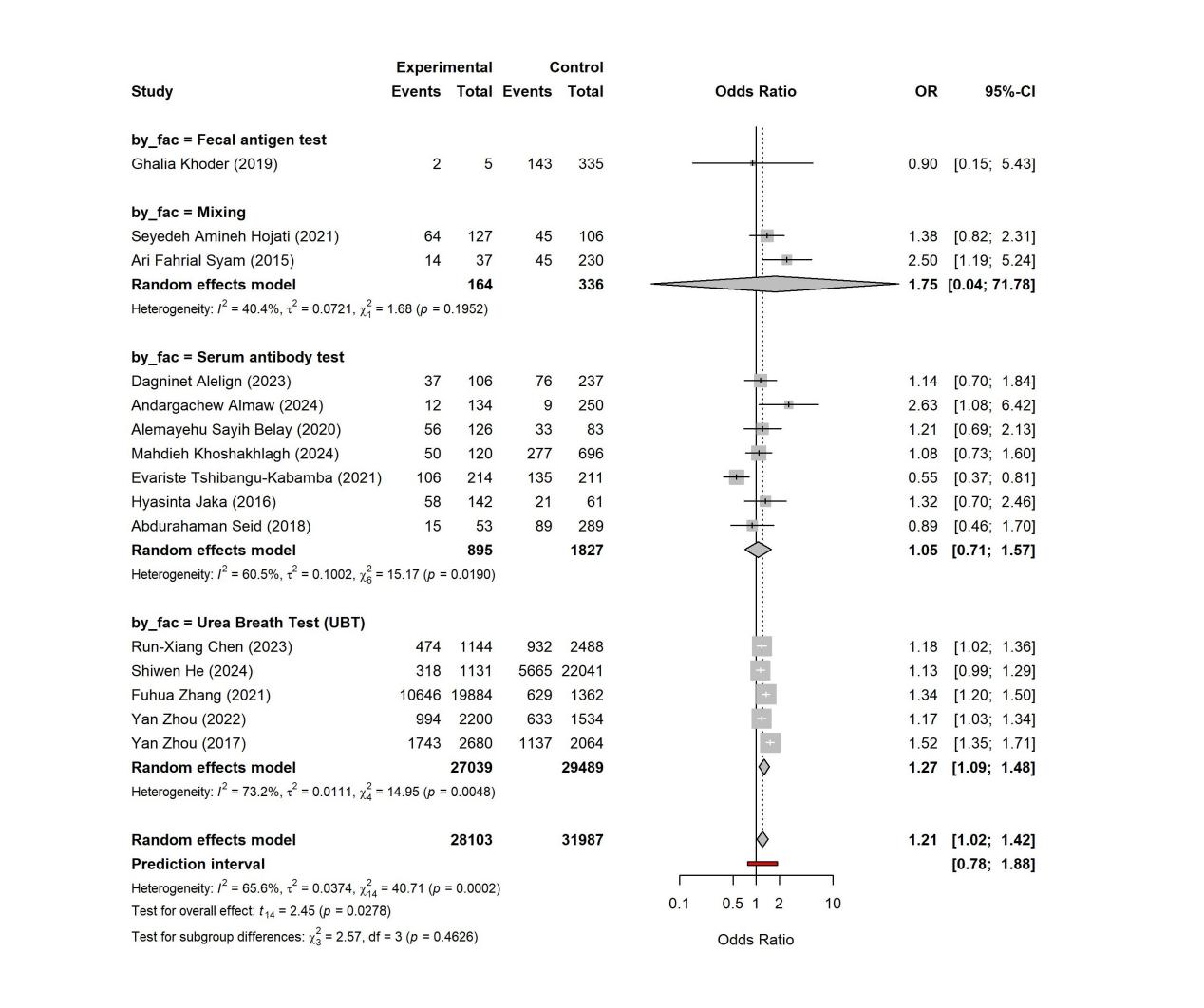


Supplementary Figure 22. Marital Status - Forest Plot for Helicobacter pylori Infection (Subgroup: Testing Method)


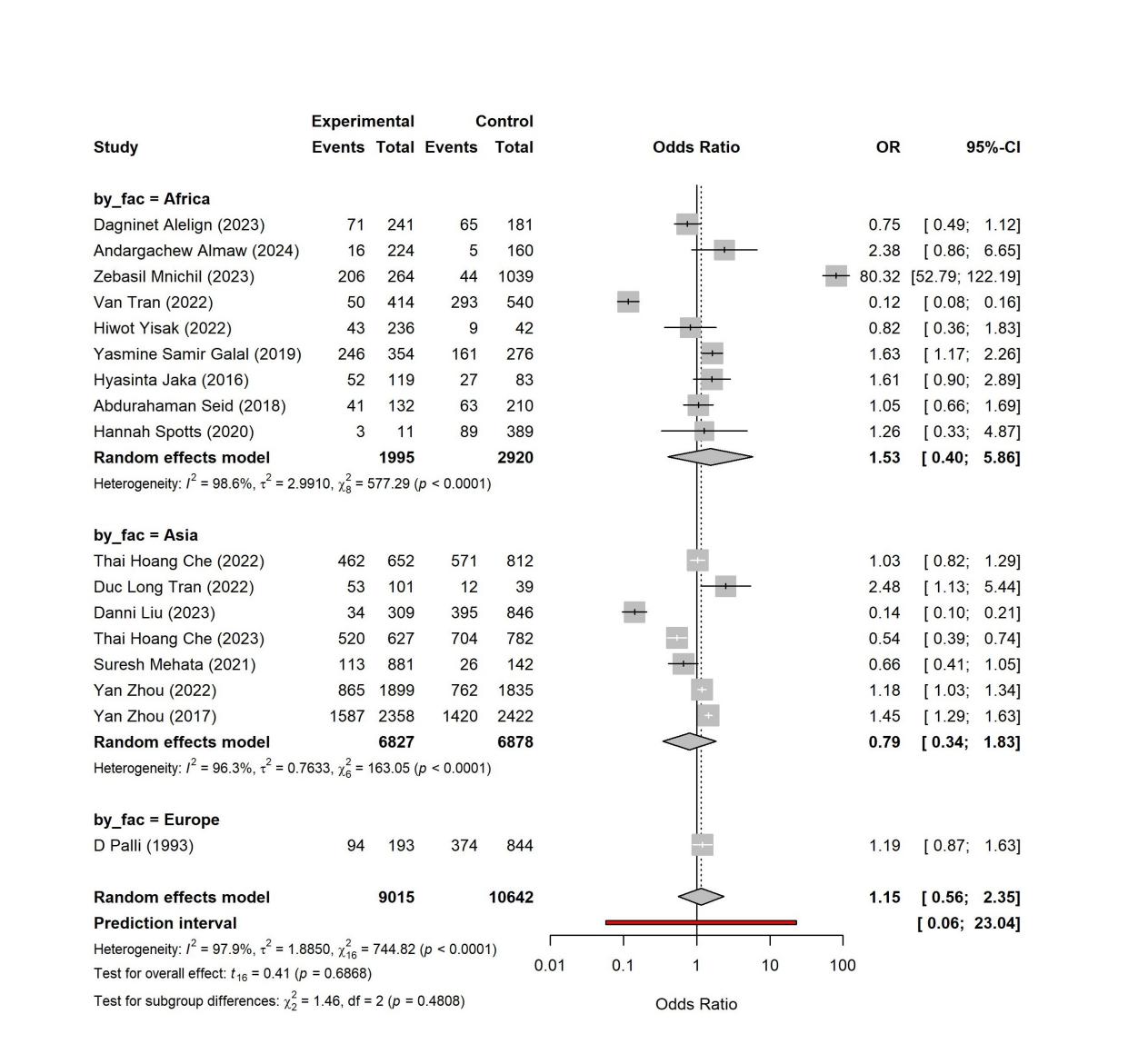


Supplementary Figure 23. Housing Conditions - Forest Plot for Helicobacter pylori Infection (Subgroup: Region)


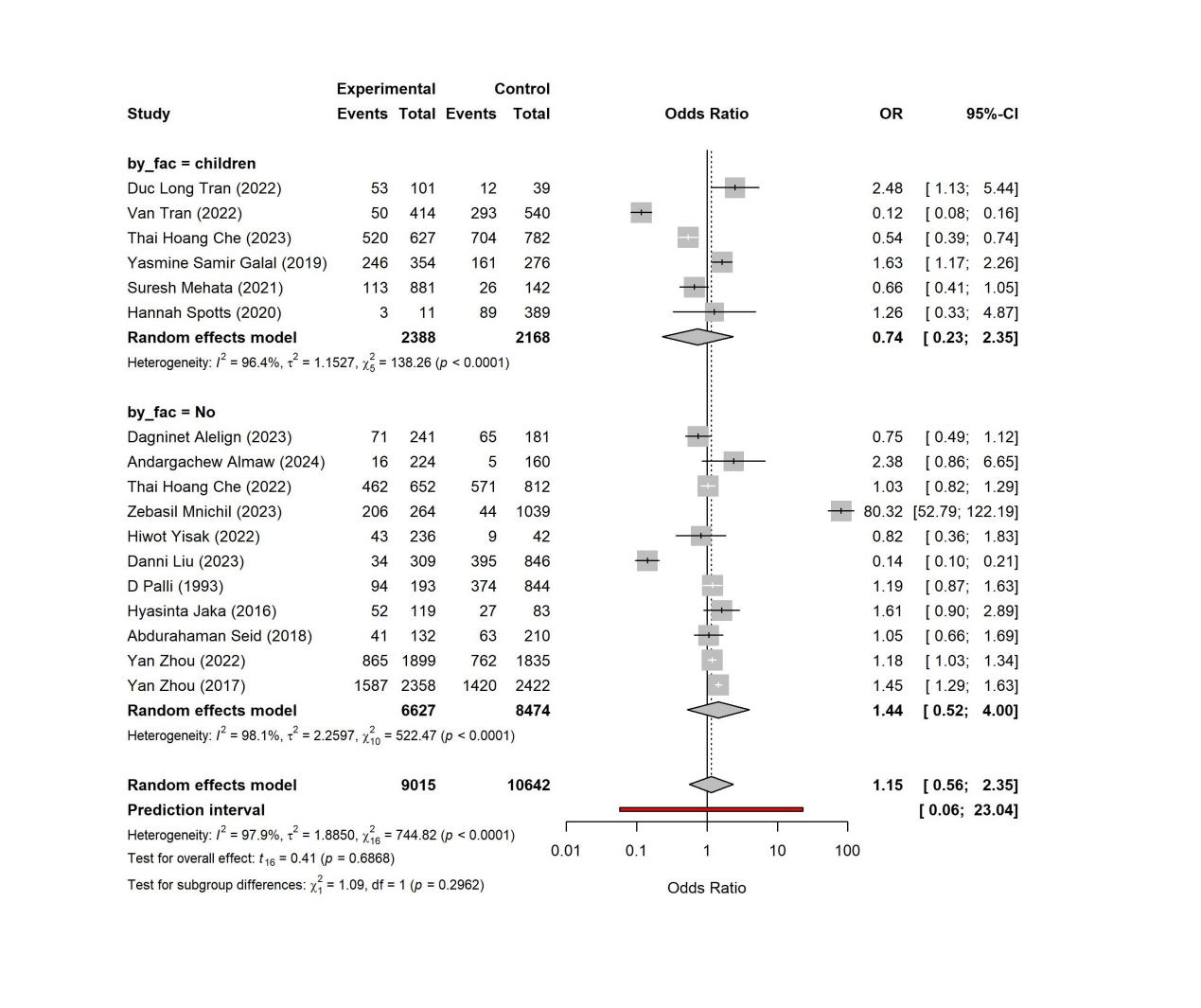


Supplementary Figure 24. Housing Conditions - Forest Plot for Helicobacter pylori Infection (Subgroup: Age)


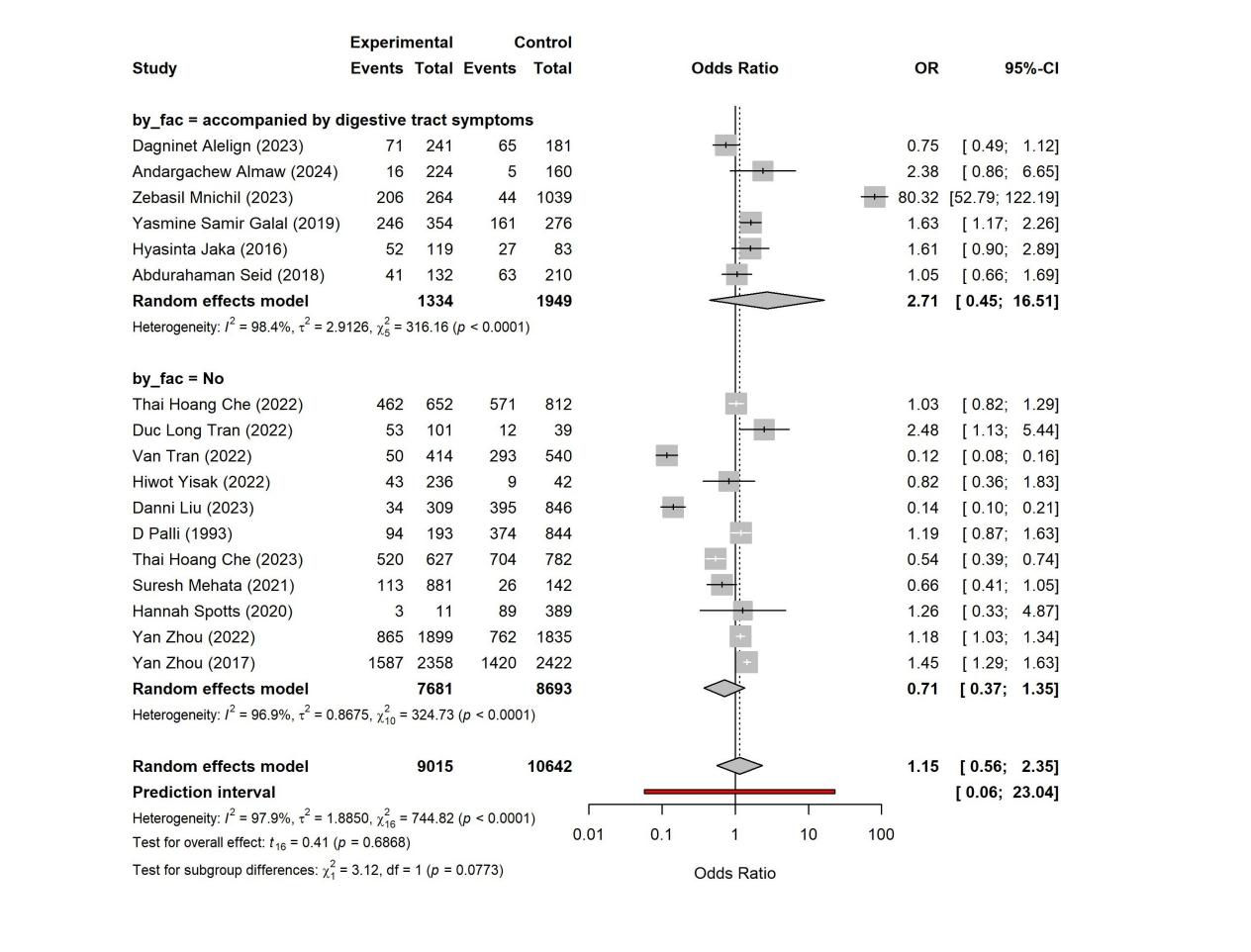


Supplementary Figure 25. Housing Conditions - Forest Plot for Helicobacter pylori Infection (Subgroup: With Gastrointestinal Symptoms)


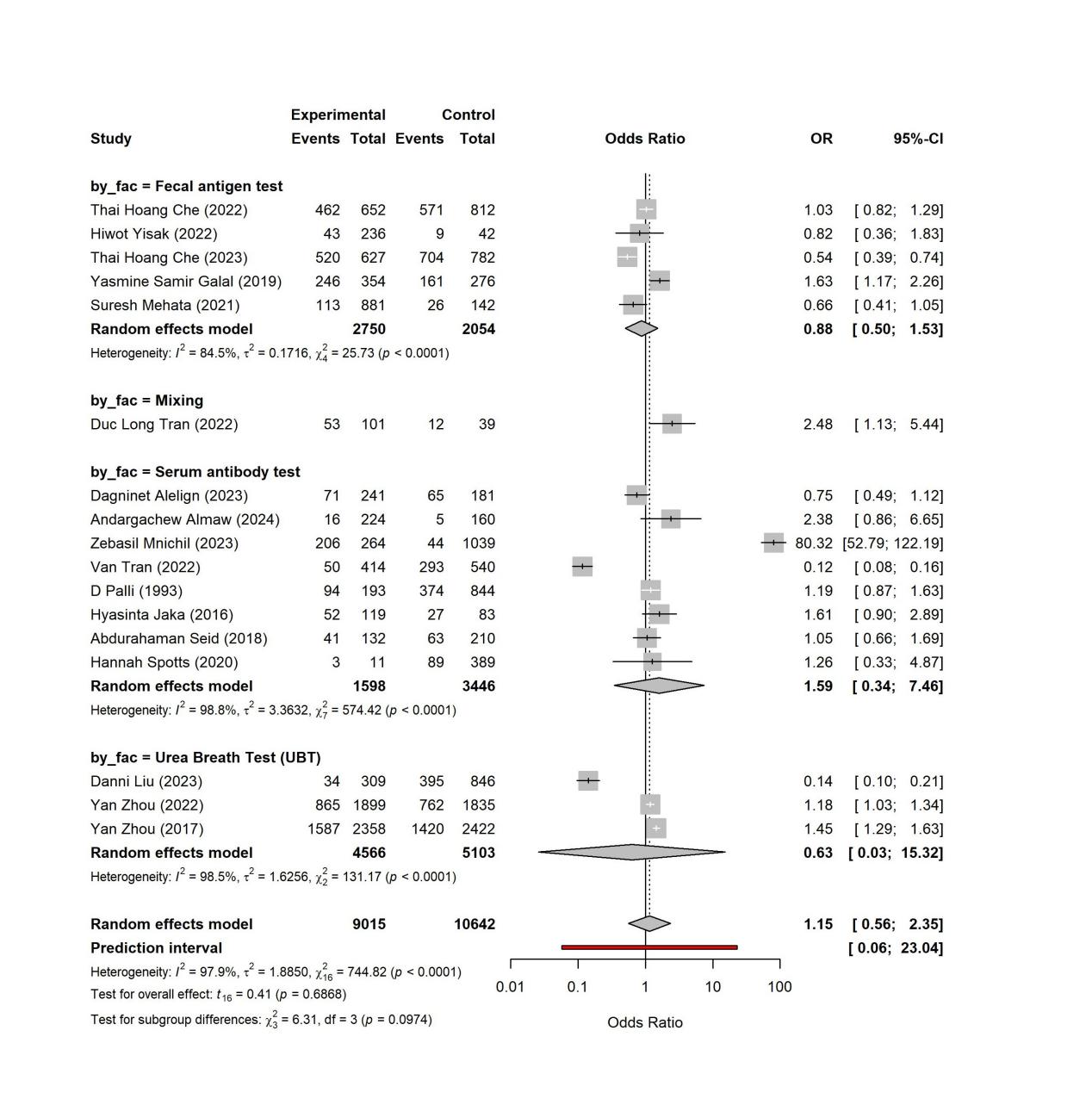


Supplementary Figure 26. Housing Conditions - Forest Plot for Helicobacter pylori Infection (Subgroup: Testing Method)


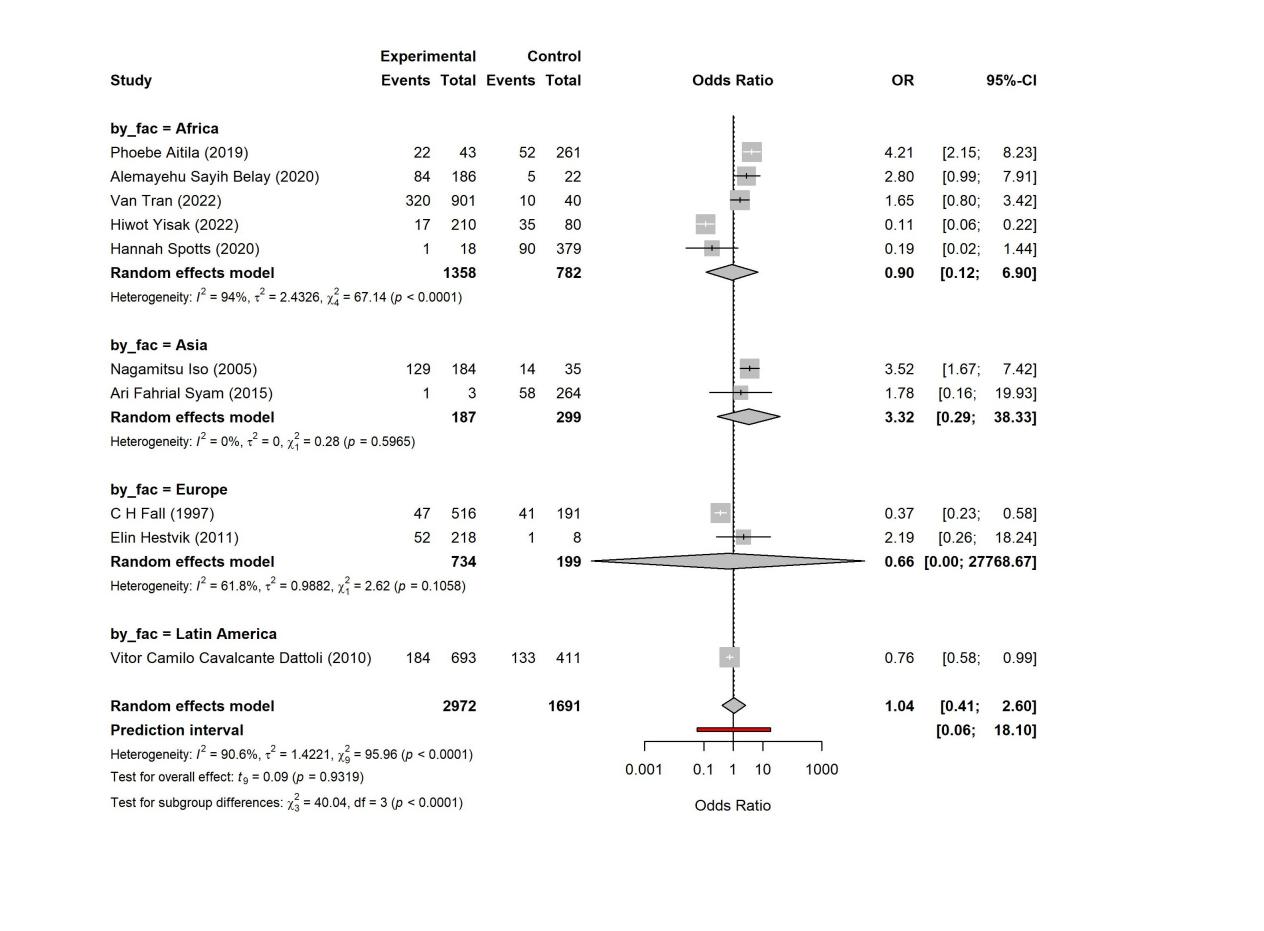


Supplementary Figure 27. Health Conditions - Forest Plot for Helicobacter pylori Infection (Subgroup: Region)


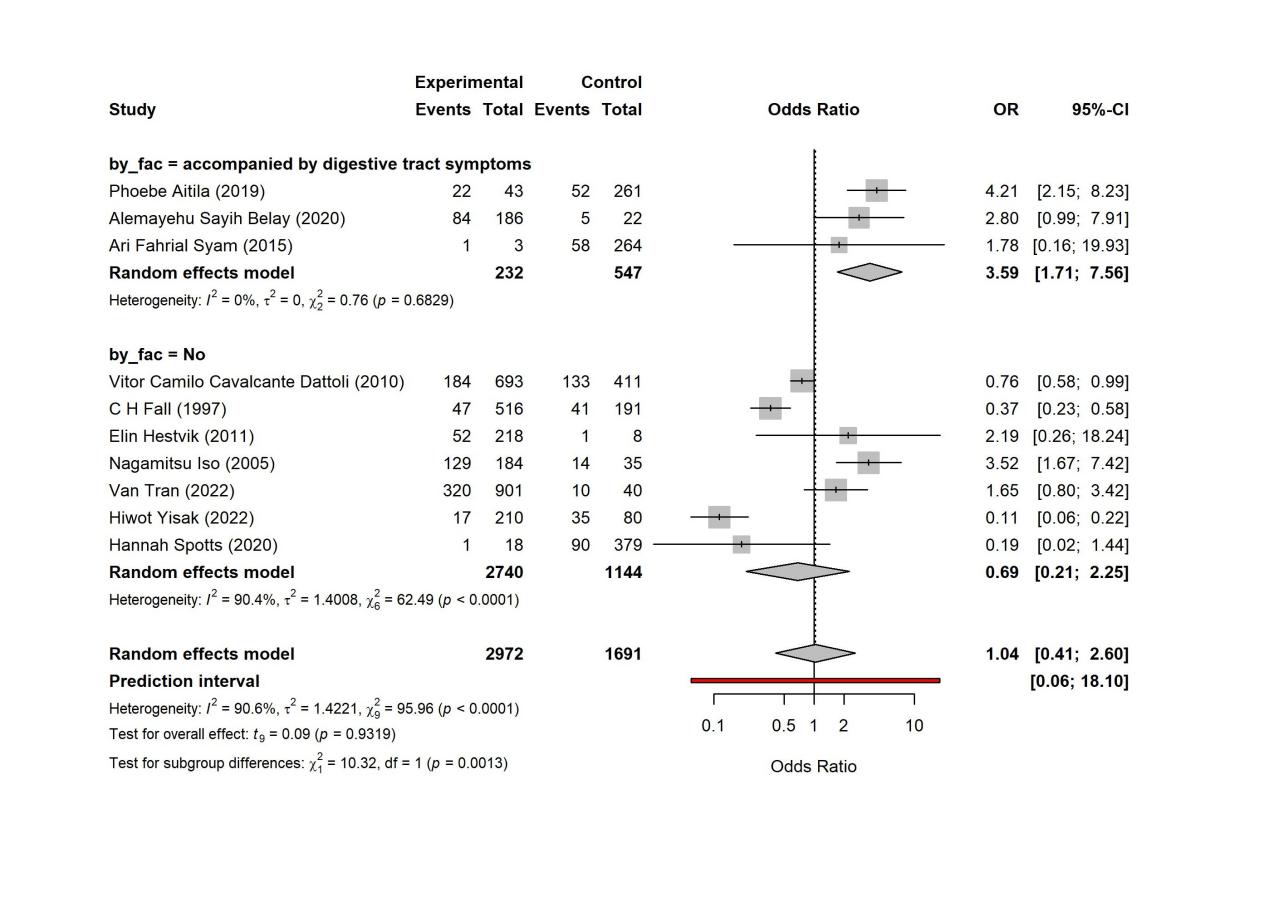


Supplementary Figure 28. Forest plot for hygiene conditions - Helicobacter pylori infection (Subgroup: Gastrointestinal symptoms)


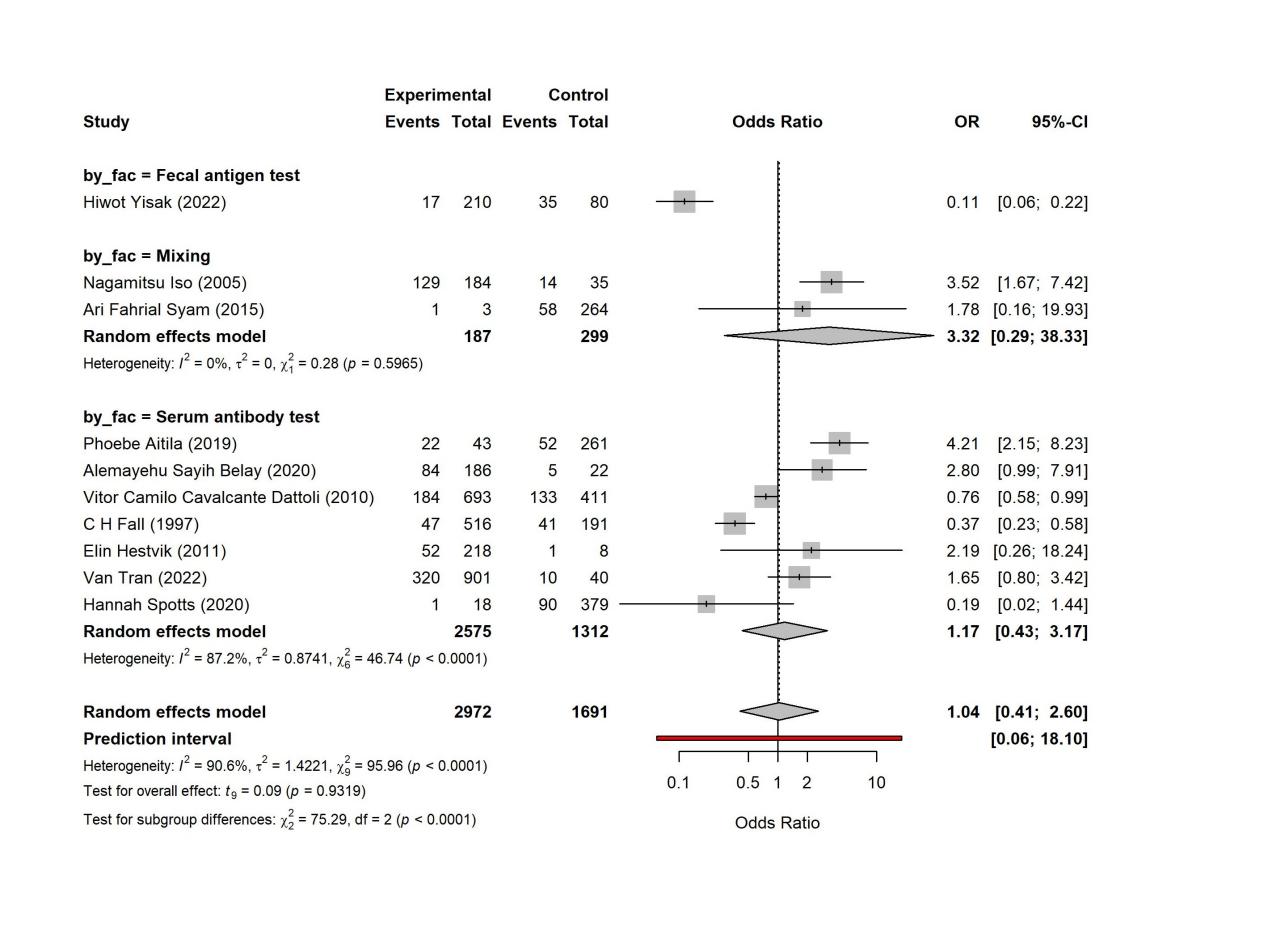


Supplementary Figure 29. Hygiene Conditions - Forest Plot for Helicobacter pylori Infection (Subgroup: Testing Method)


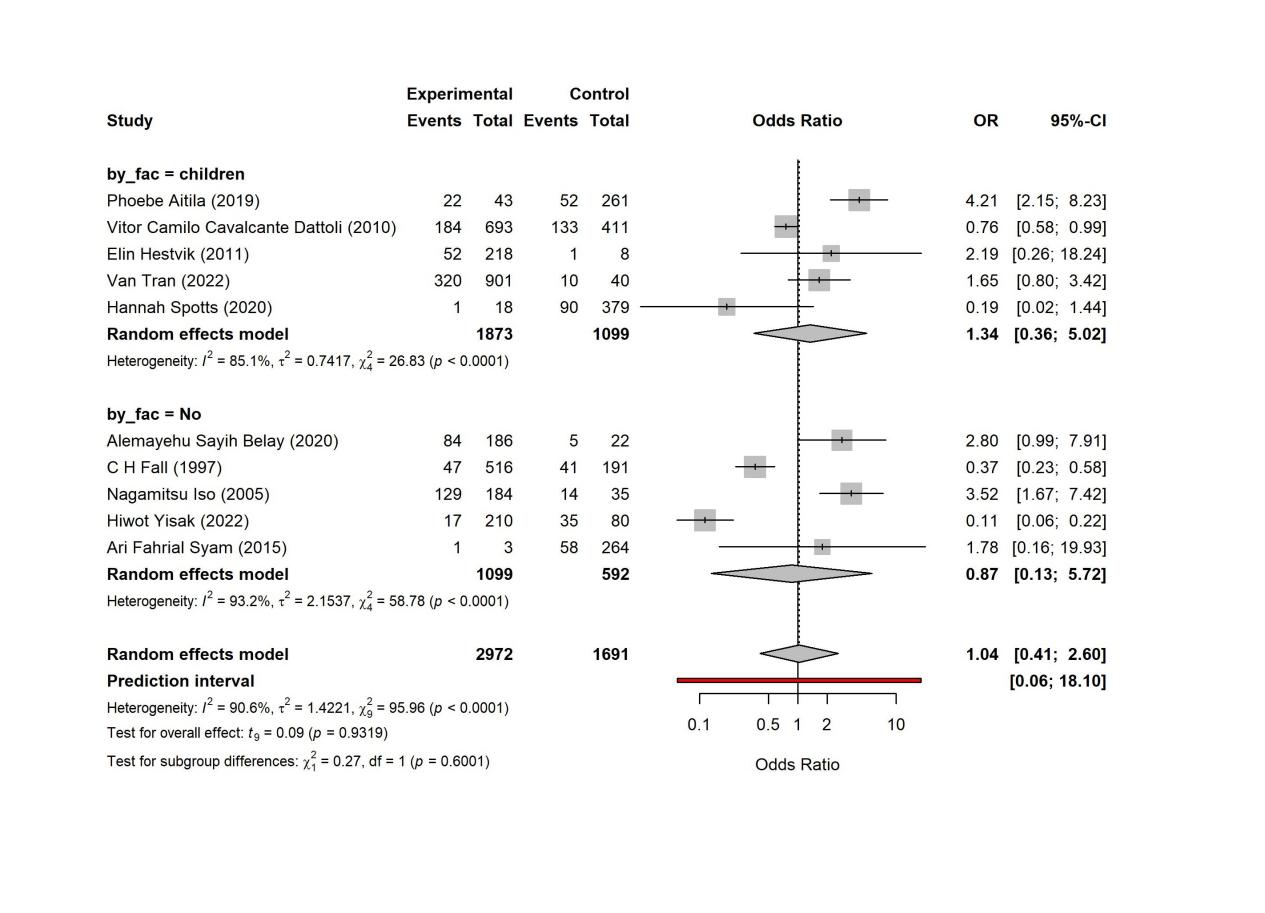


Supplementary Figure 30. Forest plot of hygiene conditions - Helicobacter pylori infection (subgroup: age)


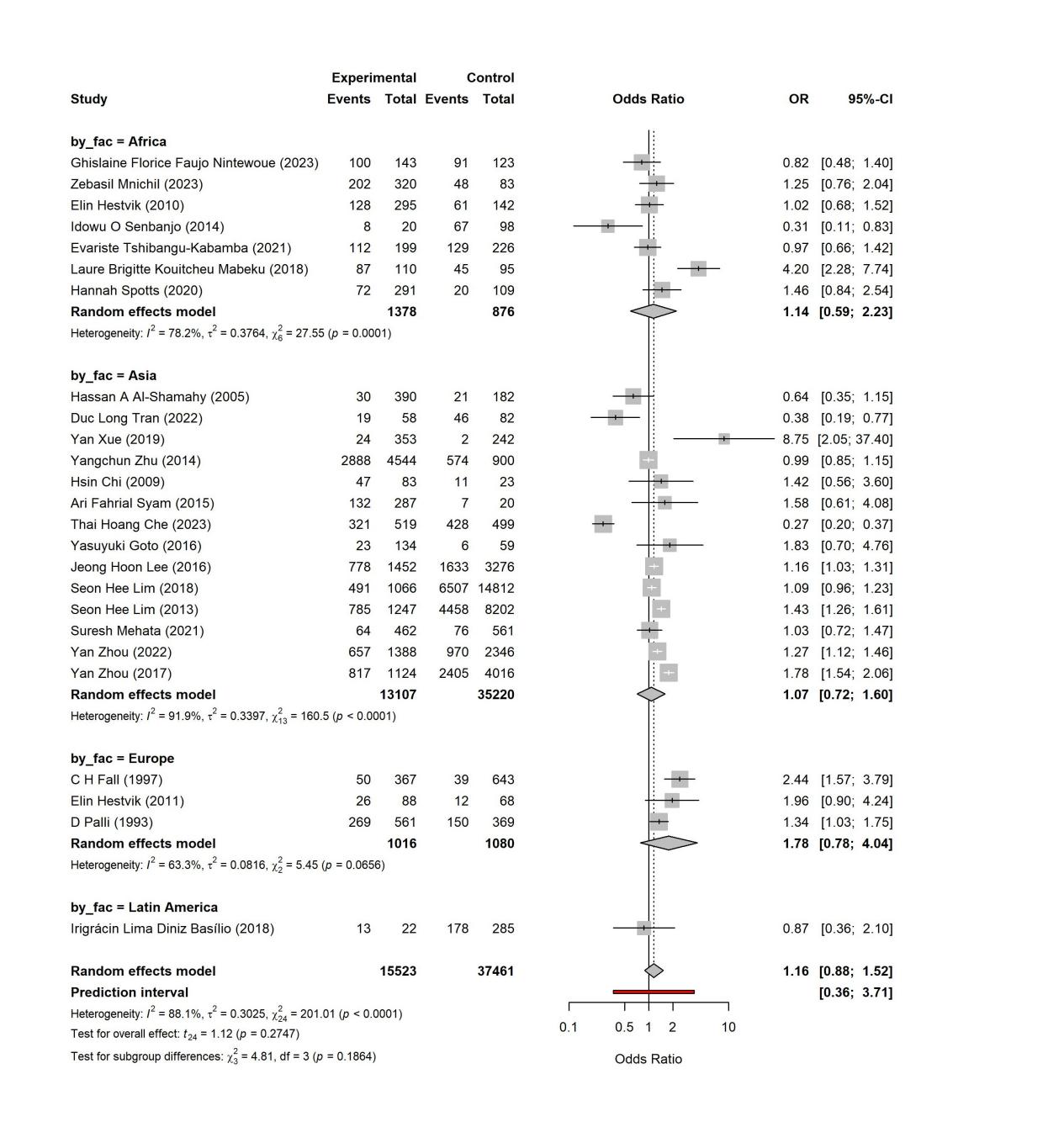


Supplementary Figure 31. Socio-economic Conditions - Forest Plot for Helicobacter pylori Infection (Subgroup: Region)


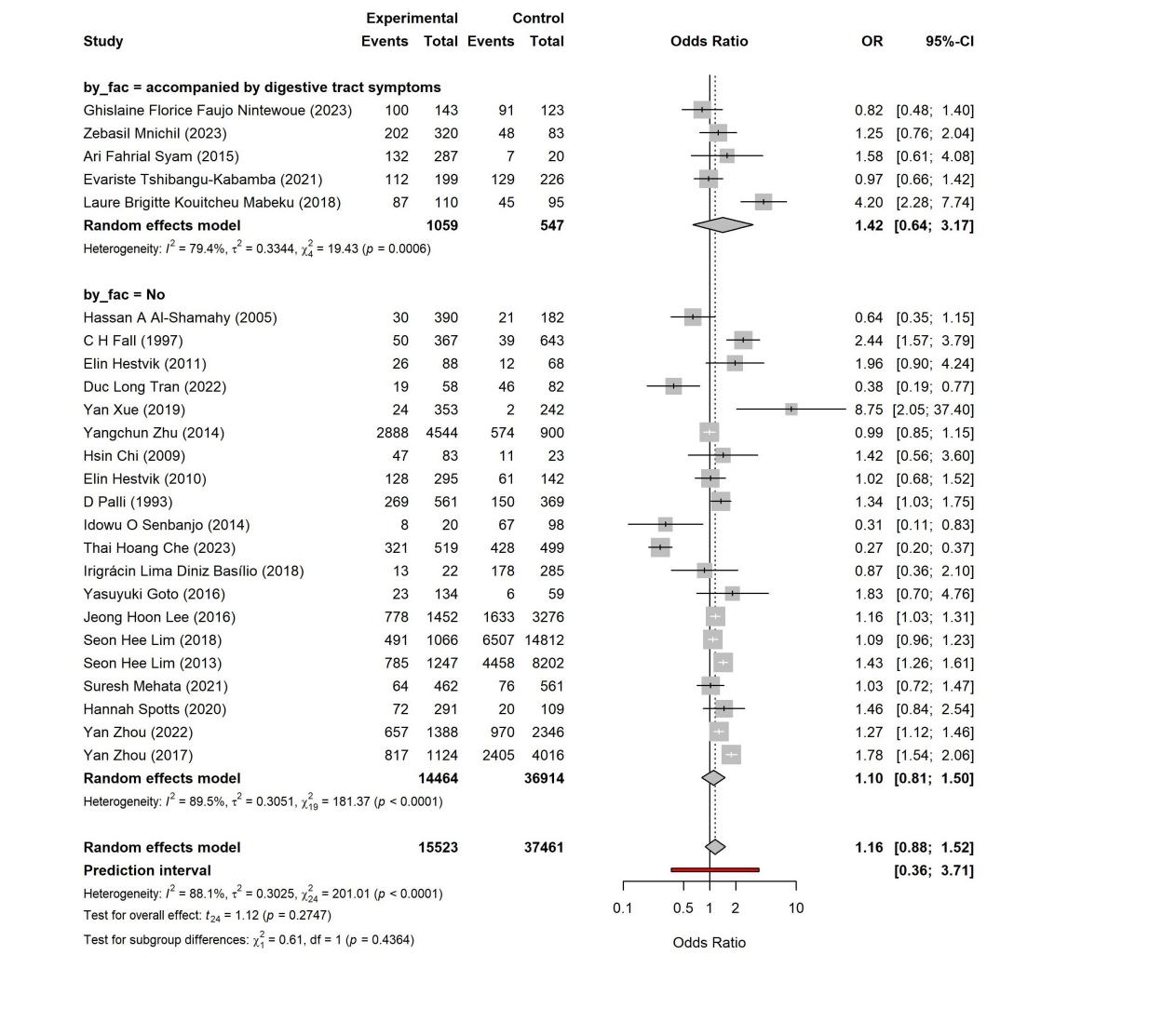


Supplementary Figure 32. Socioeconomic Factors - Forest Plot for Helicobacter pylori Infection (Subgroup: Gastrointestinal Symptoms)


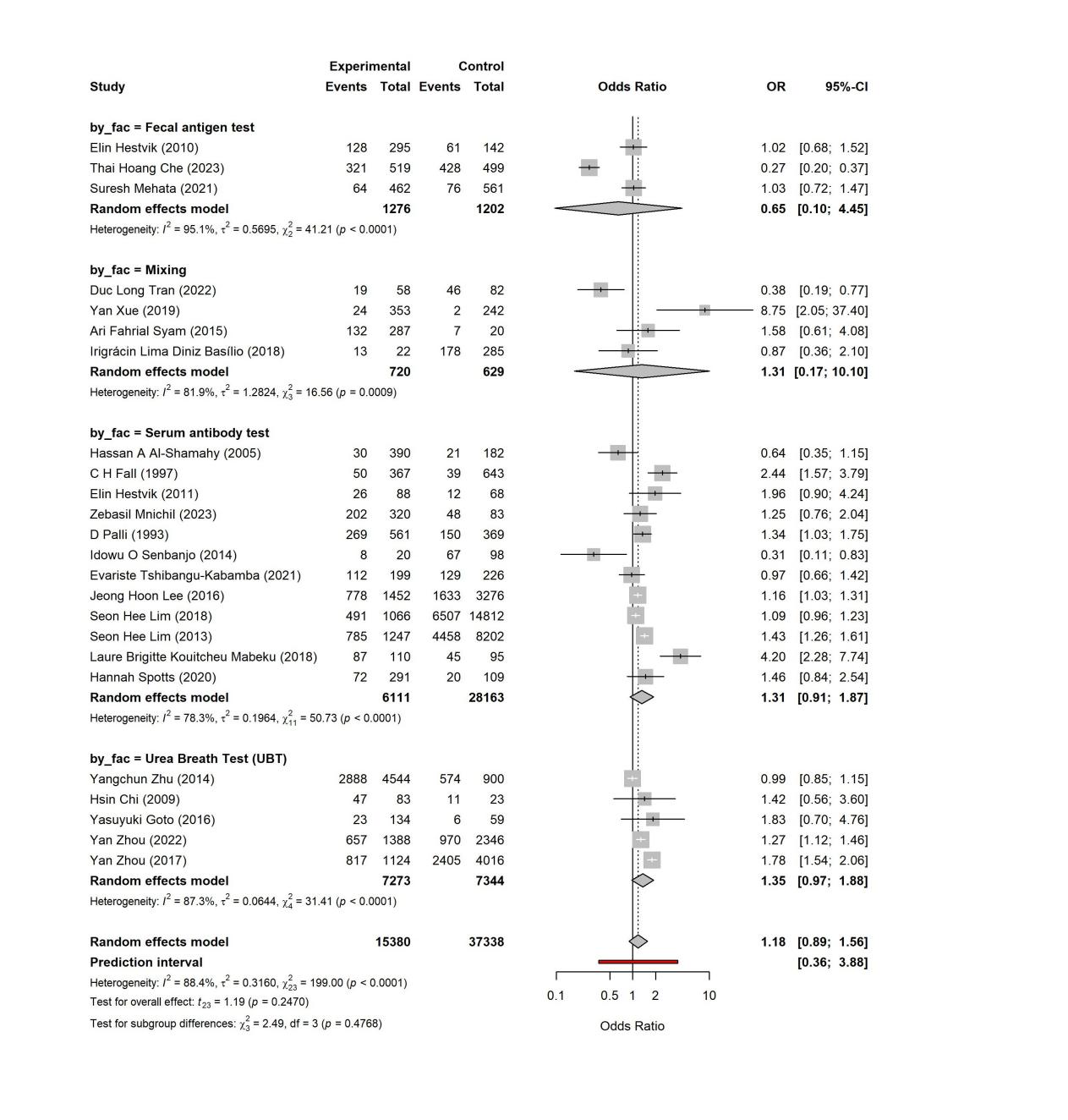


Supplementary Figure 33. Socioeconomic Conditions - Forest Plot for Helicobacter pylori Infection (Subgroup: Diagnostic Methods)


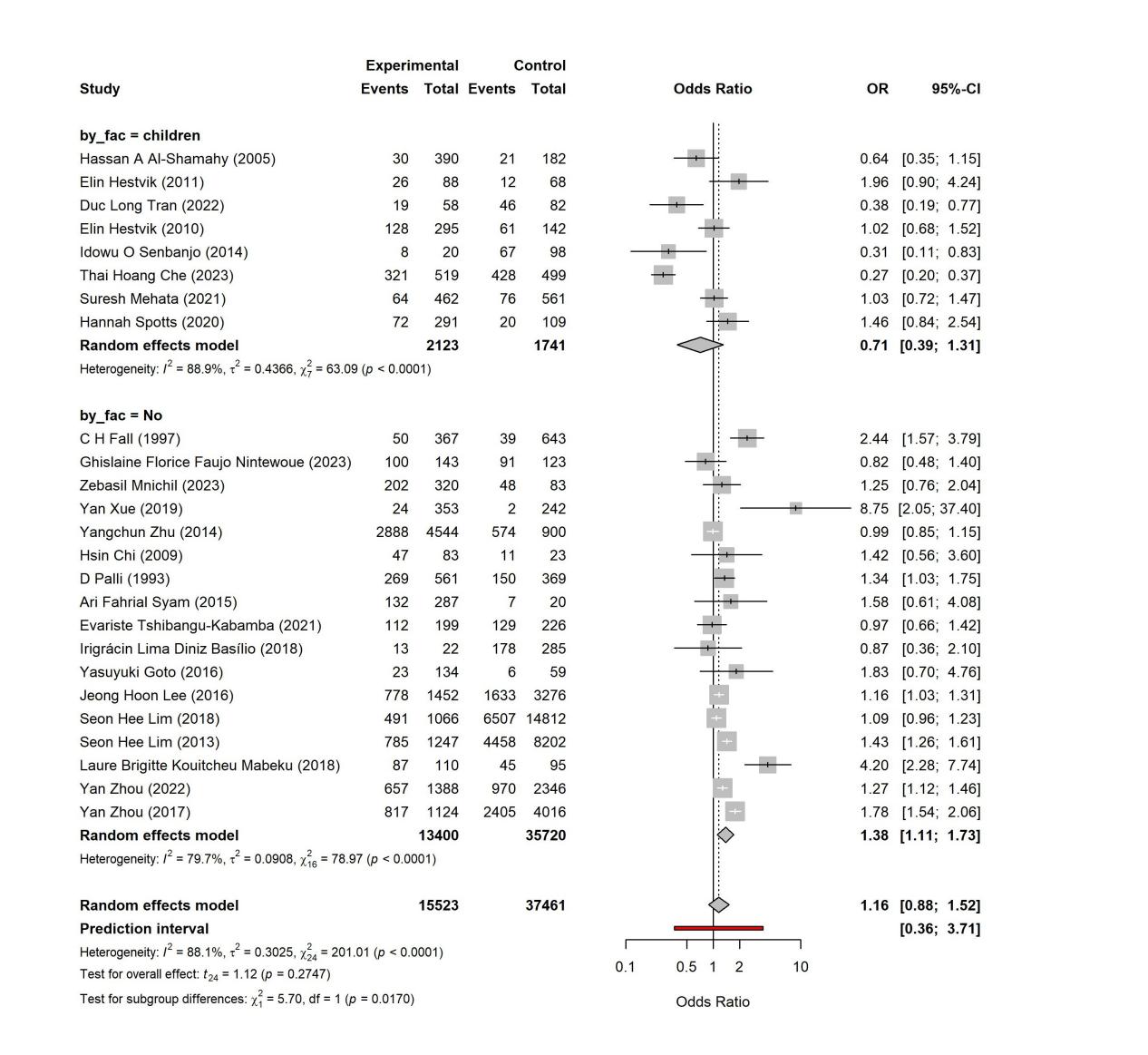


Supplementary Figure 34. Social and Economic Conditions - Forest Plot for Helicobacter pylori Infection (Subgroup: Age)
